# Repression of GSK3 restores NK cell cytotoxicity in AML patients

**DOI:** 10.1038/ncomms11154

**Published:** 2016-04-04

**Authors:** Reshmi Parameswaran, Parameswaran Ramakrishnan, Stephen A. Moreton, Zhiqiang Xia, Yongchun Hou, Dean A. Lee, Kalpana Gupta, Marcos deLima, Rose C. Beck, David N. Wald

**Affiliations:** 1 Department of Pathology, Case Western Reserve University, Cleveland, Ohio 44106, USA; 2 Invenio Therapeutics, Lexington, Kentucky 40506, USA; 3 Division of pediatrics, The University of Texas Health Sciences Center, MD Anderson Cancer Center, Houston, Texas 77030, USA; 4 Department of Medicine, University Hospitals Case Medical Center, Cleveland, Ohio 44106, USA; 5 Department of Pathology, University Hospitals Case Medical Center, Cleveland, Ohio 44106, USA

## Abstract

Natural killer cells from acute myeloid leukaemia patients (AML-NK) show a dramatic impairment in cytotoxic activity. The exact reasons for this dysfunction are not fully understood. Here we show that the glycogen synthase kinase beta (GSK3β) expression is elevated in AML-NK cells. Interestingly, GSK3 overexpression in normal NK cells impairs their ability to kill AML cells, while genetic or pharmacological GSK3 inactivation enhances their cytotoxic activity. Mechanistic studies reveal that the increased cytotoxic activity correlates with an increase in AML-NK cell conjugates. GSK3 inhibition promotes the conjugate formation by upregulating LFA expression on NK cells and by inducing ICAM-1 expression on AML cells. The latter is mediated by increased NF-κB activation in response to TNF-α production by NK cells. Finally, GSK3-inhibited NK cells show significant efficacy in human AML mouse models. Overall, our work provides mechanistic insights into the AML-NK dysfunction and a potential NK cell therapy strategy.

## Abstract (web summary)

Natural killer cells of acute myeloid leukaemia patients lack cytotoxic activity. Here the authors show that these cells have elevated GSK3β, and that its inhibition prolongs survival of mice transplanted with human AML and stimulates NK cytotoxicity via increased adhesion of NK cells to their targets.

## Graphical abstract

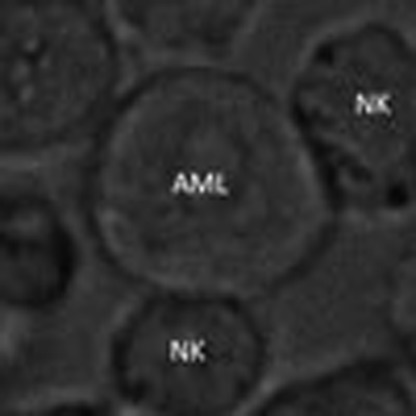

Natural killer (NK) cells are lymphocytes that kill malignant or virally infected cells without antigen-specific receptor recognition. Due to their high activity in specifically killing cancer cells, efforts have been made to utilize *ex vivo* expanded donor NK cells for cancer therapy. While NK cells have been used to target numerous malignancies, haematologic malignancies including acute myeloid leukaemia (AML) have shown particular potential for this approach^1^. In fact, the use of haploidentical NK cells has been found to be successful for treating at least some AML patients^2^,^3^,^4^.

NK cells lead to specific killing of cancer cells due to the expression of a variety of activating (for example, NKG2D) and inhibitory receptors (for example, killer inhibitory receptors) on their surface. These receptors interact with specific ligands on target cells and the balance of these activating and inhibitory signals determines whether cell killing occurs. Cancer cells commonly upregulate ligands for NK cell activating receptors such as MICA/B and downregulate ligands for inhibitory receptors such as HLA class-1 (ref. 5). This HLA downregulation avoids T-cell detection making many cancer cells paradoxically sensitive to NK cell killing.

NK cells exert anti-tumour effects through both direct cytotoxic effects and cytokine production. NK cell-mediated killing of malignant cells depends on several discrete steps that ultimately lead to the polarization and exocytosis of lytic granules towards the target cell^6^. The contact between NK and target cells is the first step and is established through NK cell receptors and adhesion molecules. Engagement of lymphocyte function-associated antigen 1 (LFA-1) by its ligand, intercellular adhesion molecule-1 (ICAM-1), on target cells is one such interaction resulting in the stable adhesion of NK cells to their target cells and is sufficient to induce the polarization of lytic granules in resting NK cells^7^. Another important step is cytokine production by NK cells including interferon-γ (IFN-γ) and tumour necrosis factor-α (TNF-α)^8^. The exact role of these cytokines in NK cell cytotoxic function is not yet fully clear.

NK cells in AML patients are known to exhibit significant defects in cytotoxic activity and to be markedly reduced in number^9^. Recent studies showed that downregulation of activating receptors on NK cells, particularly NKG2D and the natural cytotoxicity receptors NKp46 and NKp30, and defective AML-NK synapse formation are partially responsible for the NK cell dysfunction^10^,^11^,^12^. However, specific signalling alterations leading to these functional changes are not clear.

In an effort to understand the dysregulation of NK cells in AML patients, we found that glycogen synthase kinase beta (GSK3-β) protein levels are upregulated in NK cells from AML patients as compared with normal donors. Importantly for purposes of adoptive cell therapy, NK cells from both AML patients as well as normal donors show a significant enhancement in cytotoxic activity after GSK3 inhibition. GSK3 is a serine threonine protein kinase that plays a central role in a number of key signalling pathways such as Wnt/β-catenin and NFκB, as well as biological processes such as cellular proliferation, inflammation and apoptosis^13^. GSK3 has previously been shown to be a promising target in AML cells as GSK3 inhibitors lead to the growth inhibition and differentiation of leukaemic cells^14^,^15^. Although not much is known about the role of GSK3 in lymphocytes, GSK3 inhibition has been reported to arrest CD8+ T-cell development and promote the survival of T regulatory cells. The inhibition of GSK3 increases interleukin-2 (IL-2) production and lymphocyte proliferation *in vitro*^16^. The functions of GSK3 in NK cells is less clear. NK cells from patients with X-linked lymphoproliferative (XLP) fail to phosphorylate GSK3 following NK r**e**ceptor 2B4 (CD244) stimulation and this lack of phosphorylation after stimulation has been reported to be partially responsible for the NK cell defects observed in these patients^17^. In addition, the drug enzastaurin, which is an activator of GSK3 as well as inhibitor of protein kinase Cβ, was found to suppress NK cell cytotoxic activity^18^. Here we demonstrate that GSK3 inhibition in NK cells results in increased TNF-α production by these cells, which upregulates ICAM-1 on AML cells and results in increased NK-AML conjugate formation resulting in enhanced killing of AML cells.

## Results

### NK cells from AML patients exhibit high levels of GSK3β

**Figure 1:**
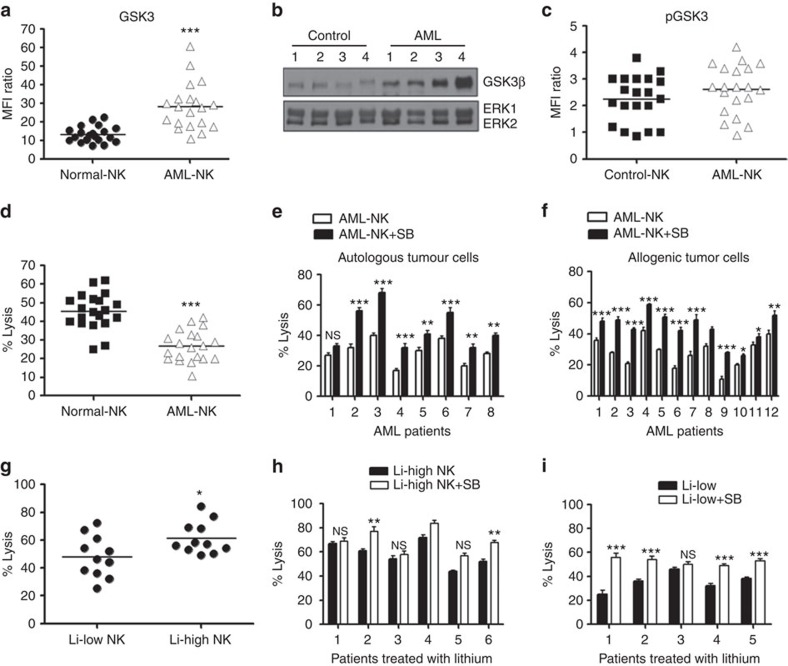
GSK3 plays a role in NK cell activity in AML. (**a**,**b**) NK cells were isolated from AML patients and normal donors (*n*=20 of each). (**a**) GSK3β expression by flow cytometry, Data are represented as mean fluorescence intensity (MFI) ratio. MFI ratio=GSK3β-MFI/Isotype-MFI. Experiment is performed three times. (**b**) GSK3β expression by western blot analysis. An equal amount of protein from NK cells isolated from four different (1–4) normal individuals and AML patients (1–4) was loaded in all wells and the expression of the kinases ERK1 and ERK2 was probed as a control. Experiment is performed two times (**c**) phospho-GSK3β (p-GSK3β) expression was measured by flow cytometry. MFI ratio=MFI p-GSK3β/Isotype. Experiment is performed three times. (**d**) NK cells were isolated from AML patients and normal donors (*n*=20 of each) and their cytotoxic activity was tested against OCI-AML3 cells. Experiment is performed eight times (**e**,**f**) NK cells isolated from AML patients, treated for 16 h with SB or vehicle, and co-cultured with (**e**) autologous AML cells (*n*=8) or (**f**) OCI-AML3 cells (*n*=12). Experiment is performed two times. (**g**) NK cells isolated from lithium treated patients were sorted out into lithium-low (Li-low, <0.6 mM) and lithium-high (Li-high, >0.6 mM) groups based on lithium blood levels. The NK cells were tested for cytotoxic activity against OCI-AML3 cells. (**h**,**i**) The NK cells described in **f** were treated with vehicle or SB for 16 h, co-cultured with OCI-AML3 cells and cytotoxicity was assessed. (unpaired Student's *t*-test ***P<0.001, **P<0.01, NS=not significant *P*>0.05). Experiment is performed two times. Non-expanded NK cells from AML patients, normal donors as well as lithium treated patients were used in this experiment.

NK cells from AML patients are known to exhibit significant functional defects that are implicated in AML development, progression and relapse. Although phenotypic changes in these cells have been described^11^, specific causes for this dysfunction are less clear. As GSK3 protein expression is known to be elevated in cancer cells^19^,^20^,^21^,^22^,^23^,^24^,^25^,^26^, we tested whether GSK3 protein expression is altered in NK cells from AML patients. Interestingly, NK cells from AML patients express higher levels of GSK3β as compared with NK cells from normal donors (Fig. 1a,b, Supplementary Fig. 1). No difference in GSK3β phosphorylation (serine-9) was detected (Fig. 1c). In addition, as previously reported NK cells from AML patients exhibit a reduced ability to kill target AML cells as compared with normal donor NK cells (Fig. 1d).

Next, we tested whether the impaired NK cell function in AML patients could be overcome by GSK3 inhibition. Interestingly after treatment with a specific GSK3 inhibitor SB415286 (SB), NK cells showed enhanced efficacy in killing their own AML cells (Fig. 1e). These AML patient-derived NK cells also showed an enhanced ability to kill allogeneic AML cells after GSK3 inhibition (Fig. 1f). These results suggest that high GSK3 activity may directly contribute to NK cell dysfunction in AML and that GSK3 inhibition may significantly enhance the efficacy of NK cell therapy for AML patients.

To begin to explore whether regulating GSK3 activity *in vivo* can impact NK cell activity, we took advantage of the fact that lithium is currently an Food Drug and Administration-approved GSK3 inhibitor that is used in patients with bipolar disease. It has previously been reported that lithium levels slightly lower than 1 mM are necessary *in vivo* to significantly inhibit GSK3 (ref. 27). We tested the activity of NK cells isolated from patients taking lithium. Interestingly, NK cells from patients with high circulating levels (>0.6 mM) of lithium sufficient to impair GSK3 demonstrate a statistically significant increase in cytotoxic activity as compared with NK cells from individuals with low lithium levels (<0.6 mM) that are not known to lead to significant GSK3 inhibition (Fig. 1g). Further when these NK cells were treated with additional GSK3 inhibitor *ex vivo*, a much more dramatic increase in cytotoxic activity was observed in the lithium-low group suggesting that their GSK3 activity was not optimally inhibited *in vivo* (4/5 patients in lithium-low group versus 2/6 in lithium-high group demonstrated statistically significant enhanced activity, Fig. 1h,i). These results with lithium-treated patients suggest that treatment of AML patients with GSK3 inhibitors may have therapeutic benefit by enhancing NK cell activity. It should be noted, however, that these patients likely also exhibit differences in genetic and epigenetic factors that could also influence NK cell activity. The elevated GSK3 protein in NK cells from AML patients in conjunction with our studies demonstrating that GSK3 inhibition increases NK cell cytotoxic activity towards AML cells, defines a new mechanism for NK cell dysfunction in AML.

### GSK3 inhibition enhances NK cell cytotoxicity towards AML

**Figure 2:**
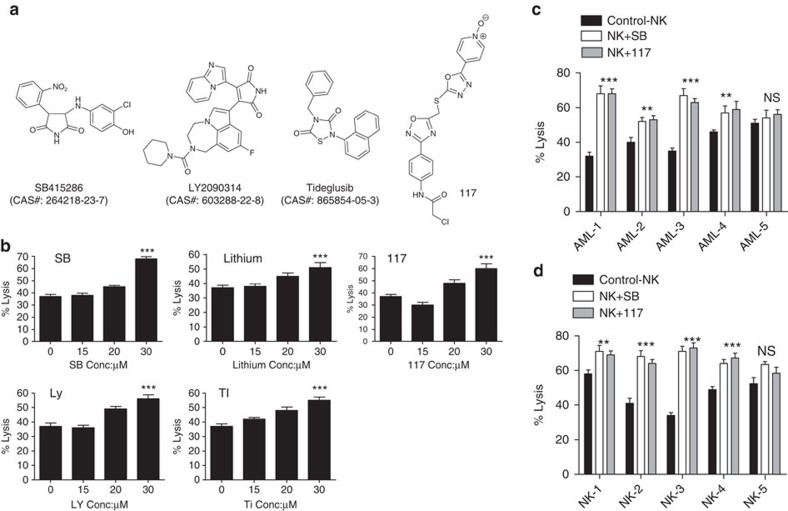
Structurally distinct GSK3 inhibitors enhance NK cell activity against AML cells. (**a**) Chemical structures of GSK3 inhibitors SB415286, LY-2090314, Tideglusib and compound 117. (**b**) Expanded NK cells isolated from normal donors were pre-treated with vehicle or the indicated concentrations of the GSK3 inhibitors for 16 h. The NK cells were then incubated with OCI-AML3 cells and the calcein-AM assay was performed after 4 h. (**c**) Expanded NK cells were pre-treated with vehicle, SB, or compound 117 for 16 h, incubated with primary AML patient leukaemic cells and the calcein-AM assay was performed. (**d**) NK cells from three normal donors were expanded, pre-treated with vehicle, compound 117 or SB for 16 h. The cells were incubated for 4 h with OCI-AML3 cells and lysis was measured using the calcein-AM assay. (unpaired Student's *t*-test ***P<0.001, **P<0.01, NS=not significant *P*>0.05). Conc.=concentration. Experiment is performed eight times.

While the use of donor NK cells for cancer is an area of intense interest, these NK cells are limited by sub-optimal cytotoxic activity that limits their clinical potential. Due to the observation that targeting GSK3 enhances the ability of NK cells to kill AML cells, we explored the potential of using GSK3 inhibition as an NK cell hyperactivation strategy for adoptive cell therapy. Pretreatment of NK cells with several structurally distinct GSK3 inhibitors resulted in enhanced killing of AML cells. Importantly, this enhanced NK cell cytotoxic activity only involves a short *ex vivo* exposure (16 h) to GSK3 inhibitors. Therefore, this strategy does not require a patient to be exposed to the high doses of GSK3 inhibitors that are necessary for potent kinase inhibition, as well as NK cell hyperactivation. Five structurally distinct GSK3 inhibitors (Fig. 2a) led to dose-dependent enhancement of NK cell activity against an AML cell line, OCI-AML3 and primary AML patient samples (Fig. 2b and Supplementary Fig. 2). To appreciate the ability of GSK3 inhibition to enhance NK cell-mediated killing in AML, a panel of primary AML cells from different patients was tested. The GSK3-inhibited NK cells demonstrated a significant enhancement in cytotoxic activity in four of five samples tested (Fig. 2c).

As NK cells are highly heterogeneous even among normal donors, we also tested the impact of GSK3 inhibition using a panel of different NK cells from normal donors. All donor NK cells tested showed increased cytotoxicity after GSK3 inhibition towards AML cells as compared with vehicle-treated cells (Fig. 2d). These results indicate that inhibition of GSK3 can significantly augment normal donor NK cell cytotoxicity activity against AML cells.

### Genetic evidence for the role of GSK3 in NK cell cytotoxicity

**Figure 3:**
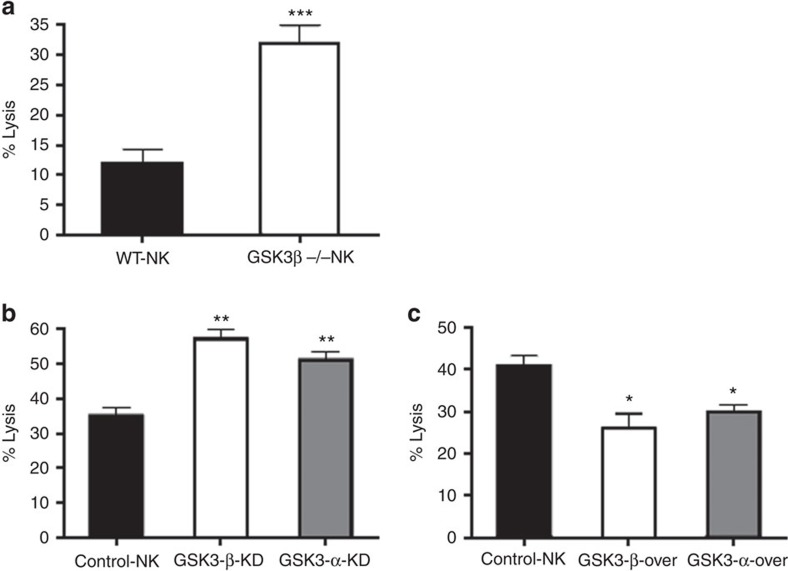
Genetic evidence of the role of GSK3 in NK cell activity against AML cells. (**a**) NK cells were isolated from three GSK3β deficient mice or three wild-type controls were incubated with WEHI-231 mouse cancer cells and target cell lysis was measured using the calcein-AM assay. Experiment is performed four times. (**b**,**c**) NK cells lentivirally transduced with empty vector, GSK3β shRNA (GSK3-β-KD), GSK3α shRNA (GSK3-α-KD), GSK3β (GSK3-β-over) or GSK3α (GSK3-α-over) were incubated with OCI-AML3 cells and target cell lysis was measured using the calcein-AM assay. (unpaired Student's *t*-test ***P<0.001, **P<0.01, *P<0.05, NS=not significant *P*>0.05. Experiments is performed three times.

To further assess the importance of GSK3 in NK cell-mediated killing of AML cells, we utilized genetic approaches. As the loss of GSK3β is embryonic lethal, we crossed floxed-GSK3β mice to Vav/Cre mice, which led to a loss of GSK3β expression in hematopoietic cells (Supplementary Fig. 3). NK cells derived from these mice showed a significantly increased cytotoxic activity against the mouse cancer cell line, WEHI-231 as compared with wild-type age matched mice (Fig. 3a). NK cells lacking GSK3β exhibited 30–40% tumour cell killing compared with 10–15% killing by wild-type NK cells. Of note all reported small molecule GSK3 inhibitors impact both GSK3α and GSK3β making it difficult to discern the importance of specific isoforms in NK cell activity. These results using GSK3β-deficient mice demonstrate that GSK3β plays an important role in NK cell activity.

Besides utilizing a genetic approach in mice, we further assessed the impact of GSK3 in human NK cells through overexpression or knockdown of GSK3 (Supplementary Fig. 4). As shown in Fig. 3b, knockdown of either GSK3α or GSK3β enhanced NK cell-mediated killing of OCI-AML3 cells. Similarly, overexpression of both isoforms decreased NK activity (Fig. 3c). The lower level of NK cell activity in these genetic studies as compared with those using small molecule inhibitors suggest that the simultaneous inhibition of both isoforms is necessary for maximal effect.

### GSK3 inhibition enhances TNF-α production by NK cells

**Figure 4:**
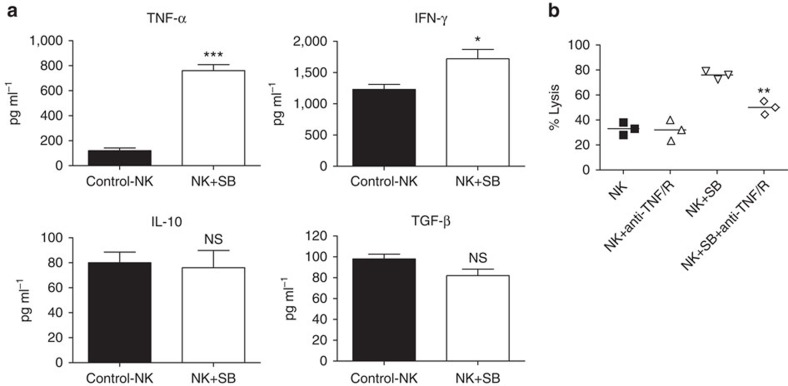
GSK3 inhibition leads to NK cell hyperactivation partially in a TNF-α-dependent fashion. (**a**) Expanded NK cells were incubated with vehicle or SB for 16 h, co-cultured with OCI-AML3 cells for 2 h and then the indicated cytokine levels were measured in the conditioned medium by ELISA. (**b**) Expanded NK cells were treated with vehicle or SB for 16 h, anti-TNF-receptor antibody (anti-TNF-R) and anti-TNF antibody (anti-TNF) were added to the indicated wells, the cells were co-cultured with OCI-AML3 cells and target cell lysis was measured using the calcein-AM assay. (unpaired Student's *t*-test ***P<0.001, **P<0.01, *P<0.05, NS=not significant *P*>0.05). Experiment is performed three times.

To explore mechanisms through which GSK3 inhibition enhances NK cell activity, we assessed NK cell cytokine production. NK cells produce a wide variety of cytokines and chemokines including IFNγ, TNF-α and IL-10 (refs 28, 29). We identified that GSK3 inhibitor treatment of NK cells with SB leads to a marked induction in TNF-α levels (>sixfold) and a modest increase in IFNγ (Fig. 4a). In contrast, there was no change in transforming growth factor-β (TGF-β) or IL-10 levels (Fig. 4a). TNF neutralization markedly impairs the cytotoxic activity of the GSK3-inhibited NK cells (Fig. 4b). Importantly, TNF-α neutralization did not impact the activity of control NK cells suggesting that TNF-α plays an important role specifically in GSK3 inhibitor-mediated NK cell hyperactivation. As TNF-α itself is known to lead to cancer cell death, we tested whether the levels of TNF-α produced could lead to direct leukaemia cell death. TNF-α treatment did not lead to evidence of AML cell death at the doses and time points tested demonstrating that the cell killing is mediated by the NK cells (Supplementary Fig. 5).

**Figure 5:**
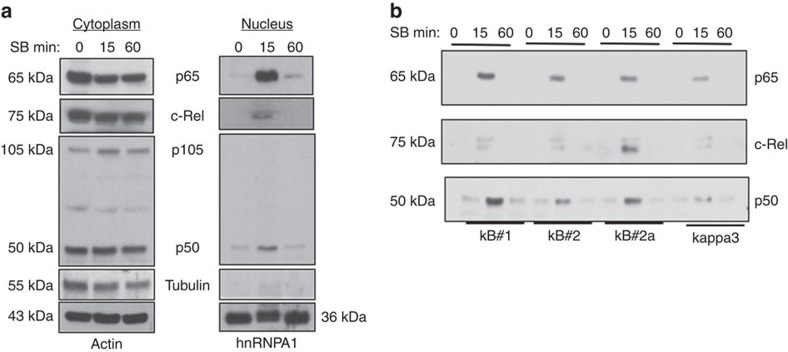
GSK3 inhibition leads to NF-kB activation and its binding to the TNF promoter in human NK cells. (**a**) Human NK cells were treated with the GSK3 inhibitor SB for the indicated times, cytoplasmic and nuclear lysates were prepared, and analysed by Western blotting using indicated antibodies. Actin and hnRNPA1 were used as internal loading controls for cytoplasmic and nuclear extracts. Tubulin was used as a control to examine the purity of nuclear extracts. Experimental control in which NK cells were treated with DMSO, the vehicle used to dissolve SB is shown in Supplementary Fig. 6. (**b**) Expanded NK cells were treated with SB for the indicated times. Nuclear extracts were prepared and analysed by Oligonucleotide pull-down assay to examine NF-kB binding to the TNF promoter. Data presented are representative of two independent studies. Experiment is performed three times.

Since TNF-α plays a major role in GSK3 inhibitor-mediated NK cell activity, we further explored mechanisms leading to its induction. As TNF-α is an NF-κB target gene, we checked the impact of GSK3 inhibition on NF-κB signalling in NK cells. We observed an increase in nuclear translocation of the NF-κB subunits p65, c-Rel and p50 in SB treated NK cells (Fig. 5a), whereas dimethylsulphoxide (DMSO)-treated NK cells did not show any NF-κB activation at the time points tested (Supplementary Fig. 6). To demonstrate that the NF-κB activation in NK cells is indeed responsible for the observed TNF induction, we performed a nucleotide pull-down assay using four known NF-κB-binding regions in the TNF-α promoter^30^. We found that p65; p50 and c-Rel binding to the TNF-α promoter were enhanced following GSK3 inhibition (Fig. 5b). Interestingly, we observed enrichment of two distinct NF-κB complexes; (1) a p65/p50 complex binding to the κB1 region and (2) a complex containing p65, p50 and c-Rel binding to the κB2a region. Inducible c-Rel occupancy was found to be unique to the κB2a region. The varying binding intensity of the NF-κB subunits to the four regions in the TNF promoter shows that the binding is inducible and specific to the cognate DNA sequences.

### Increased conjugate formation by GSK3-inhibited NK cells

**Figure 6:**
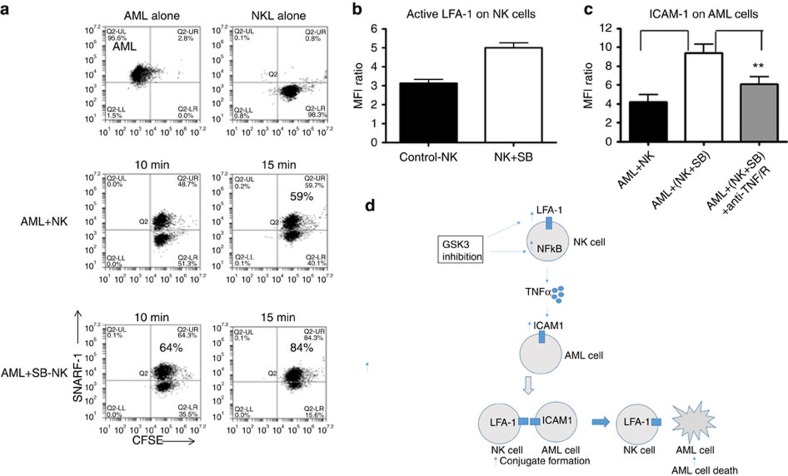
GSK3 inhibition modulates surface receptors on human NK cells and target cells after co-culture and leads to enhanced conjugate formation. (**a**) Expanded human NK cells labelled with CFSE (treated with either DMSO or SB overnight) were co-incubated with OCI-AML3 cells labelled with SNARF-1 dye. The cells were co-cultured and conjugate formation was analysed using flow cytometry at the indicated times. As a control, isolated populations of NK cells and OCI-AML3 cells were analysed. NK:AML ratio=2:1. This is a representative study of 3 independent experiments. (**b**) Expanded NK cells were treated with SB or vehicle for 16 h and the surface expression of active LFA-1 was assessed by flow cytometry. Data shown are the mean fluorescence intensity (MFI). MFI ratio=MFI LFA-1/MFI Isotype. (**c**) Expanded NK cells were treated with SB or DMSO for 16 h and then were co-cultured with OCI-AML3 cells with or without TNF neutralization (TNF-R and TNF antibodies). Data shown are the mean fluorescence intensity (MFI). MFI ratio=MFI ICAM-1/MFI Isotype on gated OC-AML3 cells. (unpaired Student's *t*-test ***P<0.001, **P<0.01). (**d**) Model of GSK3 regulation of NK cell activity against AML. Experiment is performed four times.

NK cell killing requires conjugate formation between NK and target AML cells. This interaction forms NK cell immunological synapses that lead to the activation of a cascade of intracellular signals leading to target cell lysis^31^. To determine the effect of GSK3 inhibitors on NK cell conjugate formation, we co-incubated separately labelled AML and NK cells. Interestingly, GSK3 inhibitor-treated NK cells led to a dramatic increase in conjugate formation with AML cells as compared with vehicle control NK cells (Fig. 6a).

As conjugate formation involves the interaction of cell surface molecules on NK and target cells, we measured the expression of a variety of NK cell surface receptors as well as granzyme/perforin which directly mediates killing after an overnight incubation of NK cells with GSK3 inhibitor. We observed a modest increase in perforin and granzyme expression in GSK3-inhibited NK cells as compared with control cells (Supplementary Fig. 7). While we did not identify significant changes in the expression of most cell surface receptors tested, LFA-1 (active form) and ICAM-1 were induced on the NK cells and AML cells, respectively (Fig. 6b,c and Supplementary Fig. 6). LFA-1 and ICAM-1 expression on NK cells and AML cells, respectively, is reported to play an important role in AML-NK conjugate formation.

To delineate the role of TNF-α in this conjugate formation, we checked ICAM-1 and LFA-1 expression on AML and NK cells, respectively, after TNF neutralization. Neutralization of TNF-α decreased ICAM expression in AML cells as well as impaired NK cell killing (Figs 4b and 6c). Although LFA-1 is not regulated by TNF-α, it has been previously shown that recycling of other integrins and their expression on the cell surface is regulated by GSK3 (ref. 32). This study supports the model that GSK3 inhibition activates NF-κB, which leads to TNF-α production, induction of cell surface receptors, enhanced conjugate formation and subsequent AML cell killing (Fig. 6d).

### GSK3-inhibited NK cells show increased cytotoxicity *in vivo*

**Figure 7:**
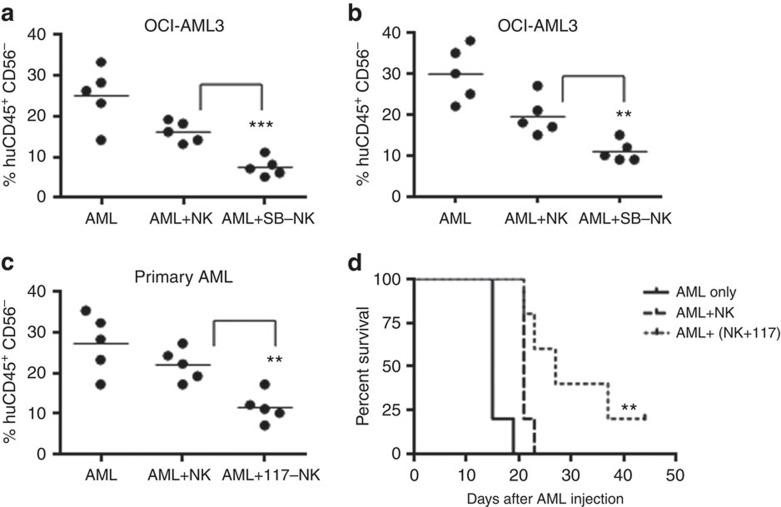
GSK3-inhibited NK cells show high activity in AML mouse model systems. (**a**,**b**) NSG mice were injected with OCI-AML3 cells followed by weekly injections of vehicle, expanded NK cells, or expanded NK cells that were pre-treated with SB for 16 h. The per cent human AML cells (CD56^−^ CD45^+^) were detected by flow cytometry from (**a**) spleen and (**b**) bone marrow after 6 weeks (**c**) NSG mice were injected with primary AML cells followed by weekly injections of vehicle, expanded NK cells, or expanded NK cells that were pre-treated with compound 117 for 16 h. The per cent human AML cells (CD56^−^ CD45^+^) were detected by flow cytometry from the bone marrow after 5 weeks. (unpaired Student's *t*-test ***P<0.001, **P<0.01). (**d**) NSG mice were injected with primary human AML cells followed by four (bi-weekly) injections of vehicle, expanded NK cells or expanded NK cells pre-treated with compound 117 for 16 h. Kaplan–Meier survival curves of mice are shown. *χ*^2^ log-rank test was performed for statistical significance. **P<0.01 for AML+NK versus AML+ (NK+117) groups. Experiments is performed two times. In all mice experiments, five mice per group were used.

To assess the potential of GSK3-inhibited NK cells for AML therapy, we utilized models of human AML in immunodeficient mice using OCI cells (Fig. 7a,b) and a primary AML patient sample derived from a patient with relapsed, refractory disease (Fig. 7c,d). In both models human AML cells were injected into the tail vein of mice and circulating AML that mimics the human disease was established. We tested the efficacy of NK cells on disease burden by sacrificing mice when the non-NK cell injected mice became moribund as well as survival. Similar to the *in vitro* studies, NK cells treated with GSK3 inhibitor (SB and 117) were more effective in killing leukaemic cells as evidenced by a significant reduction in leukaemic cell burden in the mouse bone marrow and spleen (Fig. 7a–c). We also observed a significant improvement in mouse survival when utilizing 117 pre-treated NK cells as compared with control-treated NK cells. (Fig. 7d). Importantly, similar to the *in vitro* studies, the GSK3 inhibitor-treated cells performed significantly better *in vivo* than the vehicle-treated and *ex vivo* expanded NK cells, which have previously been tested in clinical trials for haematologic and non-haematologic malignancies.

## Discussion

NK cells have long been known to play a major role in immune monitoring to prevent the development and progression of cancer^33^. Unfortunately, NK cells in AML patients are known to exhibit significant defects in number and cytotoxic activity that are implicated in AML development, progression and relapse^9^,^12^. Although phenotypic changes in these cells have been described, specific molecular causes for this dysfunction are less clear^11^. It has, for example, been reported that tumour microenvironment factors such as PGE2, TGF-β1 and IL-10 may play a role (reviewed in ref. 34). Further alterations in NK cell surface receptor expression have been reported such as a reduction in NKG2D expression as well as changes in conjugate formation.

Here we show that GSK3 plays an important role in NK cell cytotoxic activity against AML. Both genetic and pharmacologic manipulation of GSK3 was found to impact the ability of NK cells to kill AML cells. We have uncovered mechanistic insights into how GSK3 modulates the ability of NK cells to kill cancer cells (see model in Fig. 6). GSK3 inhibition leads to a rapid and transient activation of NF-κB in NK cells. This activated NF-κB then binds to the TNF-α promoter and strongly induces TNF-α that is secreted from the NK cells and upregulates ICAM-1 on target AML cells. ICAM-1, a TNF-α target gene, is a cell surface protein that is involved in interactions between NK cells and target cancer cells that are required for killing^8^,^35^,^36^,^37^,^38^,^39^,^40^. In addition to ICAM-1 upregulation on the surface of target AML cells, ICAM-1′s interacting partner on NK cells, LFA-1, is induced on the surface of NK cells by GSK3 inhibition. Consistent with our findings, the expression of integrins on the cell surface has previously been shown to be modulated by GSK3 (ref. 31). The increased NK-AML conjugate formation resulting from increased cell adhesion molecules is one of the major factors known to account for NK cytotoxicity. Besides the ICAM-1/LFA-1 proteins, it is likely that other cell surface proteins are also modulated by GSK3 in both NK cells and target cells that also impact NK/target cell conjugate formation and subsequent AML cell killing. The increased killing effect noticed after GSK3 inhibition is not due to direct TNF-α-induced killing as our data support previous reports that AML cells are highly resistant to TNF-α-induced apoptosis^41^.

In addition to showing that high levels of GSK3 can impair NK cell function, we observed elevated GSK3β expression in NK cells from AML patients that are functionally defective. These studies suggest that this elevated GSK3β protein in NK cells from AML patients may contribute to NK cell dysfunction that has long been known to occur in AML. Interestingly, it has previously been reported that GSK3 protein is upregulated in a wide variety of cancer cells including leukaemia and that GSK3 may play a role in disease progression^15^,^22^,^26^. Our work here demonstrates that this dysregulation of GSK3 protein expression is not limited to cancer cells. GSK3 inhibition in AML cells is known to enhance AML differentiation and growth inhibition^14^,^15^,^42^. In fact GSK3 inhibitors are currently being tested clinically for cancer due to their direct anti-cancer activities (NCT01632306) (NCT01287520) (NCT01214603). In contrast to these previous studies, our study demonstrates that GSK3 inhibition not only impacts AML cells directly, but also hyperactivates NK cells and leads to AML cell killing. Although our study has focused on AML, it is likely that GSK3 is an important mediator of NK cell activity in other disease contexts. Future studies will investigate the importance of GSK3 in the function of NK cells from other cancer patients beyond AML.

Besides demonstrating the role of GSK3 in NK cell function, another important finding from this work relates to revealing how GSK3 regulates NF-kB in NK cells. Studies investigating the role of GSK3 in modulating NF-κB signalling pathways have led to a variety of observations that are dependent on experimental conditions and cell types tested. For example, in hepatocytes the absence of GSK3 inhibits NF-κB^43^,^44^, while the GSK3 inhibitor lithium activates NF-κB in human intestinal epithelial cells^45^ and TNF-α production by monocytes and macrophages^46^. The rapid activation of NF-κB in NK cells on GSK3 inhibition in minutes suggests a direct role of GSK3 in suppressing NF-κB activation. This window of time is not sufficient to permit secondary effects such as new protein synthesis. Therefore, it will be of interest to examine mechanisms through which GSK3 may directly suppress NFκB activity in NK cells. For example, GSK3 may act as a cytoplasmic sequester of NF-κB. Consistent with a previous study^30^, strong binding of NF-κB to κB#1 and κB#2a sites on the TNF-α promoter was detected. Interestingly, a differential recruitment of NF-κB dimers was observed in the regions where κB#1 showed increased affinity for the p65 complex and κB#2a showed increased affinity for the c-Rel complex. The physiological relevance of the occupancy of these distinct NF-κB complexes in adjacent promoter regions of the same gene, that is, TNF-α, may suggest a mechanism for tight regulation of TNF-α production in cells.

Our study has also established a new therapeutic strategy for AML in addition to revealing the role of GSK3 in NK cell function. Inhibition of GSK3 by small molecules leads to the hyperactivation of both normal donor as well as leukaemia patient NK cells. These hyperactivated cells exhibit a significantly improved ability to kill AML cells in both cell and animal systems. Although adoptive NK cell therapy for AML has shown promise^2^,^47^, previous studies suggest that NK cells with higher levels of cytotoxic activity would be highly desirable^3^. Due to the fact that the hyperactivation only requires a short *ex vivo* treatment with a GSK3 inhibitor, the translation of this strategy to the clinic should be rapid. As the GSK3 inhibitor does not need to be given directly to the patient, high doses of drug can be used that are not able to be safely administered to patients. Although an *ex vivo* approach has significant benefits, our study assessing individuals on lithium therapy suggests that direct administration of a GSK3 inhibitor can significantly modulate NK cell activity *in vivo* at least in a non-cancer patient. It has also been previously reported that inhibiting GSK3 can lead to the upregulation of NK cell ligands on target multiple myeloma cells providing another possible benefit of direct GSK3 inhibitor administration^48^.

In conclusion, our results suggest a role of GSK3 in the dysfunction of NK cells in AML. In addition, our results highlight a novel therapeutic strategy to hyperactivate NK cells. It is hoped that this approach will lead to improved adoptive NK cell therapies for patients with AML as well as potentially other malignancies.

## Methods

### Patient samples

Normal donor and AML patient blood samples were obtained from the Hematopoietic Stem Cell Core Facility at Case Western Reserve University. Only discarded human blood samples were used in this study and informed consent documents for this study were approved by the the University Hosptials Case Medical Center Institutional Review Board.

### NK cell isolation and expansion

Peripheral blood mononuclear cells (PBMC) were first isolated from peripheral blood using Ficoll-Paque Plus (GE HealthCare, Piscataway, NJ, USA). PBMC were co-cultured with irradiated (100 cGy) K562 artificial Antigen Presenting Cells (aAPCs)^49^ at a ratio of 1:2 (PBMC:aAPC). Cultures were refreshed with half-volume media that changes every 2 to 3 days, and re-stimulated with aAPCs at a ratio of 1:1 every 7 days. NK cell expansion media is a complete medium consisting of RPMI 1640 supplemented with 10% human serum, 10 U ml^−1^ penicillin, 100 mg ml^−1^ streptomycin and 50 IU ml^−1^ IL-2 (Gold Biotechnology, St Louis, MO, USA). Human NK cells were negatively selected using either RosetteSep (Stem Cell Technologies, Vancouver, Canada) containing antibodies to CD3, CD36, CD4, CD66b, CD19 and glycophorin A or the Human NK isolation kit (Miltenyi Biotec, Inc., San Diego, CA, USA) before experiments. The purity of NK cells (>95% CD3^−^, CD16^+^, and CD56^+^) was confirmed by flow cytometry using CD3 (Cat No-300305, 1:100) CD16 (Cat No-302007, 1:100) and CD56 (Cat No-362503, 1:100) antibodies (Biolegend, San Diego, CA, USA). For mouse NK cell isolation from spleen, the Mouse NK isolation kit (Miltenyi Biotec, Inc., San Diego, CA, USA) was used.

### Antibodies

The following antibodies were used: GSK3β (9315, 1:1,000) (Cell Signaling), CD34 (340667, 1:100), ICAM-1 (559771, 1:100) (BD Biosciences, San Jose, CA, USA) and anti CD11a antibody (52895, 1:100) (Abcam, Cambridge, UK). For western blot analysis p65 (sc-372, 1:2,000), c-Rel (sc-71, 1:1,000), p50 (sc-7178, 1:1,000), p105 (sc-7178, 1:1,000), actin (sc-69879, 1:10,000), hnRNPA1 (sc-32301, 1:2,000), tubulin (sc-5546, 1:1,000) histone H3 (sc-10809, 1:1,000), ERK1 and ERK2 antibodies (sc- (Santa Cruz Biotechnology, Inc., Santa Cruz, CA, USA).

### Ethics

The Case Western Reserve University Animal Research Committee approved all of the animal protocols used in this study.

### Mice

GSK3β floxed C57bl mice, were a kind gift from Dr Woodgett (Lunenfeld-Tanenbaum Research Institute, Toronto, Ontario)^50^. These mice were crossed to Vav-Cre (The Jackson Laboratory, Maine, USA) and genotyped as previously described^51^. Littermate controls were used for comparisons. Vav-Cre GSK3β mice (C57bl, female 6–7 weeks old) were used for experiments. All mice experiments were done according to Institutional Animal Care and Use Committees (IACUC) guide lines. Nod-Scid IL-2Rg−/− (NSG) mice were purchased from The Jackson Laboratory (Bar Harbor, ME, USA). OCI-AML3 cells or a primary AML sample (3 × 10^5^ cells) were injected into the femur (*n*=5 mice per group). After 1 week of AML cell transplantation, 10^7^ NK cells (control or GSK3 inhibited) were intravenously injected together with IL-2 (3 μg per mouse) one time a week either for 4 (OCI-AML3 cells) or 5 weeks (primary AML cells). Human CD45^+^ CD56^−^ AML cells were assessed using flow cytometry in the bone marrow and spleen on week 5 or 6.

### GSK3 inhibitors

SB (Tocris Biosciences (Minneapolis, MN, USA), lithium chloride (Li) sigma (St Louis, MO, USA), tideglusib (Ti) and LY-2090314 (LY) (Selleckchem.com, Munich, Germany) and 117 were used. NK cells were pre-treated with GSK3 inhibitors overnight (16 h), washed two times with media and used for experiments. Compound 117 is a novel GSK3 inhibitor that we identified in our lab. Confirmation of its ability to inhibit GSK3 using a kinase assay in a similar capacity to SB, a widely used GSK3 inhibitor, can be seen in Supplementary Fig. 8. The ADP-Glo kinase assay was performed according to manufacturer's instructions (Promega, WI, USA).

### Synthetic procedures of the final compound 117 and its intermediates

Compound A: To a solution of 4-cyanobenzonitrile (1.18 g, 10 mmol) in ethanol (20 ml), 8-hydroxyquinoline (8 mg, 0.05 mmol) was added. Hydroxylamine hydrochloride (1.56 g, 23mmol) and sodium carbonate (1.79 g, 17 mmol), each dissolved in water (10 ml), were sequentially added over a period of 20 min, then the reaction mixture was heated to reflux for 2 h. After cooling the mixture, solvent was removed *in vacuo*, and silica gel chromatography of the residue (Hexanes/EtOAc/MeOH: 10:10:1) gave intermediate A (grey solid, 0.9 g, 60%). 1H NMR (500 MHz, DMSO-d_6_) δ 9.18 (s, 1H), 7.33 (d, *J*=8.5 Hz, 2H), 6.51 d, *J*=8.5 Hz, 2H), 5.50 (s, 2H), 5.24 (s, 2H); ^13^C NMR (125 MHz, DMSO-d_6_) δ 151.1, 149.3, 126.1, 120.4, 112.9; Liquid Chromatography-Mass Spectrometry (LC-MS) (*m/z*): [M]^+^ calcd (for C_7_H_10_N_3_O) 152, found 152. These data are consistent with the literature data for the same compound^52^.

Compound B: To a solution of A (0.5 g, 3.3 mmol) in acetone (20 ml), chloroacetyl chloride (1.11 g, 10 mmol) was added and the mixture was stirred at room temperature for 2 h. The acetone was evaporated and the residue was washed with sodium bicarbonate solution (10 ml) and water (10 ml) to give the crude off-white solid of compound B, which was dried and used directly for the next step (0.4 g, 40%). ^1^H NMR (500 MHz, DMSO-d_6_) δ 10.64 (s, 1H), 7.69 (s, 3H), 6.92 (s, 2H), 4.54 (s, 1H), 4.30 (s, 1H); ^13^C NMR (125 MHz, DMSO-d_6_) δ 166.1, 165.4, 157.6, 141.1, 128.0, 126.6, 119.3, 44.0, 41.3; LC-MS (*m/z*): [M]^+^ calcd (for C_11_H_12_Cl_2_N_3_O_3_) 304, found 304. Compound C: compound B (0.61 g, 2 mmol) was suspended in *o*-xylene (20 ml) and refluxed for 2 h. After cooling, the mixture was directly loaded on to silica gel column; elution with hexane/EtOAc (3:2) gave an off-white compound C (0.4 g, 70%). ^1^H NMR (500 MHz, DMSO-d_6_) δ 10.62 (s, 1H), 7.99 (d, *J*=8.5 Hz, 2H), 7.80 (d, *J*=8.5 Hz, 2H), 5.17 (s, 2H), 4.30 (s, 2H); ^13^C NMR (125 MHz, DMSO-d_6_) δ 175.7, 167.9, 165.4, 141.9, 128.4, 121.0, 119.9, 43.9, 34.1 LC-MS (*m/z*): [M]^−^ calcd (for C_11_H_8_Cl_2_N_3_O_2_) 284, found 284. Compound D: a mixture of isonicotinamide (0.77 g, 5 mmol), KOH (0.28 g, 5 mmol) and carbon disulfide (4 ml) in ethanol (50 ml) was refluxed for 12 h. The solution was then concentrated, cooled to ∼4 °C and acidified with diluted HCl. The solid precipitated out was filtered, washed with ethanol, and dried to give a light yellow solid D (0.52 g, 53%), which was used directly for the next step. Compound D contains ∼52% of precursor as shown in the NMR and LC-MS (see the supporting information for NMR and LC-MS data). ^1^H NMR (500 MHz, DMSO-d_6_) δ 10.55 (s, 1H), 8.35–8.25 (m, 2H), 7.85 (d, *J*=6.5 Hz, 1H), 7.72 (d, *J*=6.5 Hz, 1H); ^13^C NMR (125 MHz, DMSO-d_6_) δ 162.8, 139.9, 129.1, 125.4, 122.7; LC-MS (*m/z*): [M]^+^ calcd (for C_7_H_6_N_3_O_2_S) 196, found 196 and [M]^−^ calcd (for C_7_H_4_N_3_O_2_S) 194, found 194. The thione tautomer of compound D was synthesized and reported in the literature^53^.

Compound 117: a mixture of D (20 mg, 0.0492, mmol based on an estimated purity of ∼48%), KOH (28 mg, 0.5 mmol) and C (29 mg, 0.1 mmol) in DMSO (2 ml) was stirred at room temperature for 1 h. EtOAc (10 ml) was added to the reaction mixture, and the solution was washed with water (5 ml two times). The EtOAc extract solution was evaporated and the residue was subjected to silica gel chromatography (Hexanes:EtOAc:MeOH, 10:10:1) to give 117 (light brown solid 25 mg, quantitative yield).Compound 117 characterization data: melting point: 118–119 °C; TLC (Hexanes:EtOAc:MeOH, 2:2:1 v/v/v): *R*_f_=0.31; ^1^H NMR (500 MHz, DMSO-d_6_) δ 10.60 (s, 1H, amide H), 8.37 (d, J=7.25 Hz, 2H, CH), 7.95 (d, *J*=8.5 Hz, 2H, CH), 7.90 (d, *J*=7.25 Hz, 2H, CH), 7.77 (d, *J*=8.5 Hz, 2H, CH), 5.02 (s, 2H, C-CH2-S), 4.29 (s, *J*=1.7 Hz, 2H, C(O)-CH2-Cl). ^13^C NMR (125 MHz, DMSO-d_6_) δ 175.6, 167.0, 164.6, 162.9, 162.4, 140.9, 139.4, 127.5, 123.2, 120.3, 119.1, 117.9, 43.1, 26.2; HRMS (*m/z*): [M]^+^ calcd (for C_18_H_14_ClN_6_O_4_S) 445.0485, found 445.0481.

Synthetic scheme for compound 117 is illustrated in Supplementary Fig. 9. More details of the spectral data of Compound 117 and its intermediates are provided in Supplementary Section.

### Preparation of cytoplasmic and nuclear fractions

Cell fractions were prepared by suspending 5 × 10^6^ cells in 200 μl of buffer A (10 mM HEPES, pH 7.6, 10 mM KCl, 0.1 mM EDTA, 0.1 mM EGTA, 1.0 mM DDT, Protease inhibitors). Cell suspensions were incubated on ice for 15 min and 0.5% NP-40 was added and vortexed for 10 s. The samples were centrifuged for 30 s at 10,000*g* at 4 °C, and the supernatant was isolated and used as cytoplasmic fraction. The remaining pellet was washed with buffer A and extracted with 100 μl of buffer C (20 mM HEPES, pH 7.6, 0.4 mM NaCl, 1.0 mM DDT, 1.0 mM EDTA, 1.0 mM EGTA and Protease inhibitors) for 20 min on ice. The samples were centrifuged for 3 min at 10,000*g* in an Eppendorf microfuge at 4 °C and the supernatant was collected and used as nuclear fraction.

### Western blotting

For western blot analysis, 25–40 μg of protein was resolved using 9% polyacrylamide gels and transferred to nitrocellulose membrane. The blot was blocked in 5% non-fat dry milk for 1 h at room temperature and probed with respective antibodies for 2 h at room temperature or overnight at 4 °C. Blots were visualized using horseradish peroxidase-conjugated secondary antibody probing followed by enhanced chemiluminescence^54^. Images have been cropped for presentation. Full size images are presented in Supplementary Figs 10–12.

### TNF-α promoter-binding assay

Oligonucleotides corresponding to four known NF-κB binding regions in the human TNF promoter (Supplementary Fig. 13) were synthesized (IDT, Iowa, USA)^30^. The forward primers were tagged with biotin to facilitate streptavidin pull down. NK cells (200 × 10^6^) were treated with SB for the indicated time points. Nuclear extracts were prepared and lysates corresponding to 100 μg of protein were used for binding and pull down using Neutravidin beads (Thermo Scientific, Fremont, CA). Pull down was carried out overnight at 4 °C in a rotator, beads were washed with lysis buffer containing 20 mM HEPES, pH 7.6, 1% Triton X-100 and 150 mM NaCl, and analysed by western blotting^55^.

### Cytotoxicity assays

NK cells were pre-treated overnight with GSK3 inhibitors in IL-2-free media. These cells were washed three times with plain media to remove any traces of GSK3 inhibitors from the experimental system to avoid direct effect of GSK3 inhibitors on AML cells. The target cells were either the AML cell line OCI-AML3 (DSMZ, Braunschweig, Germany), WEHI-231 (ATCC, Manassas, VA, USA) or primary AML cells isolated from AML patients. The E:T ratio was adjusted to 5:1 for all assays. Target cells were resuspended in complete medium at a final concentration of 10^6^ ml^−1^ and incubated with 15 μM calcein-AM (Life Technologies, Grand Island, NY, USA) for 30 min at 37 °C with occasional shaking. After two washes in medium, cells were adjusted to 10^5^ ml^−1^. Spontaneous (only target cells in complete medium) and maximum release (target cells lysed using 2% Triton X-100) target cells were used as controls. After 4 h incubation at 37 °C, the plate was centrifuged, 200 μl of each supernatant was harvested and transferred into 96-well plates. Samples were measured using a Spectramax Gemini dual-scanning microplate spectrofluorimeter (Molecular Devices, Sunnyvale, CA, USA; excitation filter: 485±9 nm; band-pass filter: 530±9 nm). Per cent lysis was calculated as described previously^56^.

### ELISA

ELISA assays were performed in 96-well ELISA plates using TNF-α, IFN-γ, IL-10 and TGF-β ELISA kits (eBioscience, San Diego, CA, USA) according to the manufacturer's instructions.

### Lentiviral transductions

293 T cells (ATCC, Manassas, VA, USA) were co-transfected with one of the following plasmids: shGSK3β, shGSK3α, pLKO (Sigma-Aldrich, St Louis, MO, USA), GSK3β and GSK3α (both full-length human sequences were cloned into pLVX-EF1alpha-IRES-mCherry using the EcorR1/Not1 sites; Clontech, Mountain View, CA, USA) GSK3β contains a serine to alanine mutation at position 9 and GSK3α contains a serine to alanine mutation at position 21 to prevent the inactivation of these kinases by inhibitory phosphorylation. The packaging plasmids were pCMVdR8.74 and pMD.G (Addgene, Cambridge, MA, USA) using lipofectamine (Invitrogen, Carlsbad, CA, USA). NK cells were infected with the virus containing supernatant concentrated overnight in Polyethylene glycol (PEG) in the presence of 6 μg ml^−1^ of polybrene. Stable cells were generated by selection with puromycin (1 μg ml^−1^).

### Conjugation assay

NK cells were labelled with CellTracker Green CMFDA (Invitrogen, Carlsbad, CA, USA) and AML cells were labelled with SNARF-1 (Invitrogen, Carlsbad, CA, USA). After labelling, cells were incubated at 37 °C for 30 min and washed two times with media. The labelled NK cells (2 × 10^6^ cells per ml) were mixed with labelled target cells (1 × 10^6^). The effector-target cell mixture was centrifuged at 300 r.p.m. for 5 min at 4 °C and then incubated at 37 °C for the indicated time. After incubation, the cell mixture was resuspended by strong vortexing and fixed by adding ice-cold 0.5% paraformaldehyde. The conjugate formation was analysed by flow cytometry.

### Statistical analysis

Applicable data were analysed using the unpaired Student's *t*-test. For all the experiments, unless otherwise indicated, *N*=3, *P* values were assigned *P<0.05, **P<0.01, ***P<0.001. For mice survival analysis, the log-rank test was performed, **P<0.01.

## Additional information

**How to cite this article:** Parameswaran, R. *et al*. Repression of GSK3 restores NK cell cytotoxicity in AML patients. *Nat. Commun.* 7:11154 doi: 10.1038/ncomms11154 (2016).

## Supplementary Material

Supplementary Information Supplementary Figures 1-13

Supplementary Data 1 Chemical characterization of 117
