# Supplementary material for: Repression of GSK3 restores NK cell cytotoxicity in AML patients
Source: Nat Commun. 2016 Apr 4;7:11154. doi: 10.1038/ncomms11154 (PMC4822012; doi:10.1038/ncomms11154)
Supplement: Supplementary Information — Supplementary Figures 1-13 [file ncomms11154-s1.pdf]

Supplementary figure 1

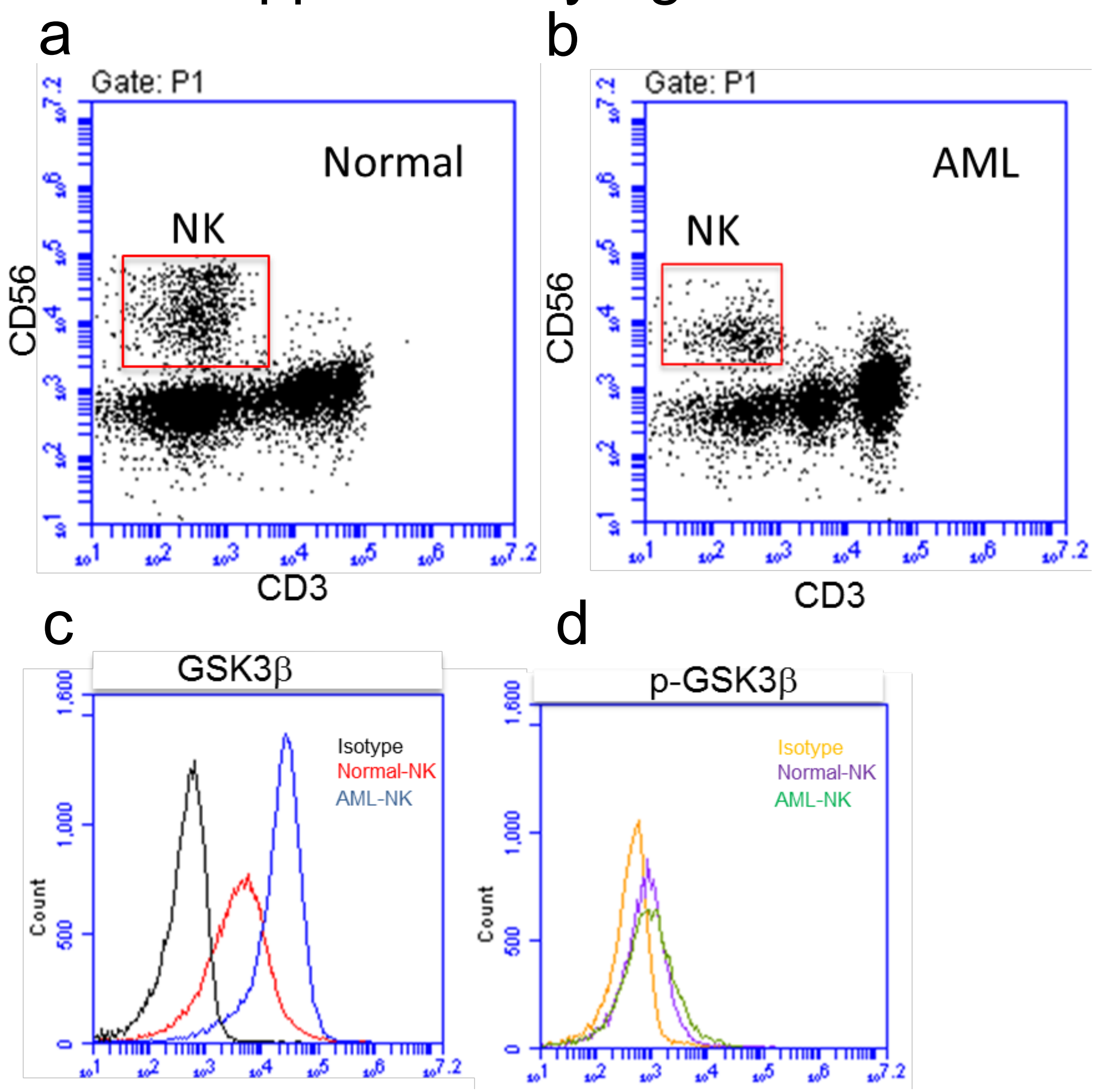

Supplementary Figure 1. NK cells from AML patients exhibit elevated GSK3b protein expression as compared to normal donors. Flow cytometry analysis of mononuclear cells from normal and AML patients. Anti CD56 and CD3 antibodies were used for staining cells. $CD56^+$ $CD3^-$ cells gated as NK were sorted out from normal **(a)** and **(b)** AML patient peripheral blood samples. Sorted NK cells stained with **(c)** anti GSK3β and **(d)** phospho GSK3β antibodies.

Supplementary figure 2

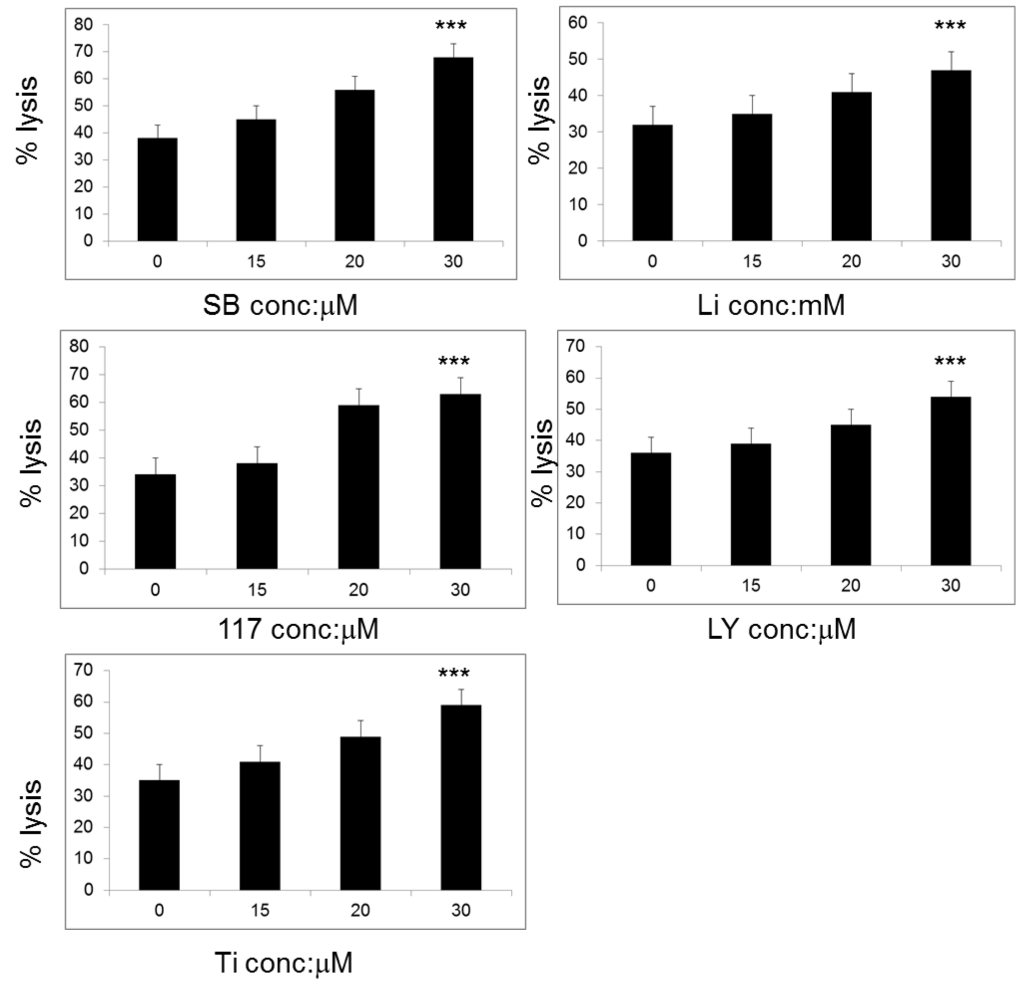

Supplementary Figure 2. Structurally distinct GSK3 inhibitors enhance NK cell activity against primary AML cells. 5 structurally distinct GSK3 inhibitors lead to NK cell hyperactivation against a primary AML sample**.** Expanded NK cells isolated from normal donors were pre-treated with vehicle or the indicated concentrations of the GSK3 inhibitors for 16hr. The NK cells were then incubated with AML cells and the calcein-AM assay was performed after 4 hr. Conc. = Concentration.

Supplementary figure 3

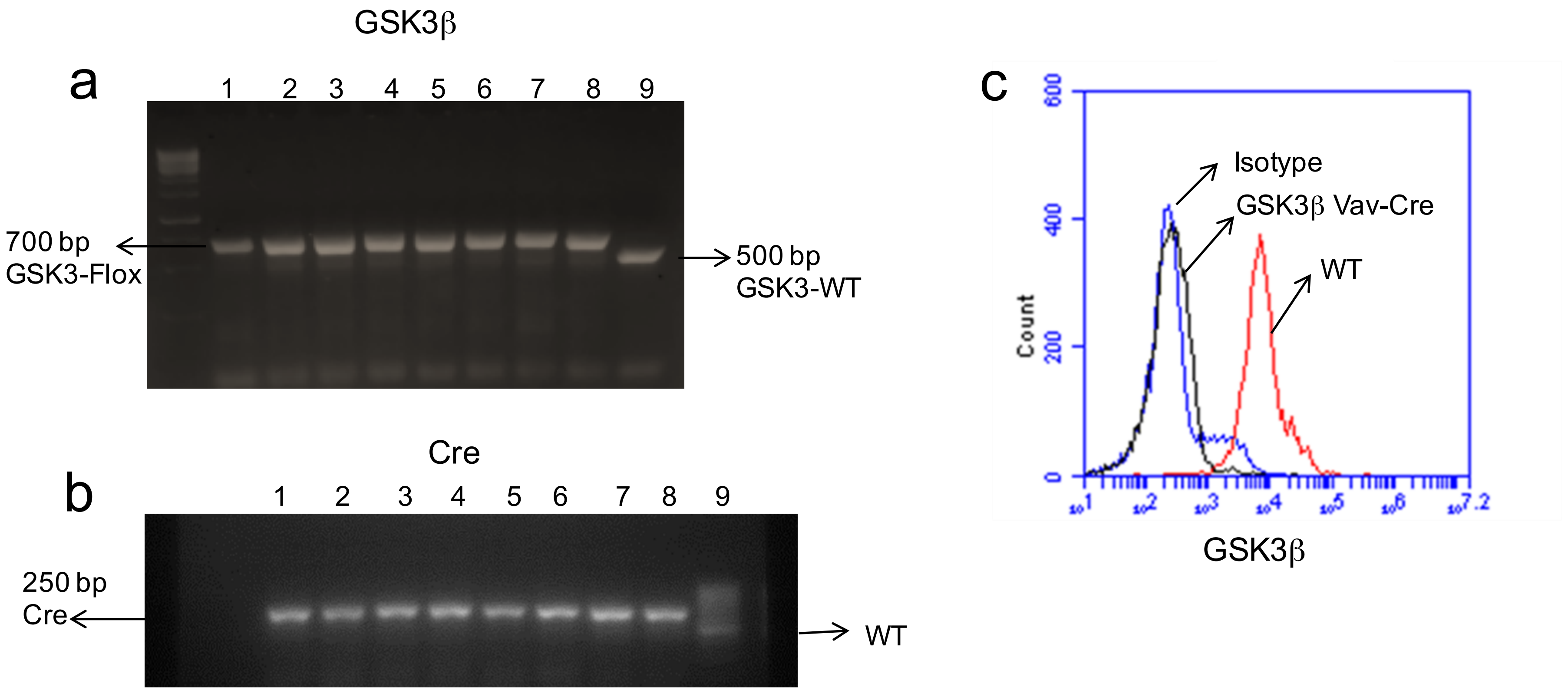

Supplementary Figure 3. Generation of mice lacking GSK3β expression in their NK cells. GSK3β-/- Vav-Cre mice were generated by crossing GSK3β floxed mice with Vav-cre mice. PCR analysis of tail DNA showing (a) GSK3β-Wild Type (500bp) and GSK3β-Flox (700bp) and (b) Cre expression (250bp). In panel A, mice 1-8: GSK3β-Flox; mouse 9 GSK3β-Wild Type. In panel B, mice 1-8: Cre positive; mouse 9: Cre negative. (c) Flow cytometric analysis showing the expression of GSK3β in Wild Type and GSK3β-Flox Vav-Cre mice.

Supplementary figure 4

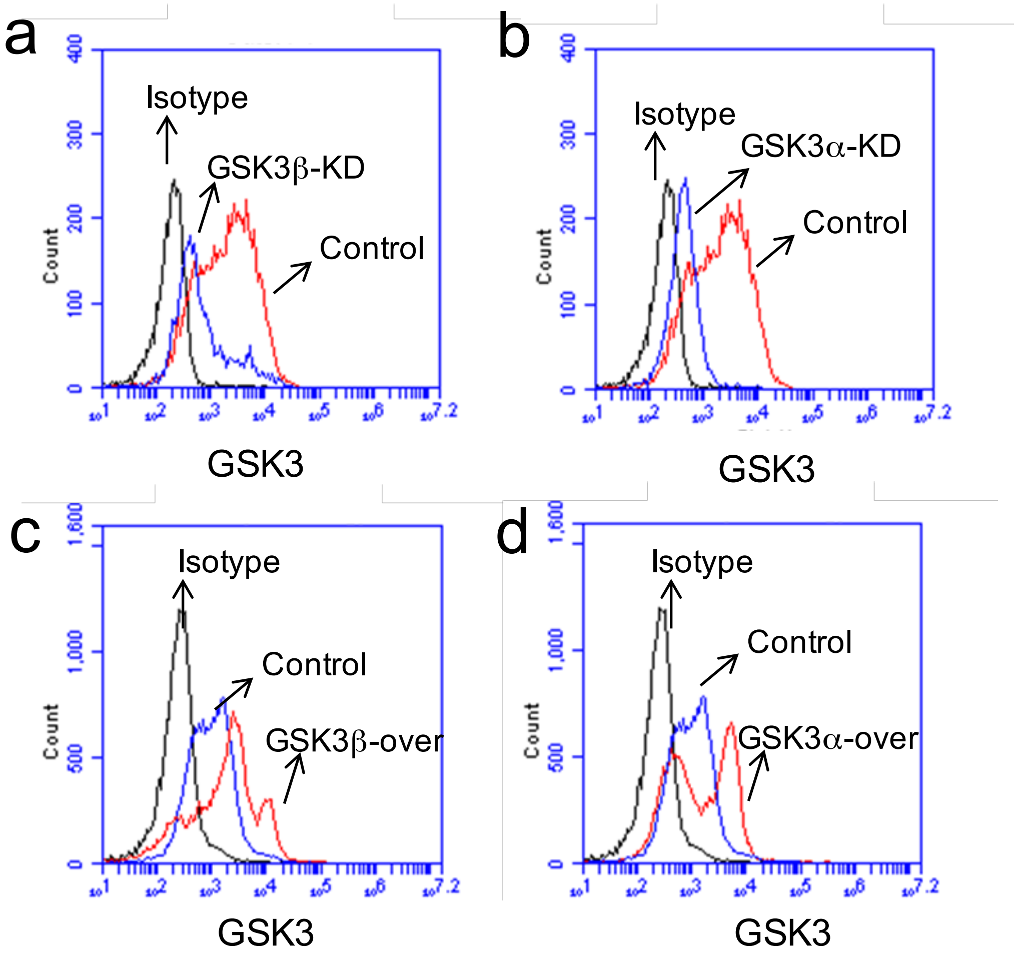

Supplementary Figure 4. GSK3 knockdown and overexpression in human NK cells. Flow cytometric analysis showing the GSK3 expression in control and (a) GSK3b Knockdown (b) GSK3a Knockdown (c) GSK3b overexpression and (d) GSK3a overexpression NK cells. KD = Knockdown; Over = Overexpression.

Supplementary figure 5

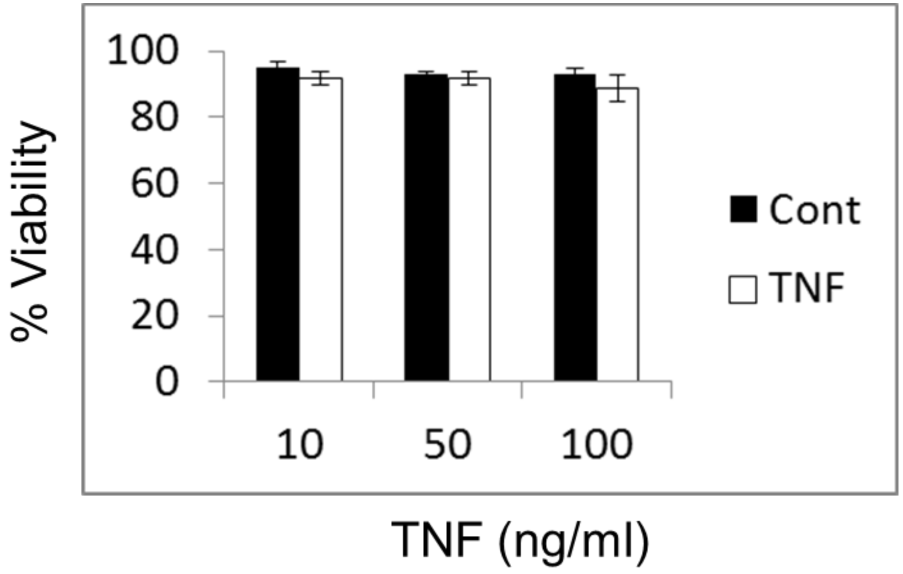

Supplementary Figure 5. TNFa does not induce apoptosis of AML cells at levels sufficient for NK cell hyperactivation. OCI-AML3 cells were treated with TNFa for 6hr at the doses indicated and assessed for viability using trypan blue exclusion.

Supplementary figure 6

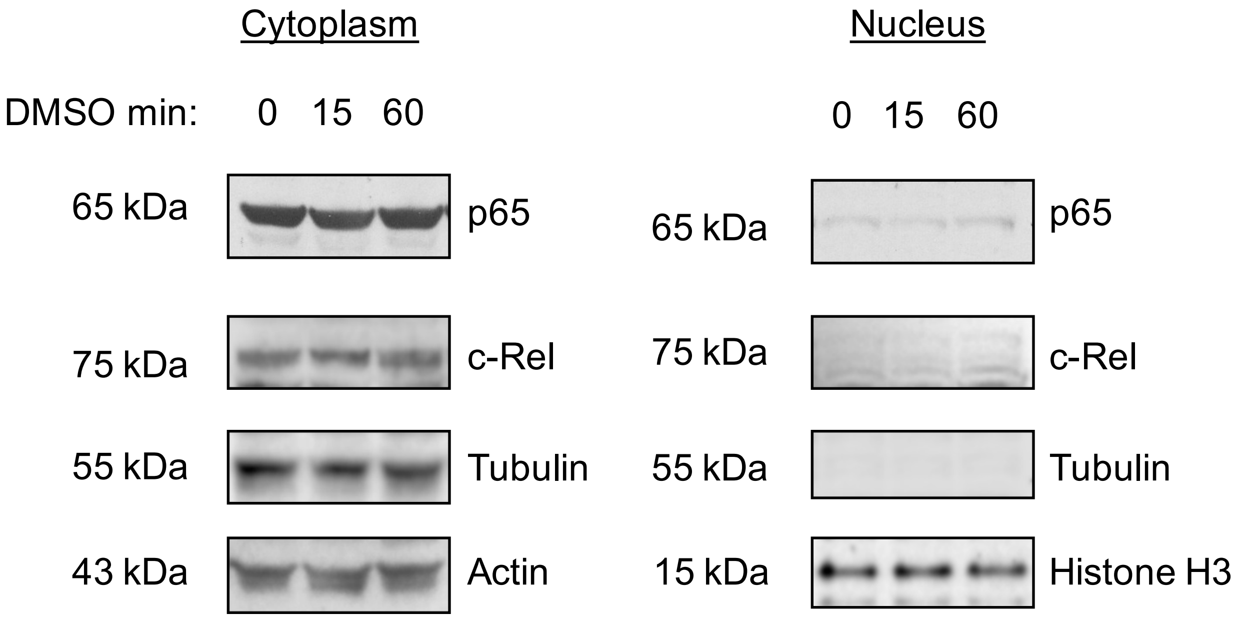

Supplementary Figure 6. NF-kB activation in NK cells. NK cells were treated with DMSO for the indicated times, cytoplasmic and nuclear lysates were prepared and probed with the indicated antibodies. Actin and Histone H3 were used as internal loading controls for cytoplasmic and nuclear extracts. Tubulin was used as a control to examine the purity of nuclear extracts.

Supplementary figure 7

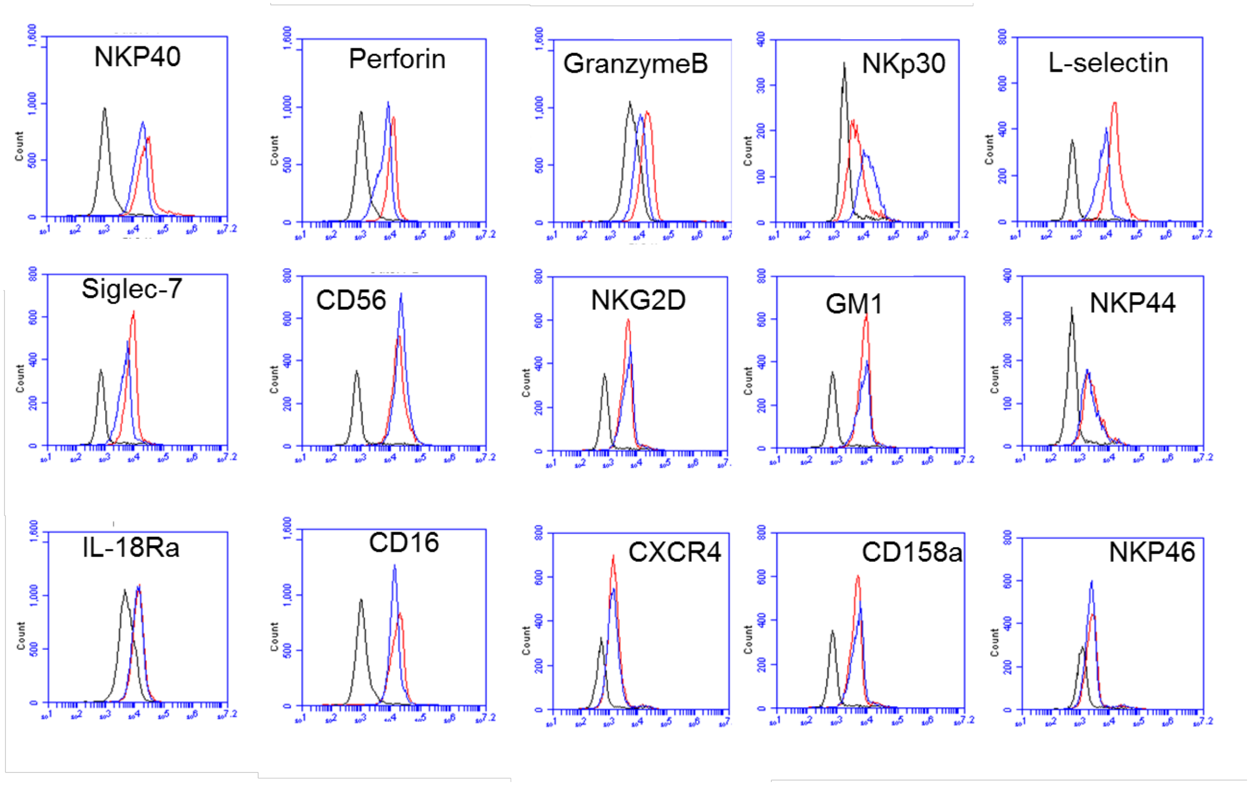

Supplementary Figure 7. GSK3 inhibitor regulation of NK cell receptors, granzyme and perforin. Expanded NK cells were treated with vehicle or SB for 16hr and then assessed for expression of the indicated proteins by flow cytometry. Red = SB; Blue = Vehicle; Black = Isotype Antibody Control.

Supplementary figure 8

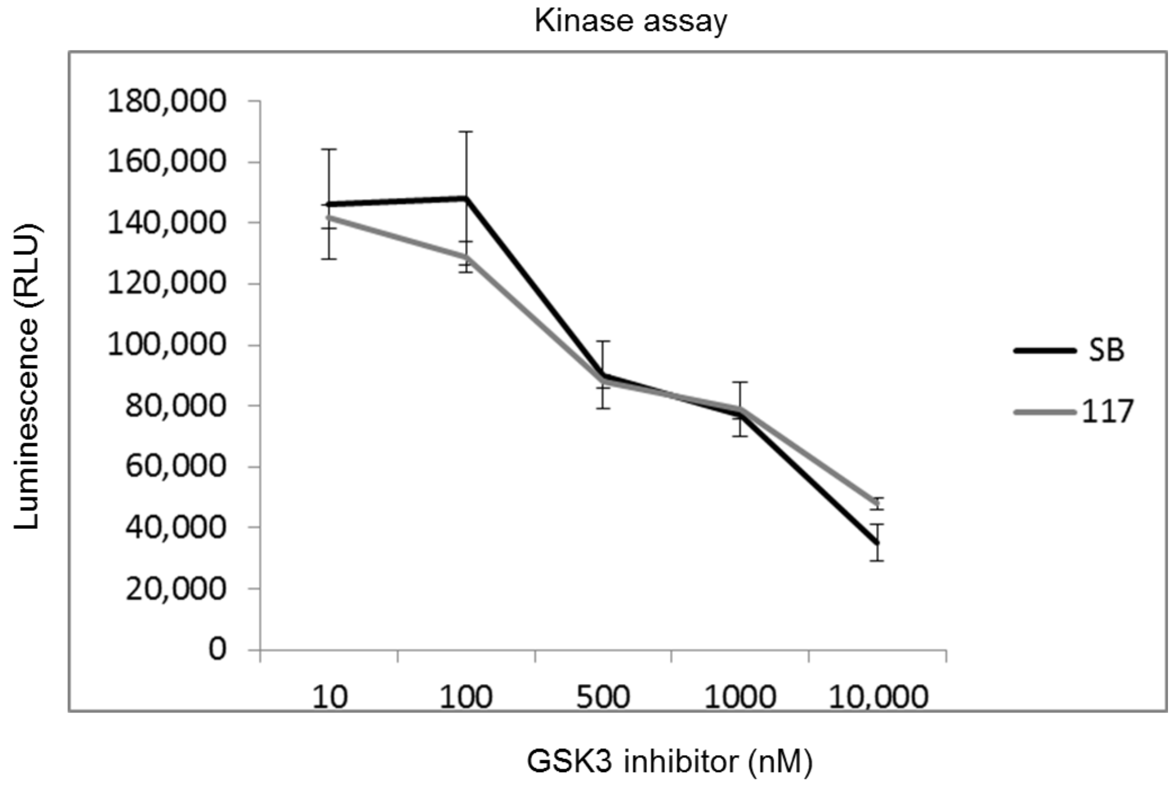

Supplementary Figure 8. 117 inhibits GSK3. 117 and SB were tested using the ADP-Glo kinase assay to assess inhibition of GSK3**.** Luminescence values represent the mean of four replicates (RLU = relative light units).

Supplementary figure 9

Compound 117-Synthetic scheme

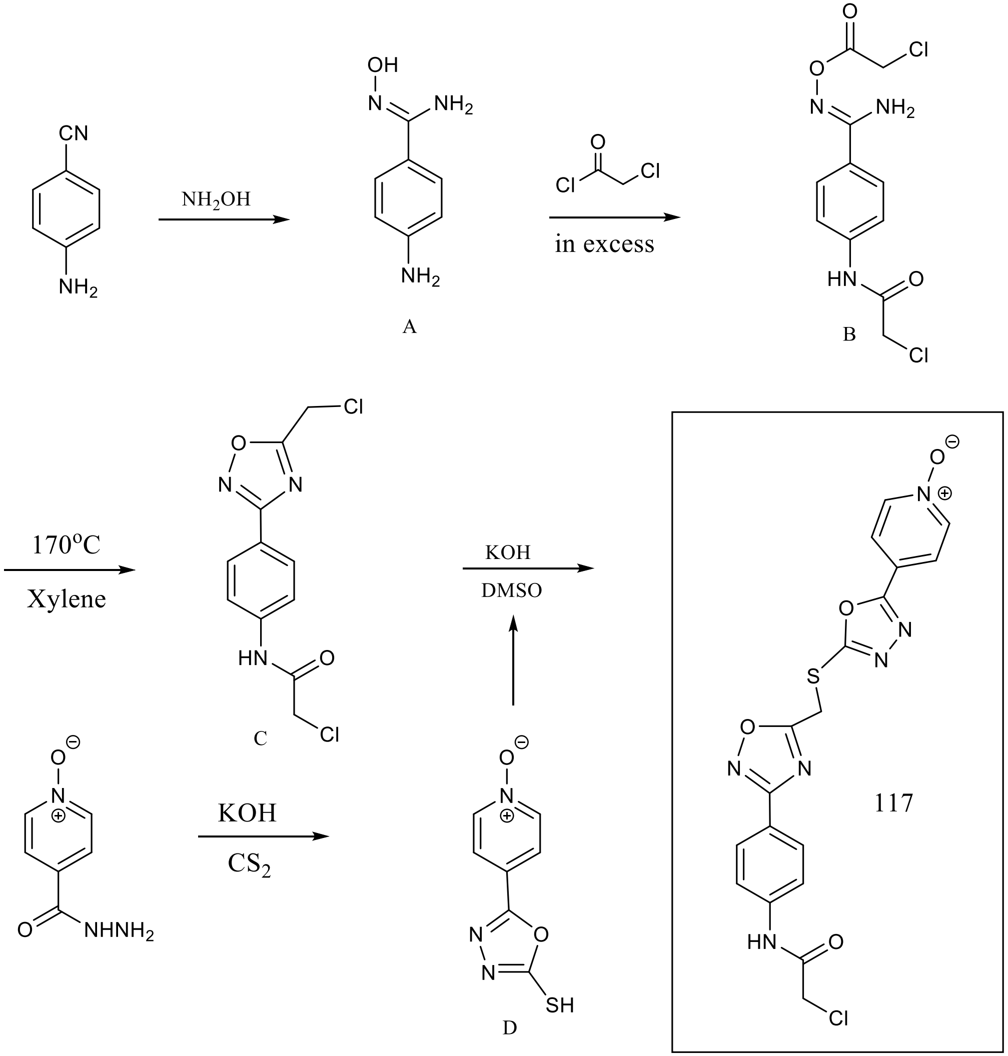

Supplementary figure 10

Western blot raw data

Figure 1b GSK3β

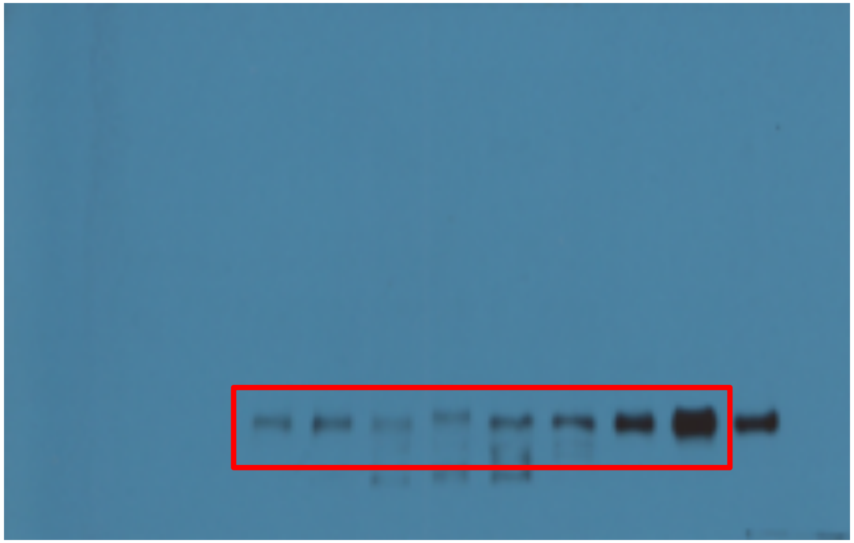

Figure 1b ERK1 and ERK2

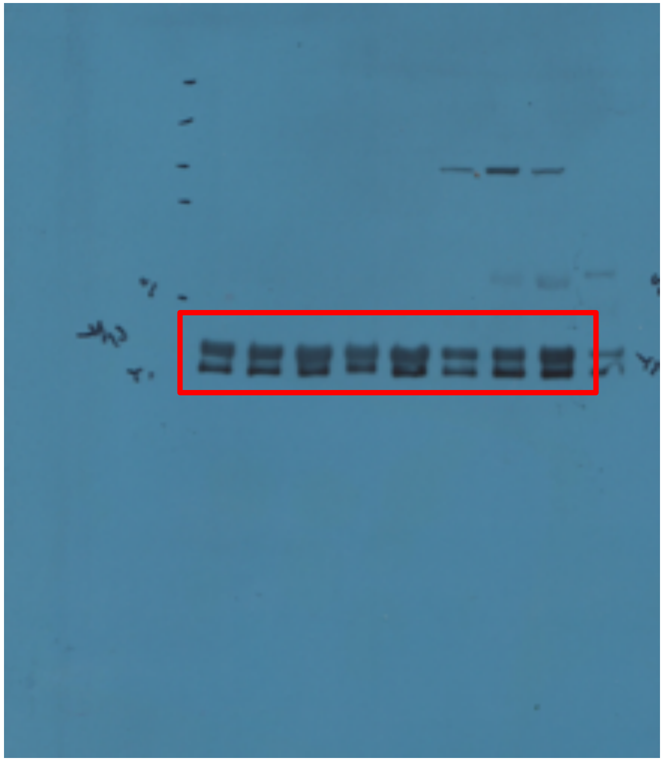

Supplementary figure 11

Figure 5a- p65 cytoplasm

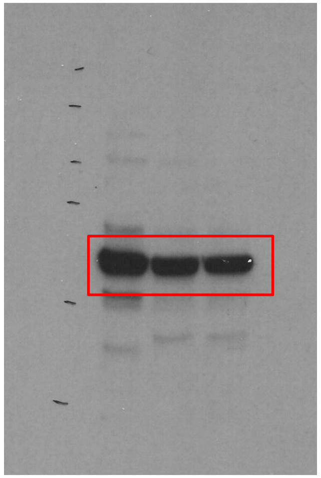

Figure 5a - p65 nucleus

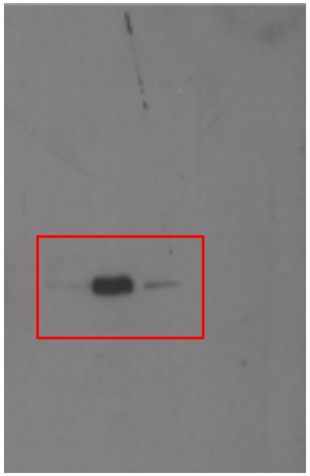

Figure 5a - cRel cytoplasm

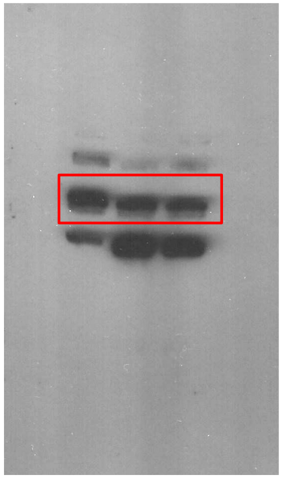

Figure 5a– cRel nucleus

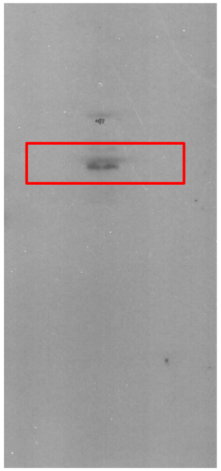

Figure 5b- p65

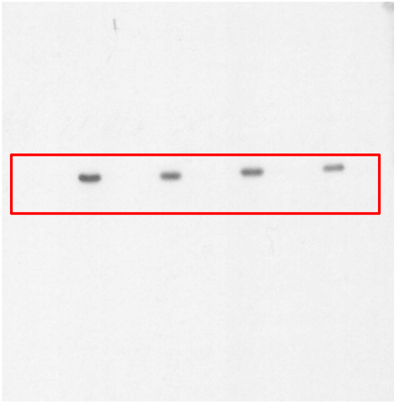

Figure 5b- p50

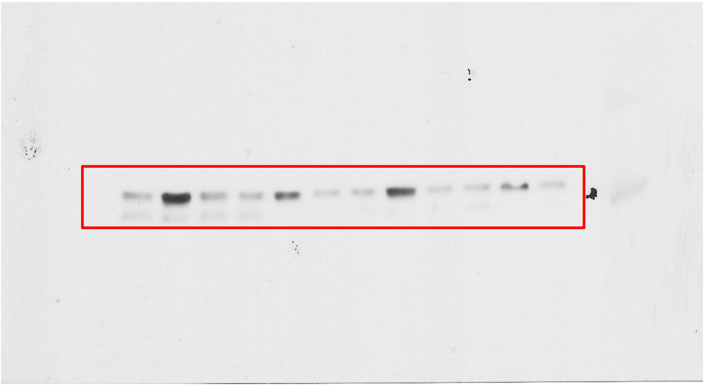

Figure 5b- c-Rel

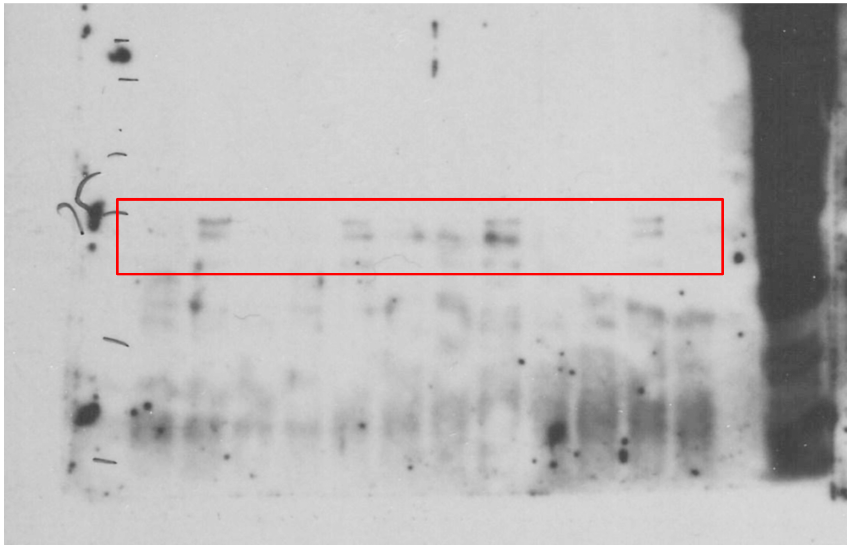

Supplementary figure 12

Raw gels of Supplementary Fig 6

Actin control DMSO

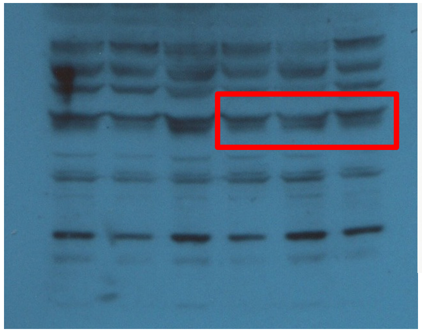

cRel-Control Nucleus DMSO

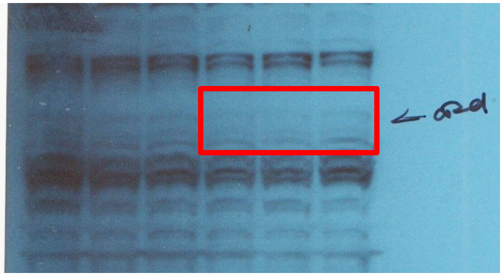

c-Rel cyto control DMSO

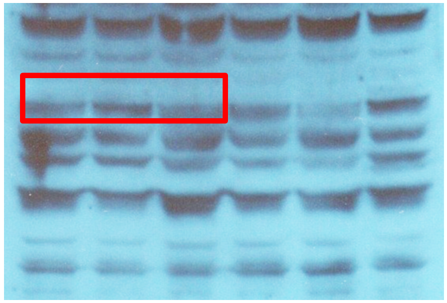

Tubulin cyto DMSO

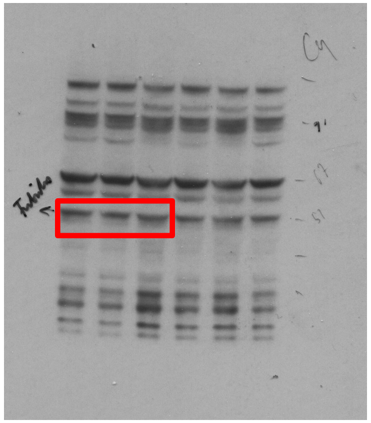

P65 control

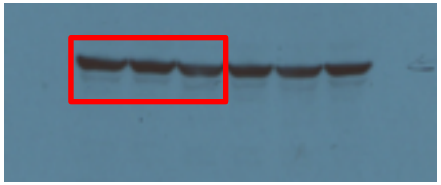

P65 nucleus control

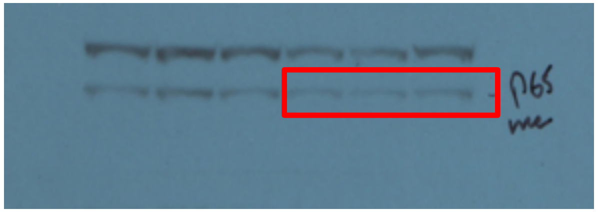

Histone H3

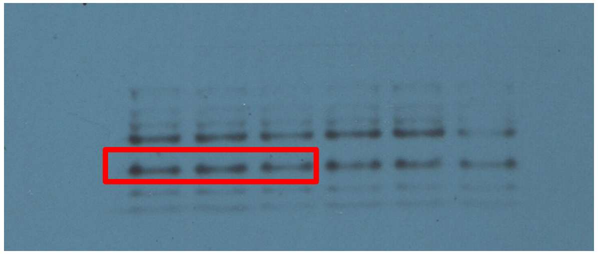

Actin cyto figure 5a

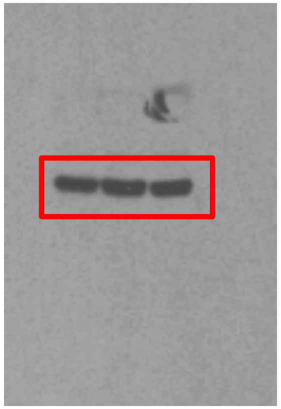

Reprobed with tubulin figure 5a

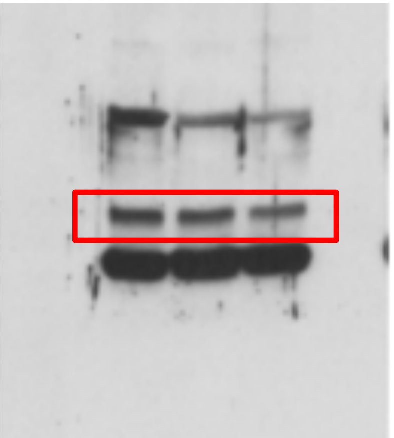

hnRNPA1-figure 5a

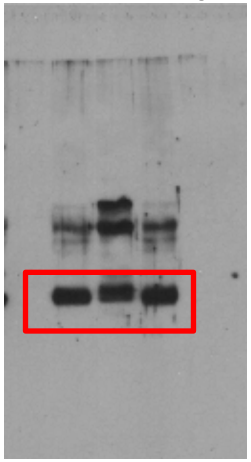

Supplementary figure 13

Oligonucleotides corresponding to NF-κB binding regions in the human TNF promoter.

kB#1 DNA probe for 5'-Biosg- tcgagGAGTAT**GGGGACCCCCCC**TTAActcga
kB#1rev- tcgagTTAAG GGG GGG TCC CCA TAC TCctcga

kB#2 DNA probe for 5'-Biosg- tcgagGGGTCT**GTGAATTCCC**GGGGGTc tcga
kB#2 DNA rev - tcgag ACCCC CGG GAA TTC ACA GAC CCctcga

kB#2a DNA probe for 5'-Biosg-tcgagTCCCC**GGGGCTGTCC**CAGGCTTc tcga
kb#2a rev- tcgag AAGCC TGG GAC AGC CCC GGG GActcga

kappa3 DNA probe for 5'-Biosg-tcgagGCTCAT**GGGTTTCTCC**ACCAAGctcga
kappa3 rev- tcgag CT TGG TGG AGA AAC CCATGA GCctcga
