# Supplementary material for: Repression of GSK3 restores NK cell cytotoxicity in AML patients
Source: Nat Commun. 2016 Apr 4;7:11154. doi: 10.1038/ncomms11154 (PMC4822012; doi:10.1038/ncomms11154)
Supplement: Supplementary Data 1 — Chemical characterization of 117 [file ncomms11154-s2.pdf]

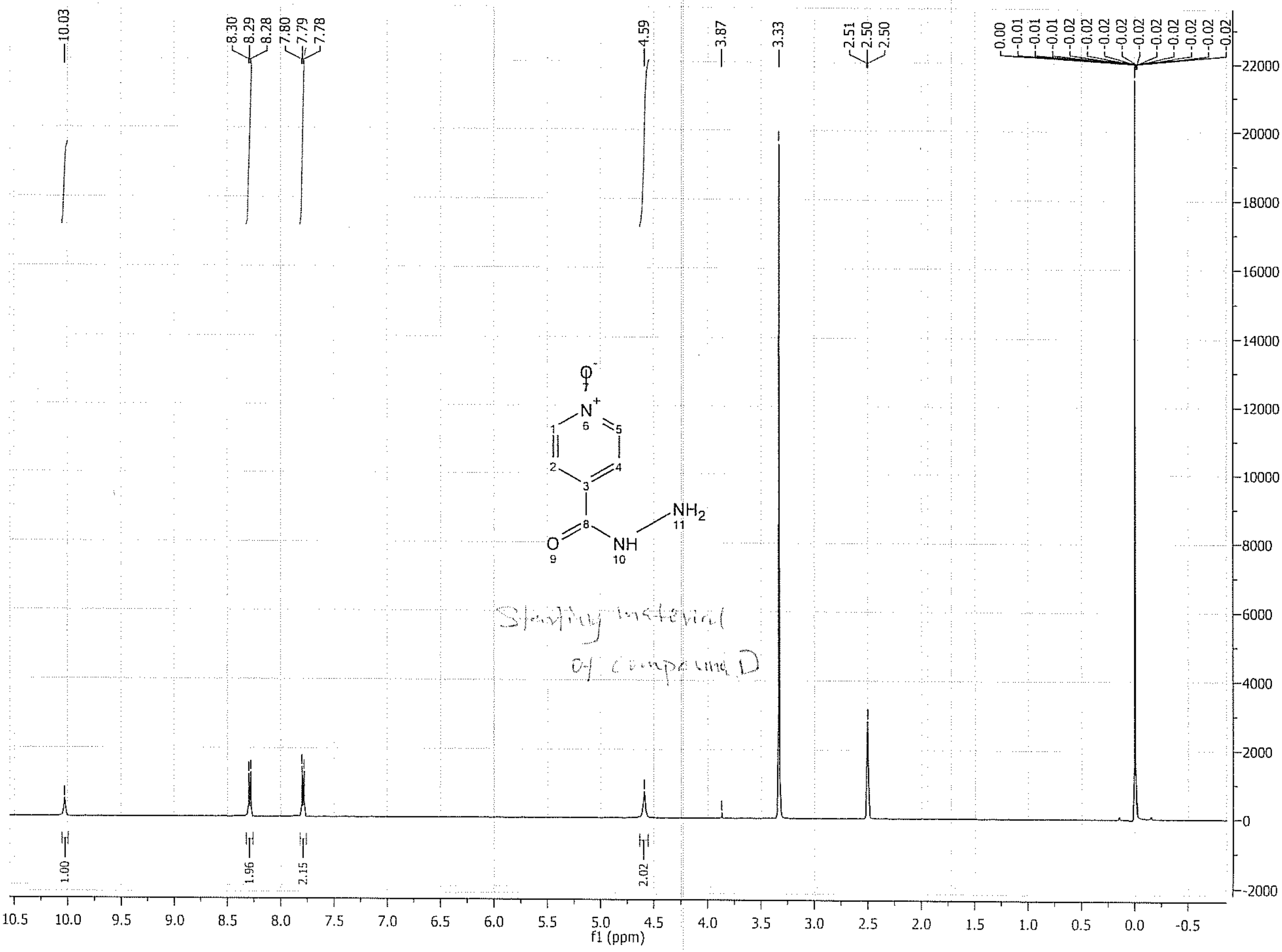
10.03
8.30
8.29
8.28
7.80
7.79
7.78
4.59
3.87
3.33
2.51
2.50
2.50
0.00
-0.01
-0.01
-0.01
-0.02
-0.02
-0.02
-0.02
-0.02
-0.02
-0.02
-0.02
-0.02
-0.02
1.00
1.96
2.15
2.02
NH_2
NH
O
N^+
O^-
Starting material
of compound D
f1 (ppm)
10.5
10.0
9.5
9.0
8.5
8.0
7.5
7.0
6.5
6.0
5.5
5.0
4.5
4.0
3.5
3.0
2.5
2.0
1.5
1.0
0.5
0.0
-0.5
22000
20000
18000
16000
14000
12000
10000
8000
6000
4000
2000
0
-2000

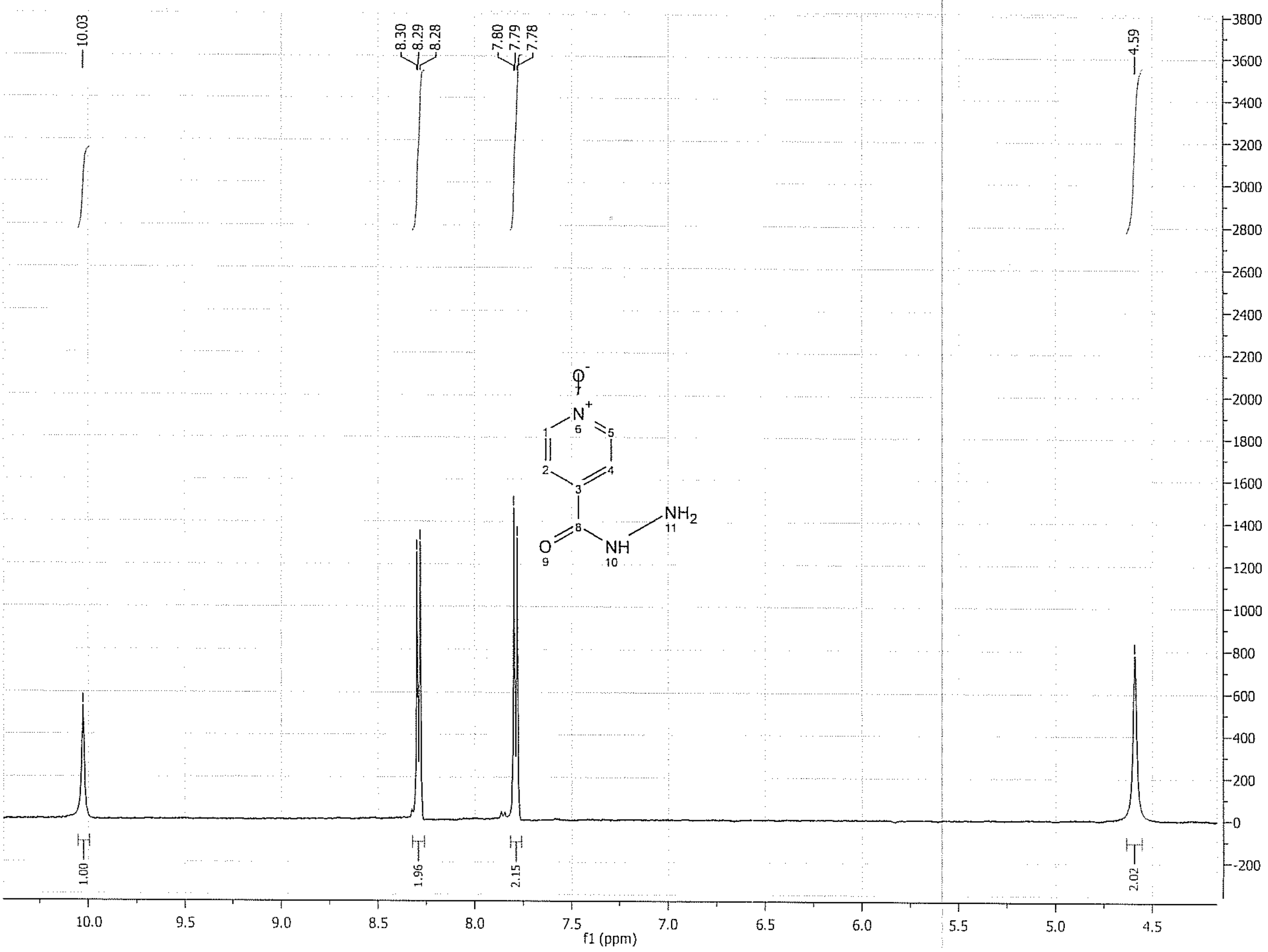

10.03
8.30
8.29
8.28
7.80
7.79
7.78
4.59
NH2
NH
O
N+
O-
1.00
1.96
2.15
2.02
f1 (ppm)

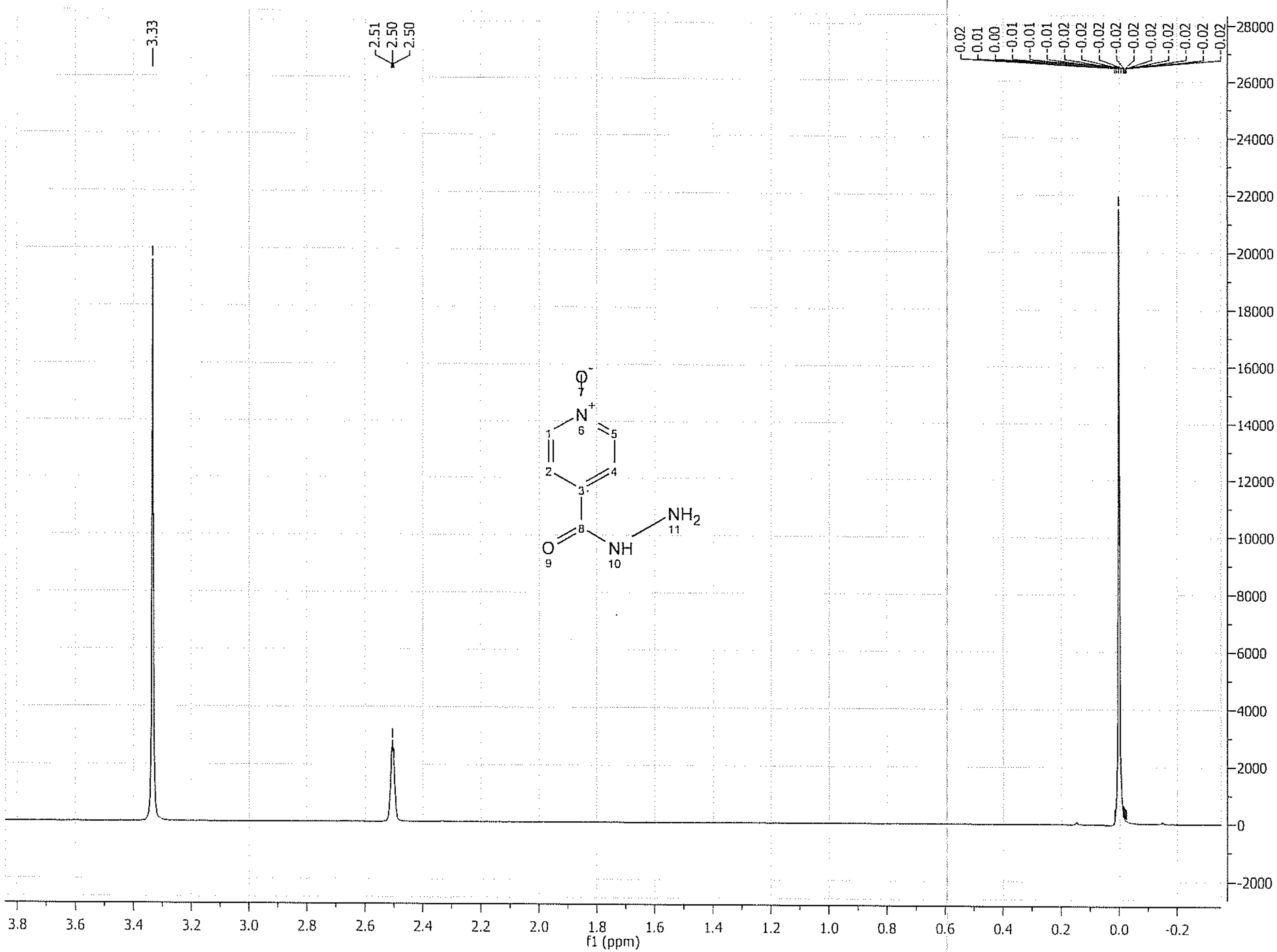

3.33
2.51
2.50
2.50
0.02
0.01
0.00
-0.01
-0.01
-0.01
-0.02
-0.02
-0.02
-0.02
-0.02
-0.02
-0.02
-0.02
-0.02
-0.02
O
7
N
6
1
2
3
4
5
8
O
9
NH
10
NH2
11
28000
26000
24000
22000
20000
18000
16000
14000
12000
10000
8000
6000
4000
2000
0
-2000
3.8
3.6
3.4
3.2
3.0
2.8
2.6
2.4
2.2
2.0
1.8
1.6
1.4
1.2
1.0
0.8
0.6
0.4
0.2
0.0
-0.2
f1 (ppm)

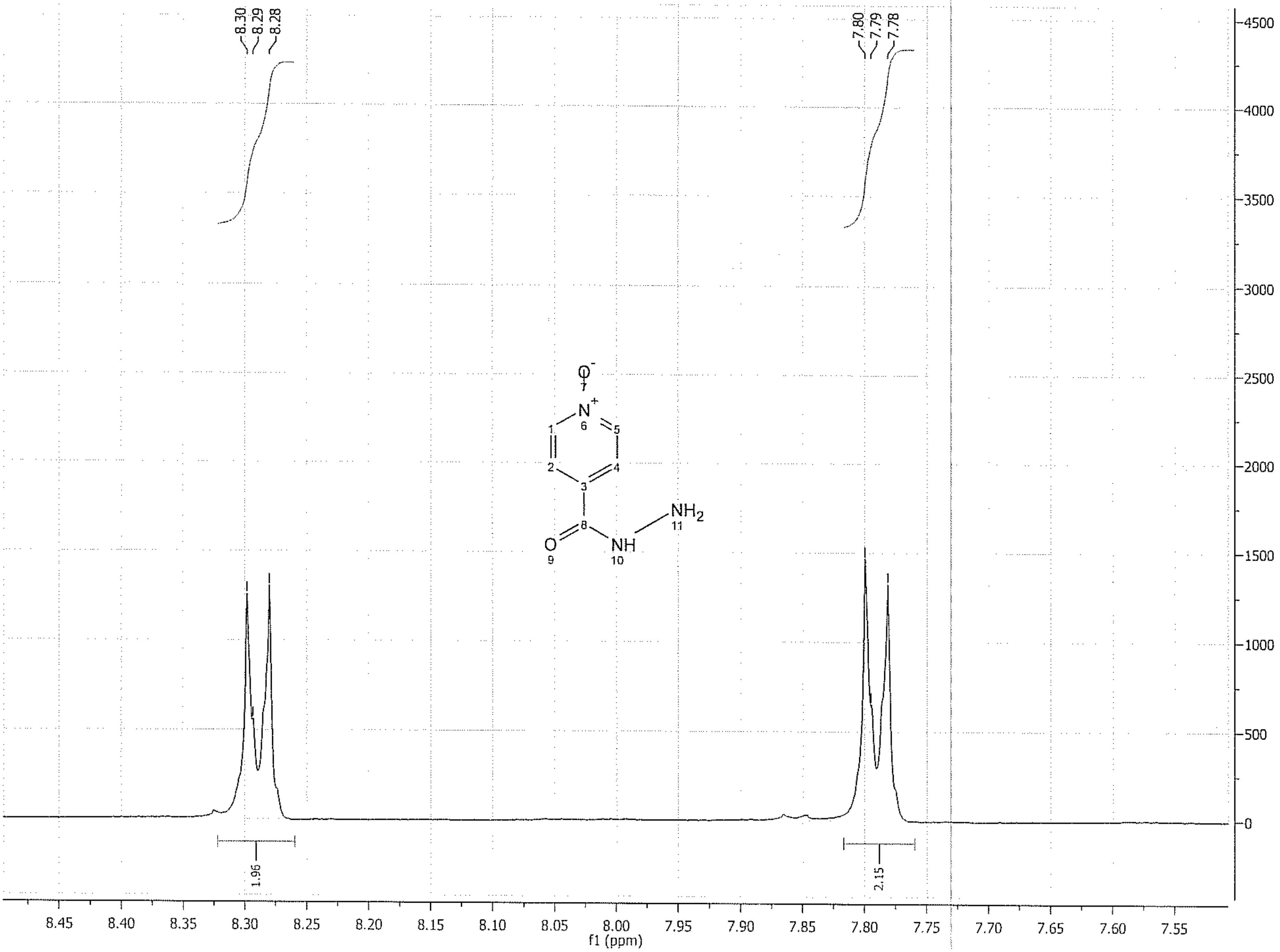

8.30
8.29
8.28
7.80
7.79
7.78
1.96
2.15
4500
4000
3500
3000
2500
2000
1500
1000
500
0
8.45
8.40
8.35
8.30
8.25
8.20
8.15
8.10
8.05
8.00
7.95
7.90
7.85
7.80
7.75
7.70
7.65
7.60
7.55
f1 (ppm)
O
N+
NH
NH2

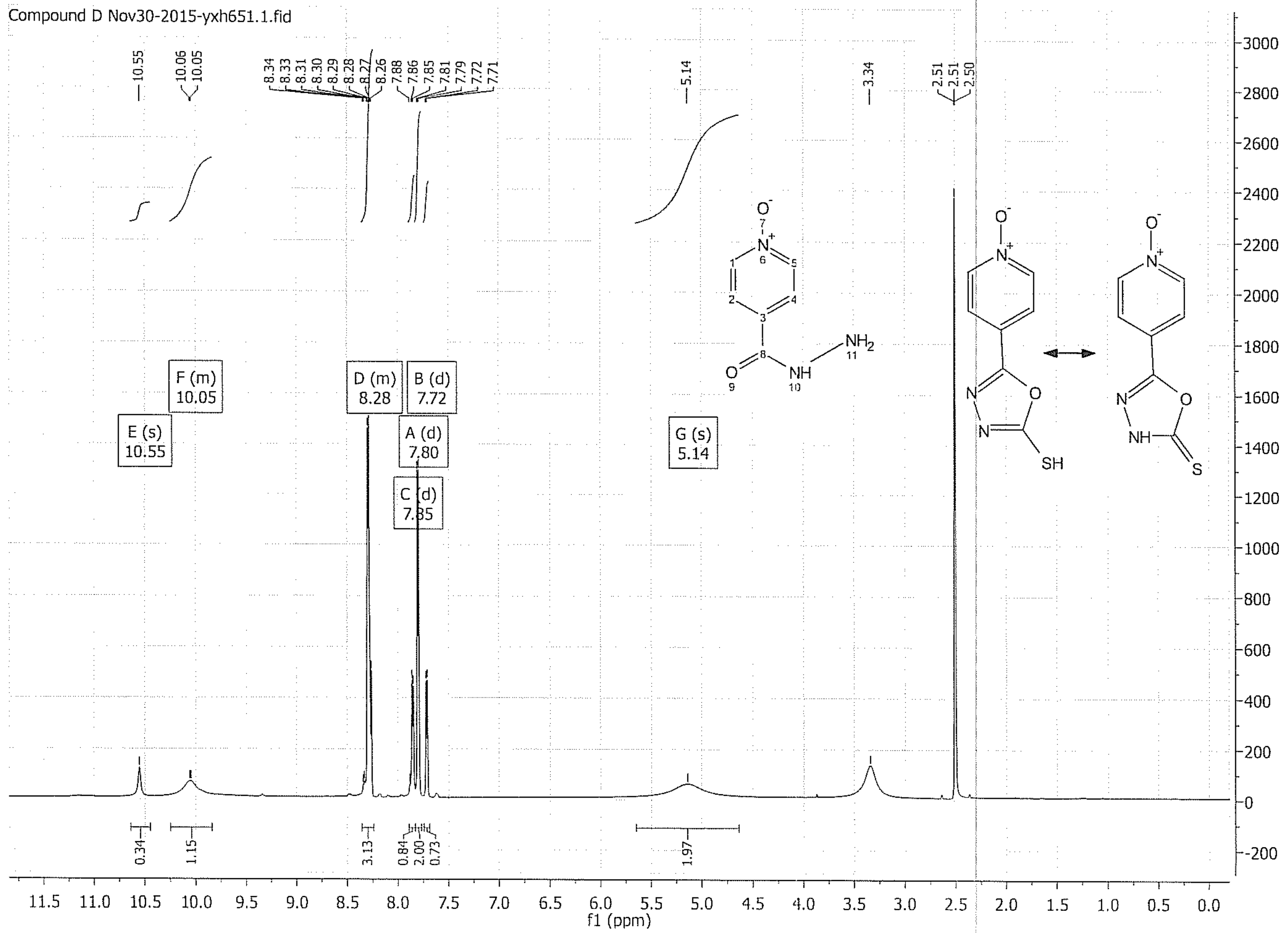
Compound D Nov30-2015-yxh651.1.fid
10.55
10.06
10.05
8.34
8.33
8.31
8.30
8.29
8.28
8.27
8.26
7.88
7.86
7.85
7.81
7.79
7.72
7.71
5.14
3.34
2.51
2.51
2.50
F (m)
10.05
E (s)
10.55
D (m)
8.28
B (d)
7.72
A (d)
7.80
C (d)
7.85
G (s)
5.14
0.34
1.15
3.13
0.84
2.00
0.73
1.97
NH
NH2
SH
S
f1 (ppm)

Compound D Nov30-2015-yxh651.1.fid

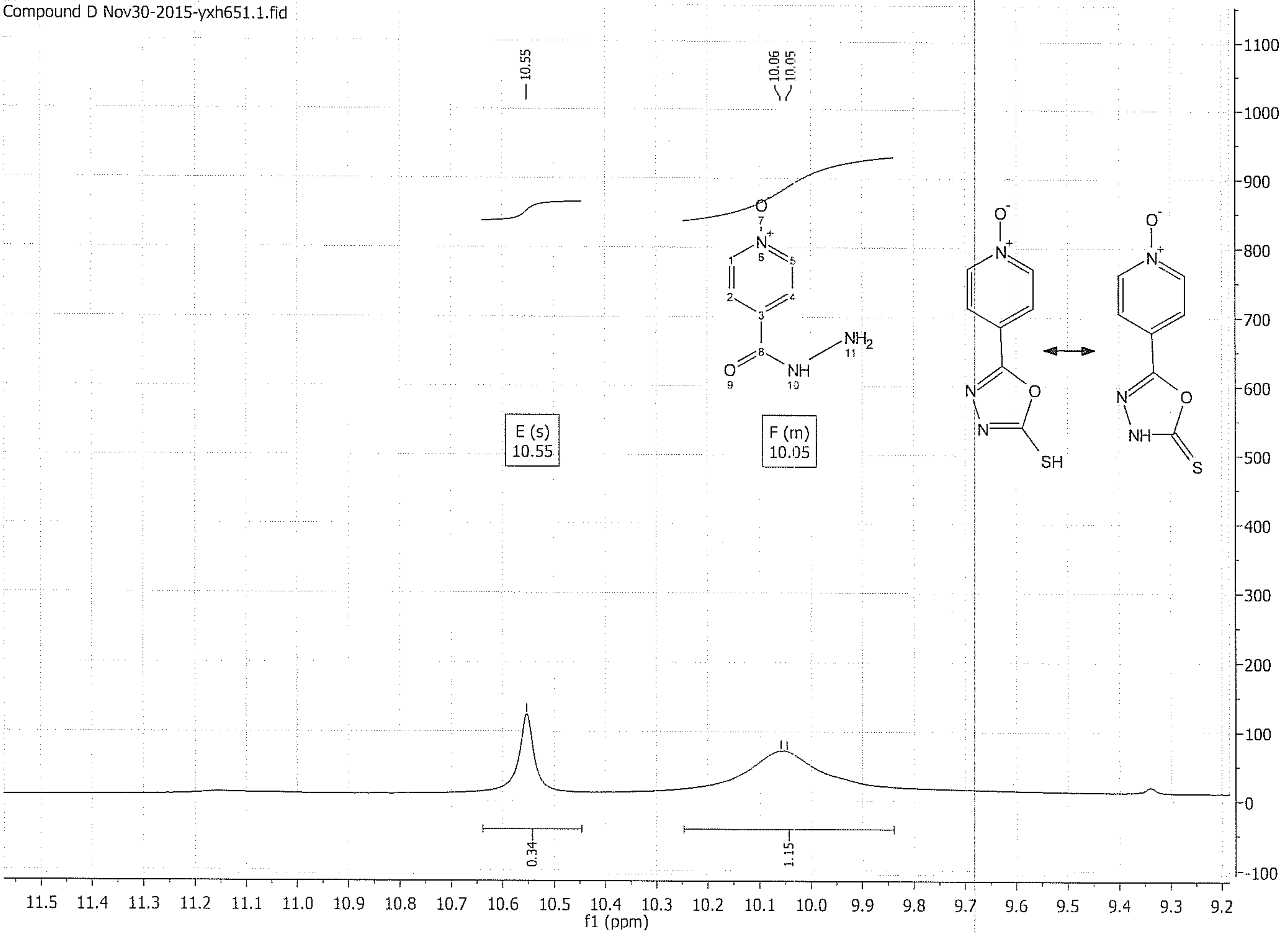

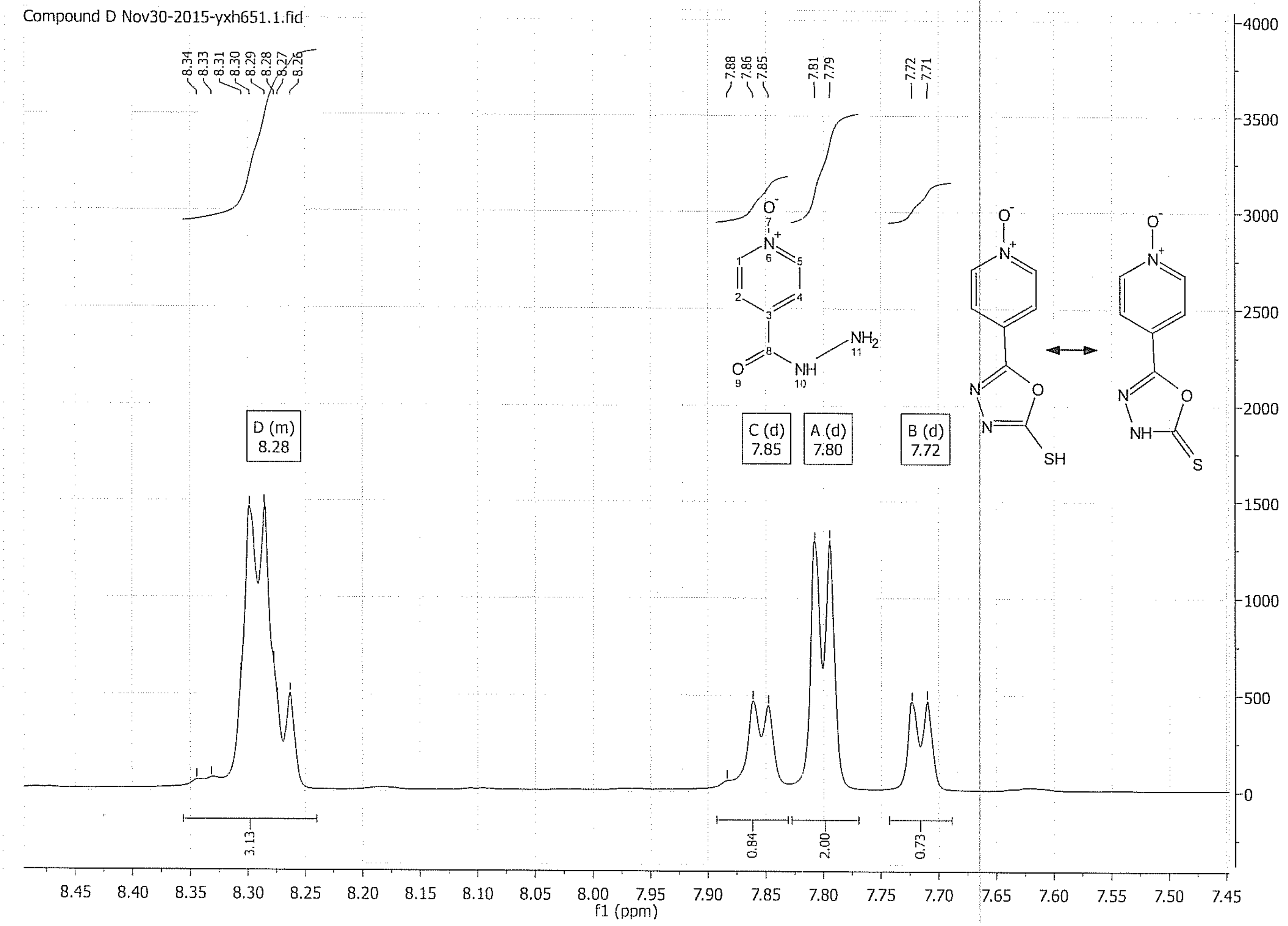

Compound D Nov30-2015-yxh651.1.fid
8.34 8.33 8.31 8.30 8.29 8.28 8.27 8.26
7.88 7.86 7.85
7.81 7.79
7.72 7.71
D (m) 8.28
C (d) 7.85
A (d) 7.80
B (d) 7.72
3.13
0.84
2.00
0.73
f1 (ppm)
SH
S

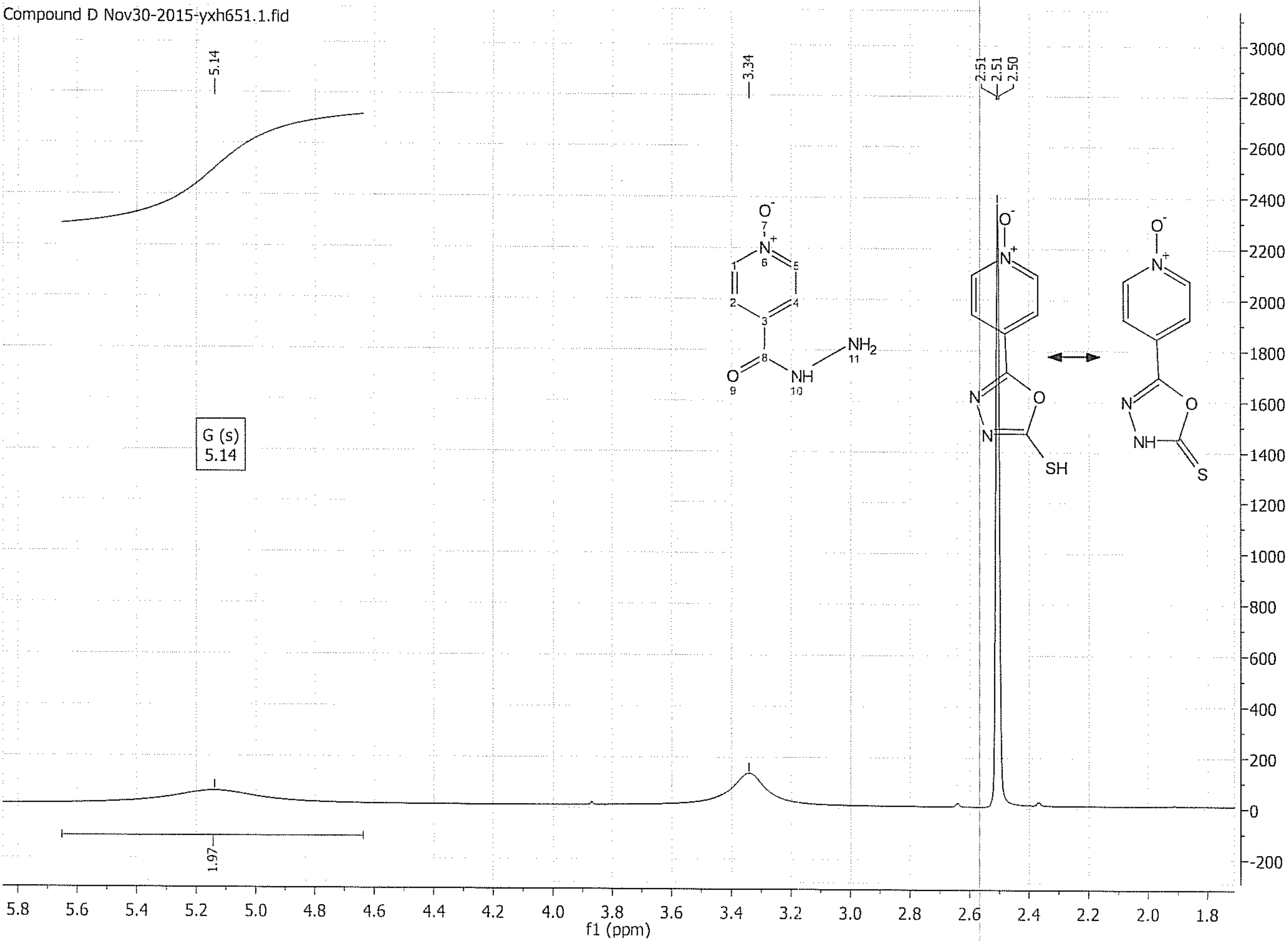
Compound D Nov30-2015-yxh651.1.fid
5.14
3.34
2.51
2.51
2.50
G (s)
5.14
1.97
f1 (ppm)
NH2
NH
O
N
SH
S

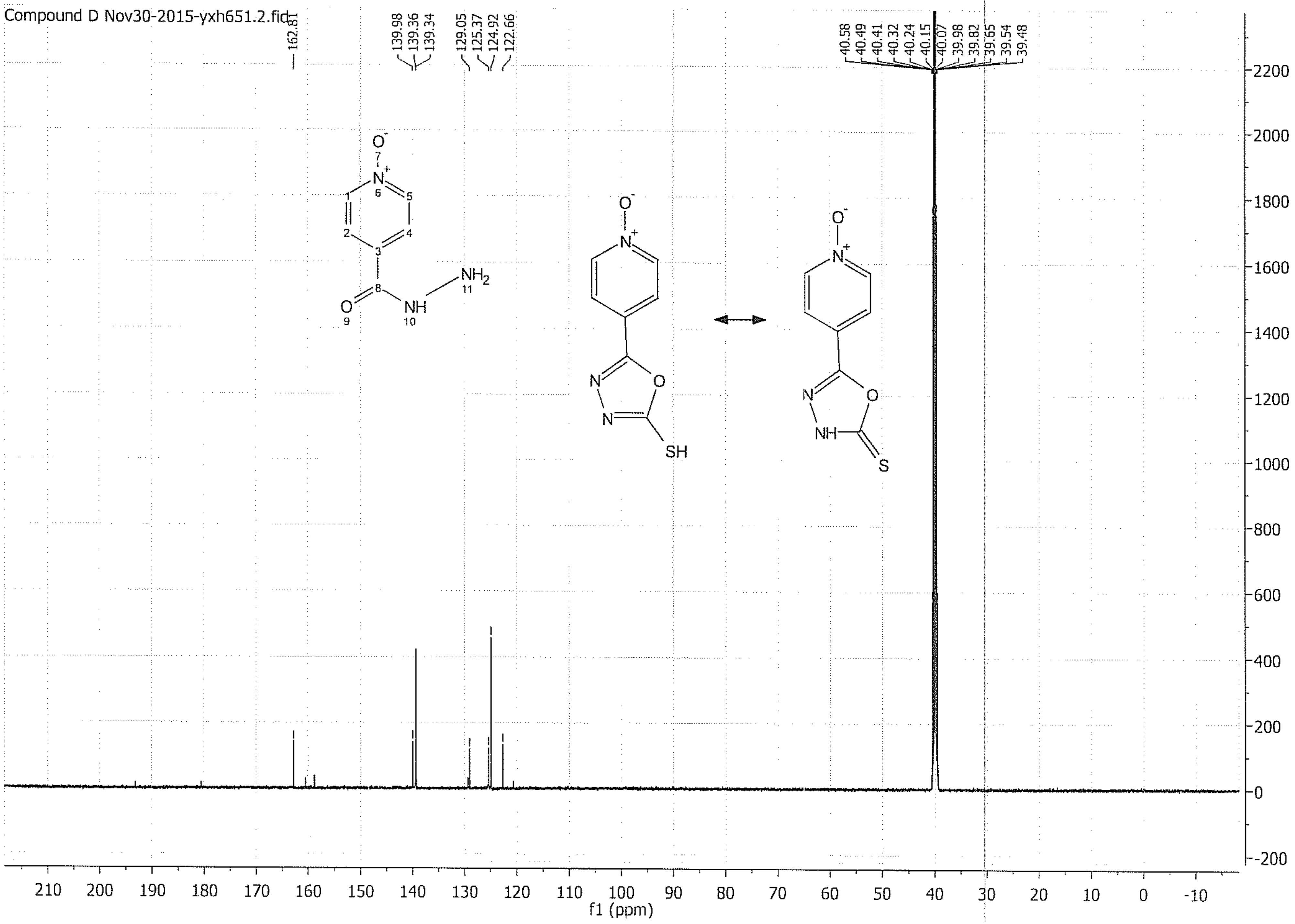

Compound D Nov30-2015-yxh651.2.fid
162.81
139.98
139.36
139.34
129.05
125.37
124.92
122.66
40.58
40.49
40.41
40.32
40.24
40.15
40.07
39.98
39.82
39.65
39.54
39.48
O
N
NH
NH
NH
SH
S
f1 (ppm)

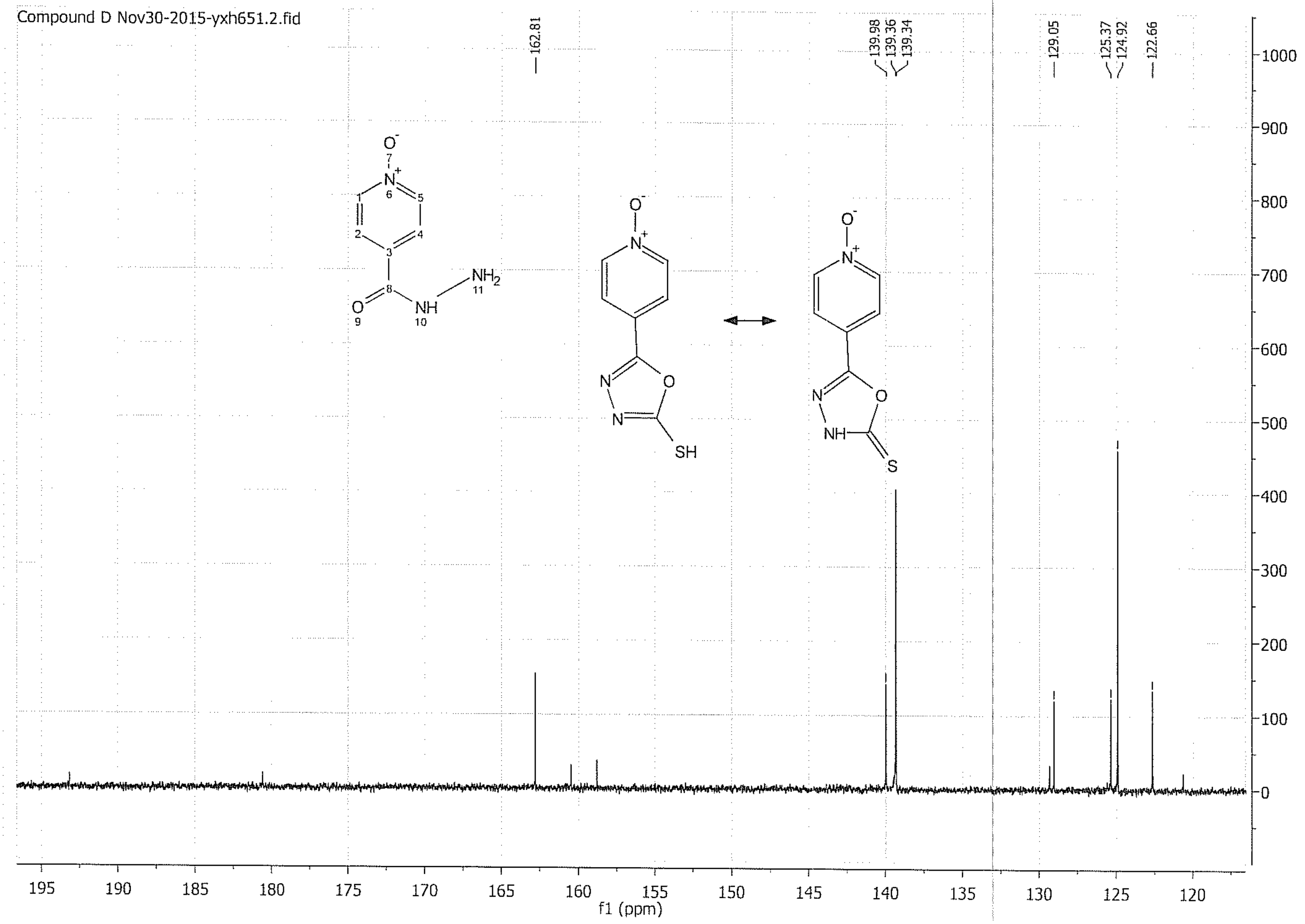
Compound D Nov30-2015-yxh651.2.fid
162.81
139.98
139.36
139.34
129.05
125.37
124.92
122.66
f1 (ppm)

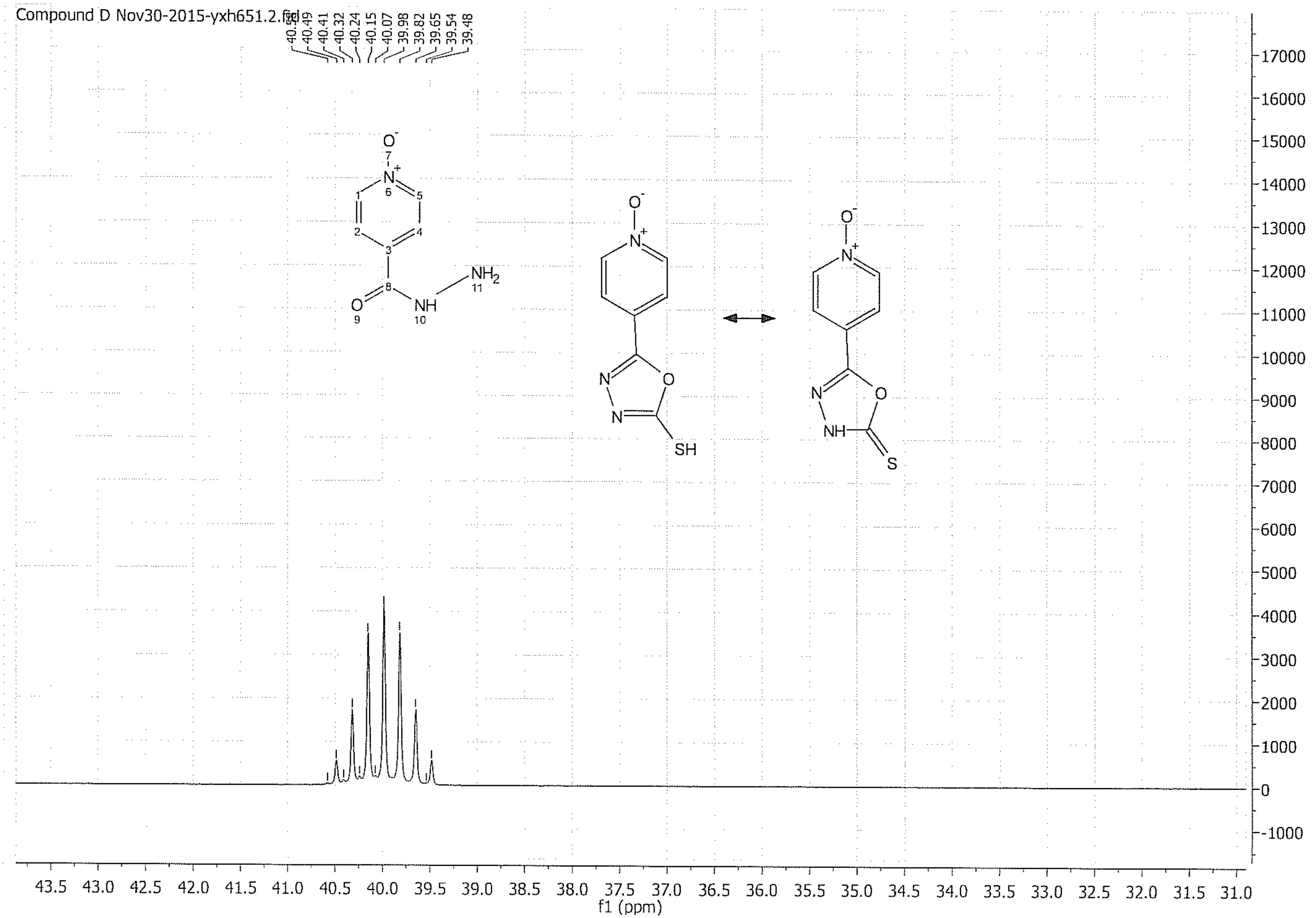

Compound D Nov30-2015-yxh651.2.fid
40.58
40.49
40.41
40.32
40.24
40.15
40.07
39.98
39.82
39.65
39.54
39.48
O
N
NH
NH2
SH
S
f1 (ppm)

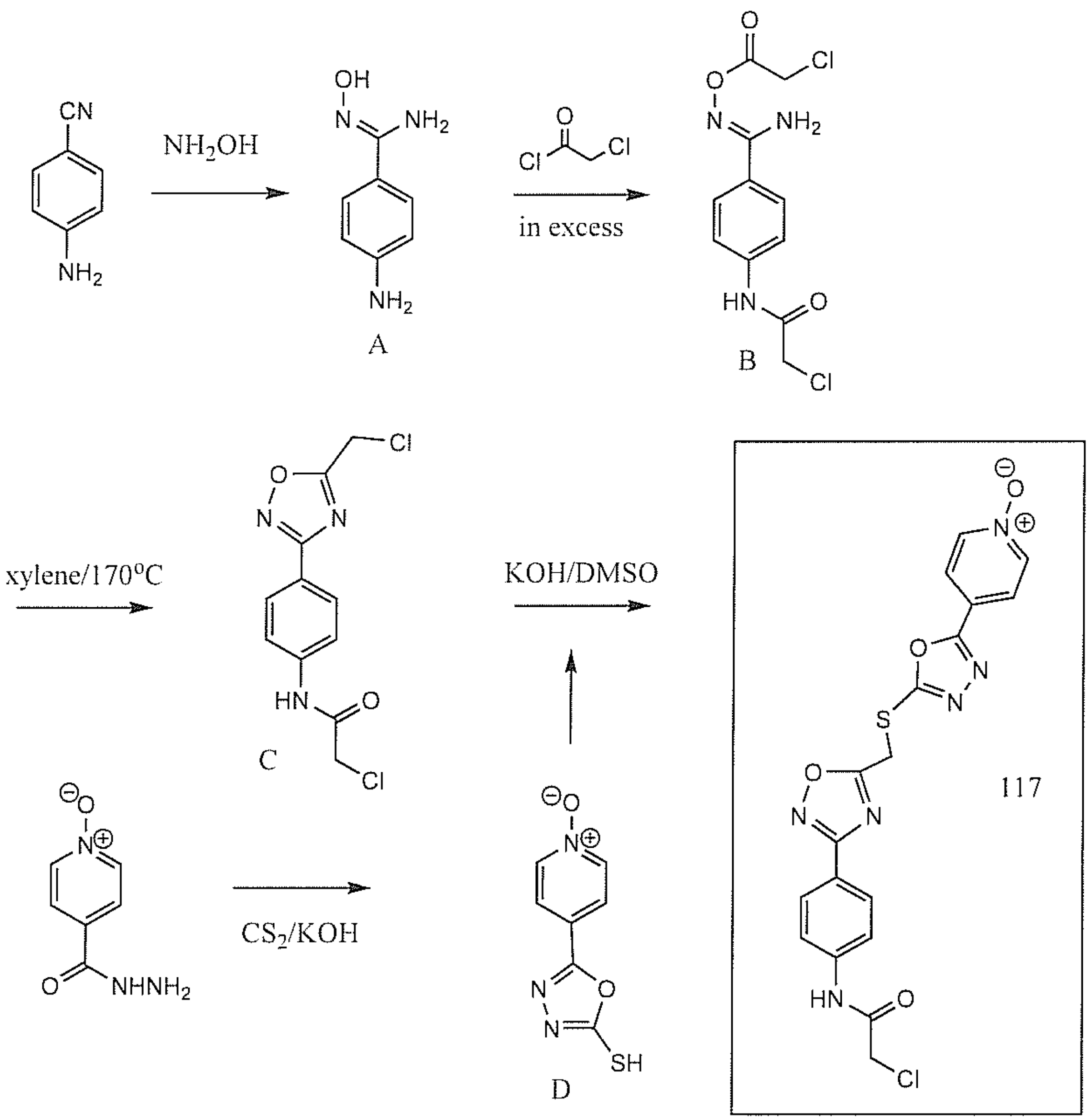

Synthetic Scheme for the Synthesis of Compound 117

compound A Nov30-2015-yxh651.1.fid
Compound A for 117

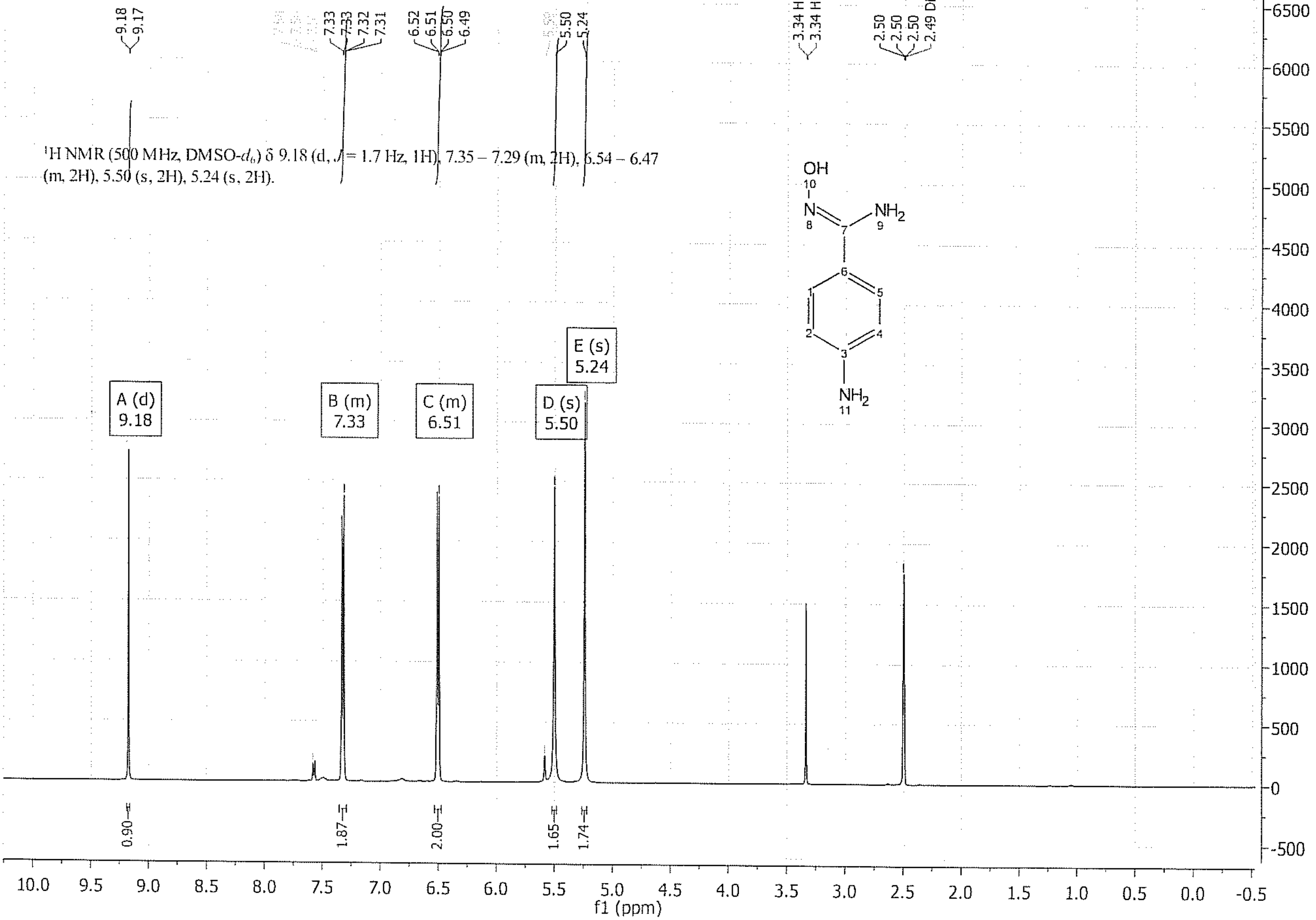

^{1}H NMR (500 MHz, DMSO-d_6) δ 9.18 (d, J = 1.7 Hz, 1H), 7.35 – 7.29 (m, 2H), 6.54 – 6.47 (m, 2H), 5.50 (s, 2H), 5.24 (s, 2H).

compound A Nov30-2015-yxh651.1.fid
Compound A for 117

^{1}H NMR (500 MHz, DMSO-d_6) δ 9.18 (d, J = 1.7 Hz, 1H), 7.35 – 7.29 (m, 2H), 6.54 – 6.47 (m, 2H), 5.50 (s, 2H), 5.24 (s, 2H).

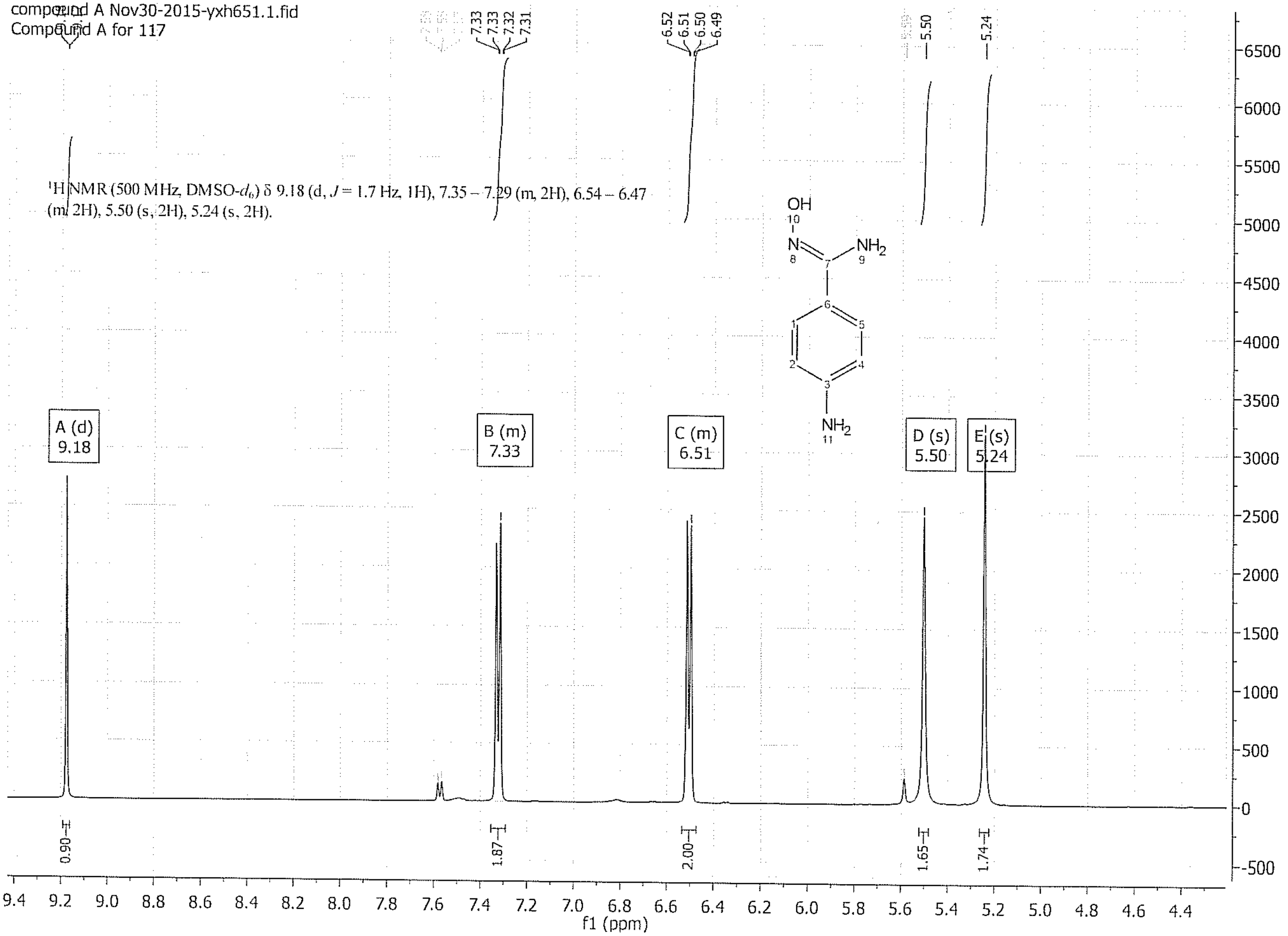

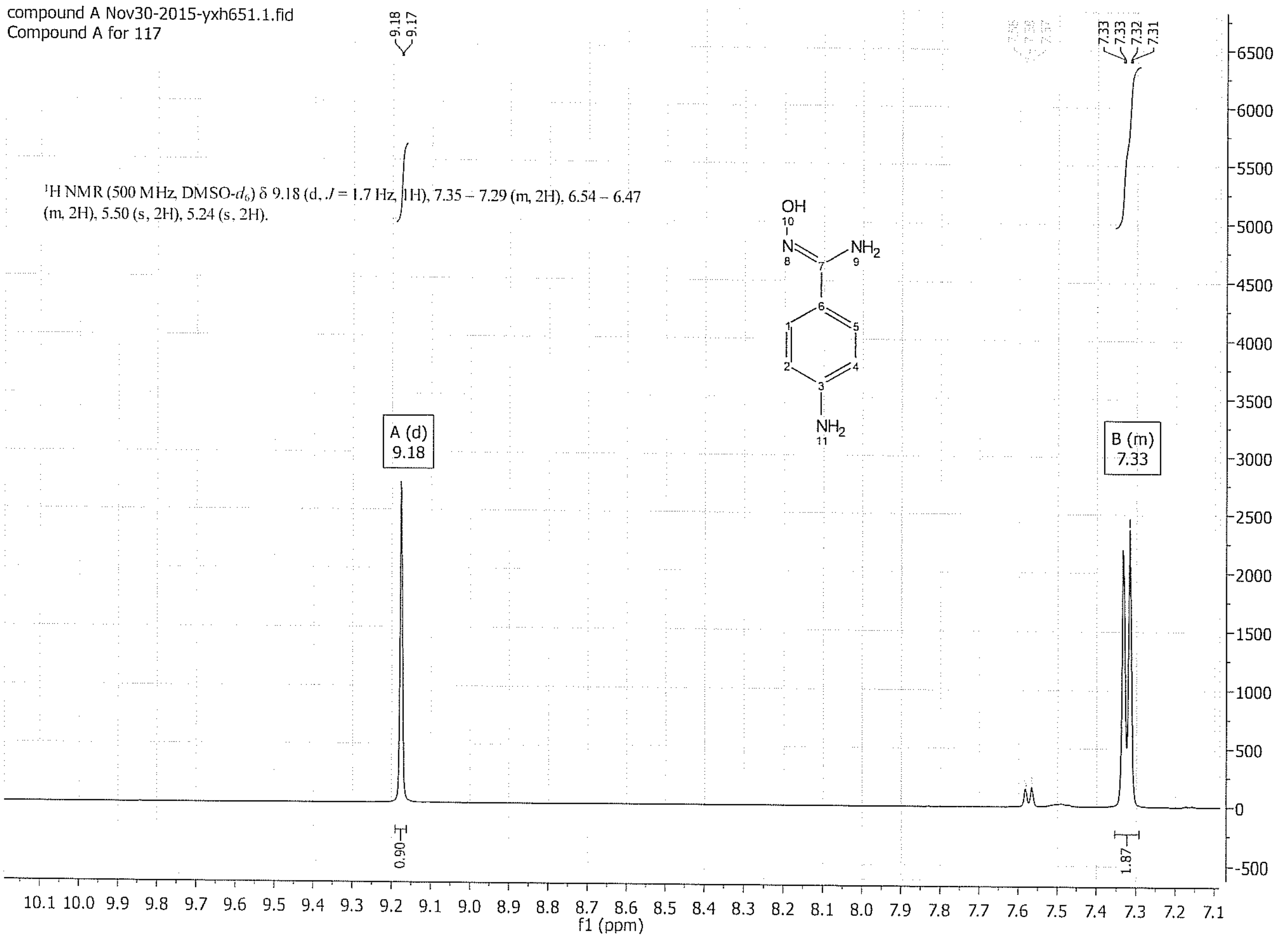
compound A Nov30-2015-yxh651.1.fid
Compound A for 117
9.18
9.17
7.33
7.33
7.32
7.31
^{1}H NMR (500 MHz, DMSO-d_6) δ 9.18 (d, J = 1.7 Hz, 1H), 7.35 – 7.29 (m, 2H), 6.54 – 6.47 (m, 2H), 5.50 (s, 2H), 5.24 (s, 2H).
OH
NH_2
NH_2
A (d)
9.18
B (m)
7.33
0.90
1.87
f1 (ppm)

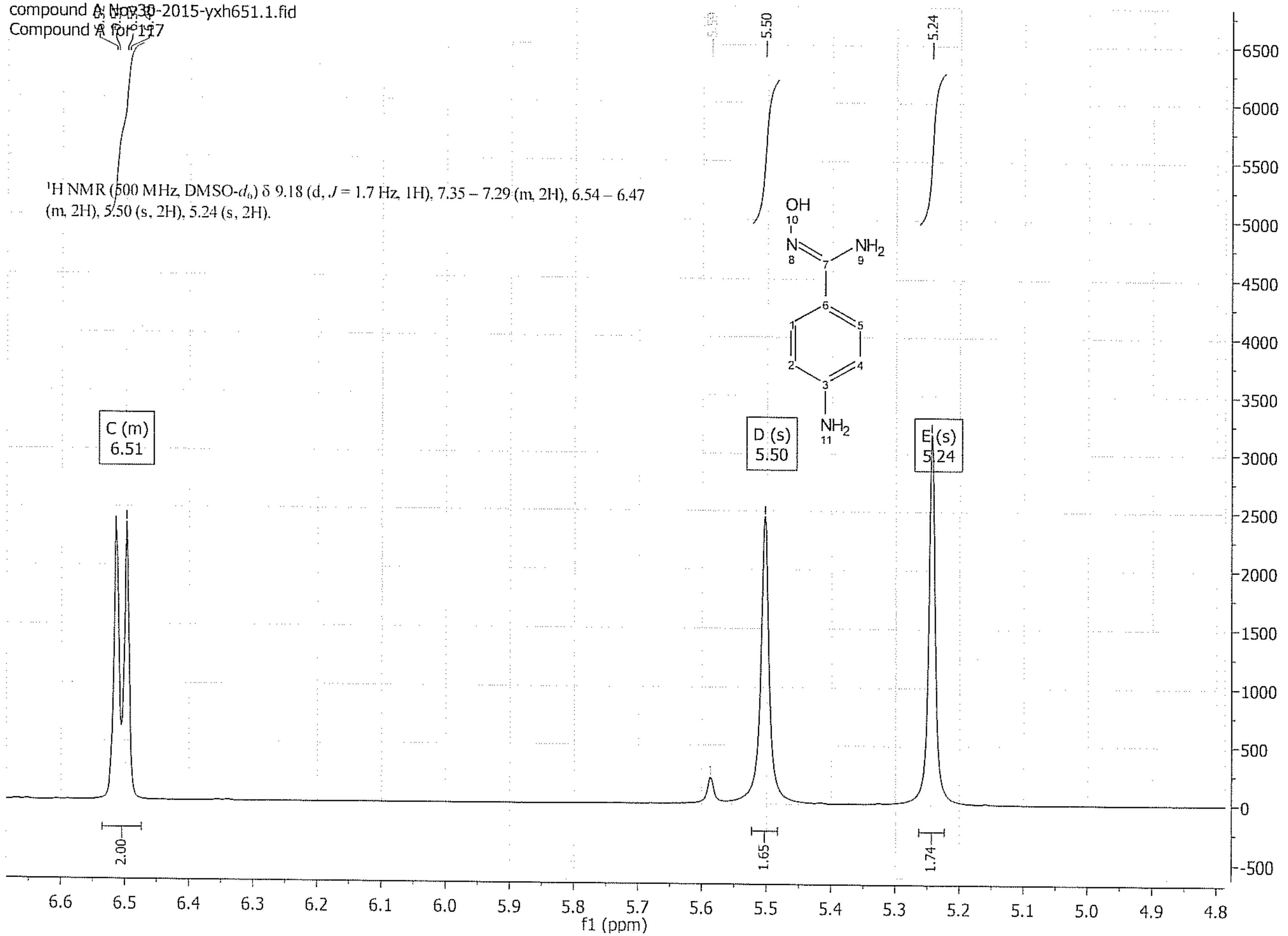

^{1}H NMR (500 MHz, DMSO-d_6) δ 9.18 (d, J = 1.7 Hz, 1H), 7.35 – 7.29 (m, 2H), 6.54 – 6.47 (m, 2H), 5.50 (s, 2H), 5.24 (s, 2H).

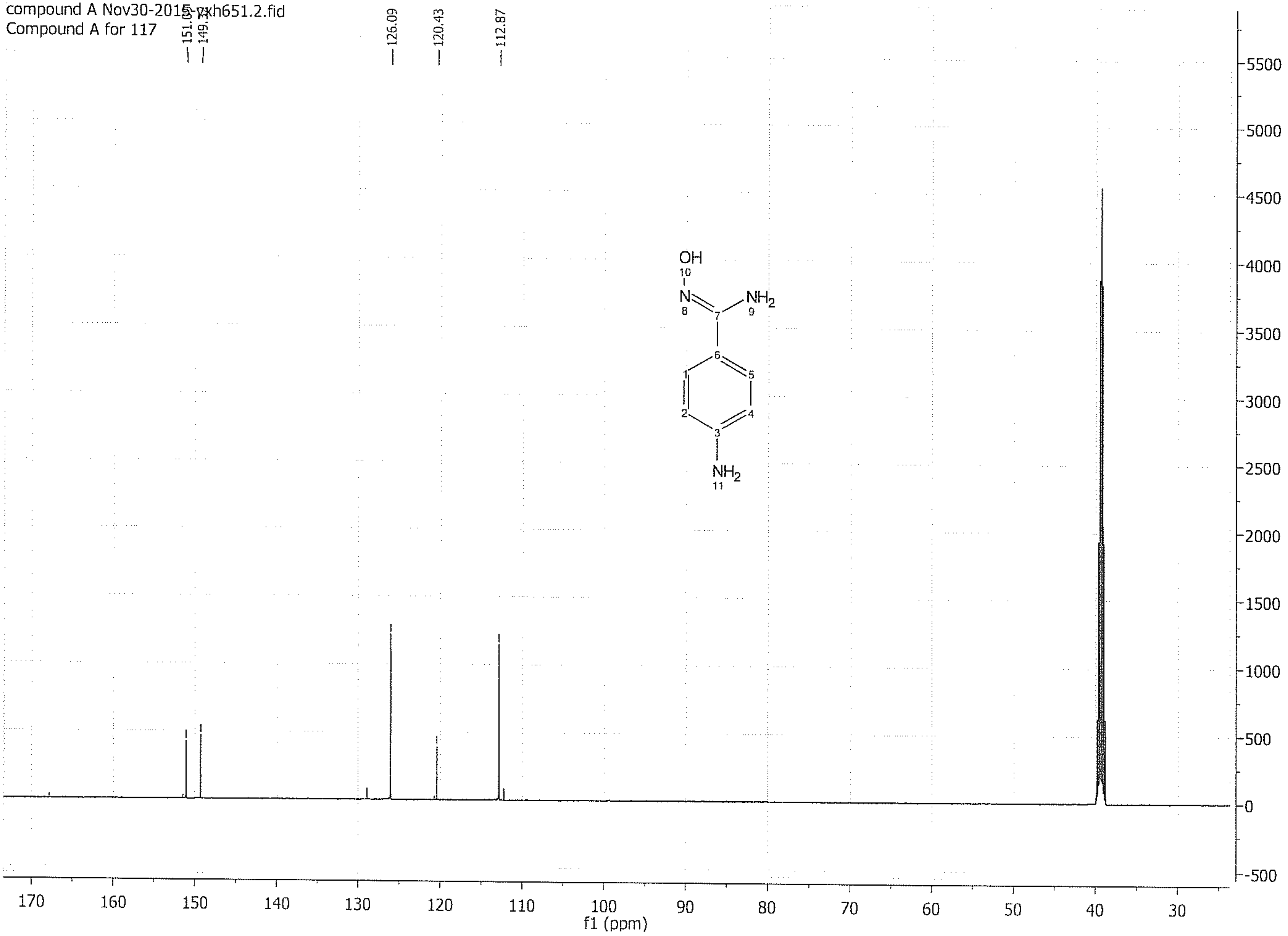
compound A Nov30-2015-yxh651.2.fid
Compound A for 117
151.0
149.
126.09
120.43
112.87
OH
10
N
8
NH2
9
7
6
1
5
2
4
3
NH2
11
5500
5000
4500
4000
3500
3000
2500
2000
1500
1000
500
0
-500
170
160
150
140
130
120
110
100
90
80
70
60
50
40
30
f1 (ppm)

==== Shimadzu LabSolutions Data Report ====

<Chromatogram>

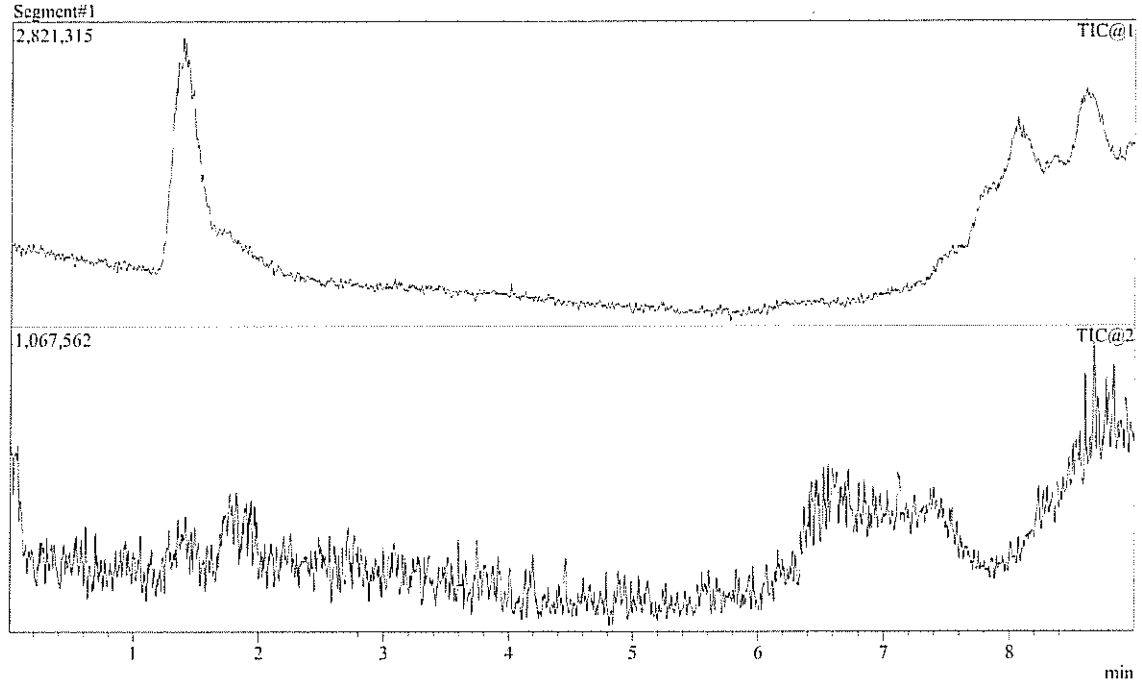

compound A

==== Shimadzu LabSolutions Data Report ====

<Spectrum>

Line#:1 R.Time:1.374(Scan#:1179)
MassPeaks:565
RawMode:Single 1.374(1179) BasePeak:152(1209166)
BG Mode:None Segment 1 - Event 1

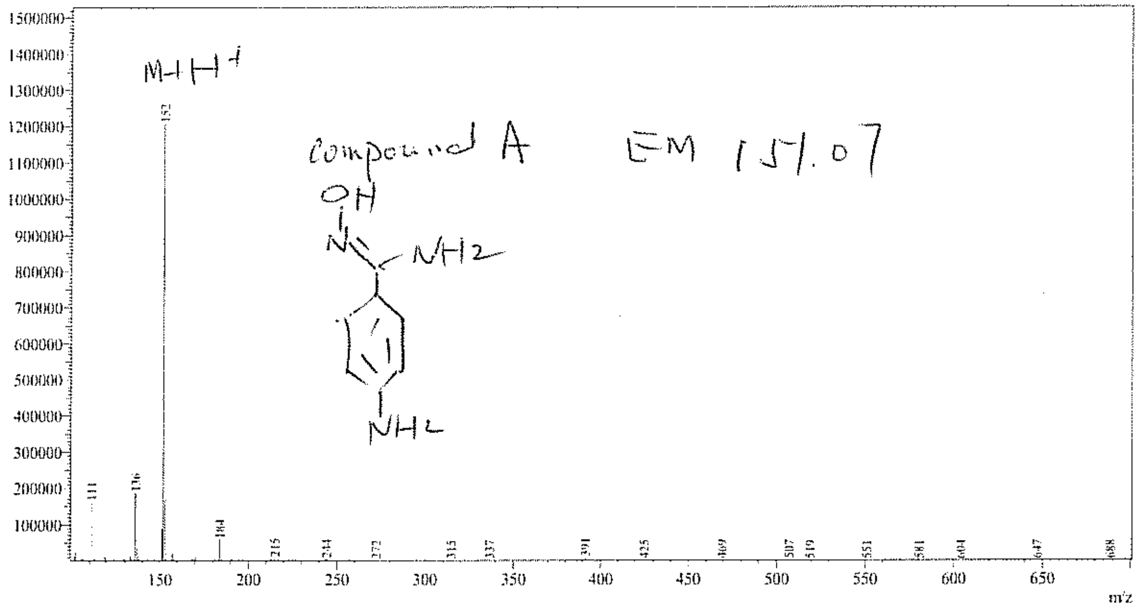

Line#:2 R.Time:1.375(Scan#:1180)
MassPeaks:635
RawMode:Single 1.375(1180) BasePeak:113(135896)
BG Mode:None Segment 1 - Event 2

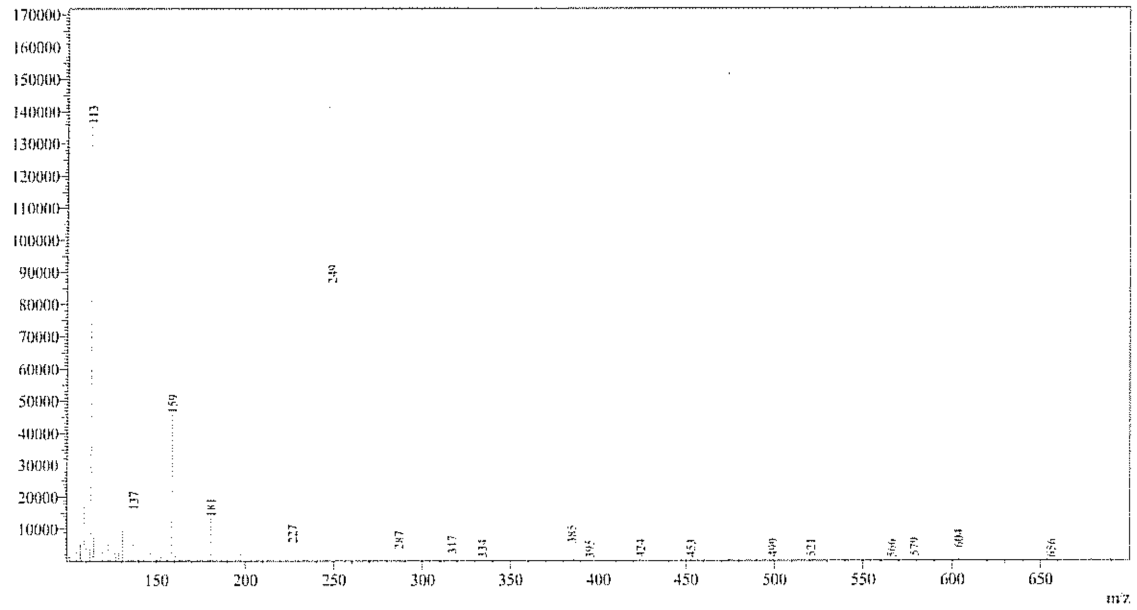

compound B Nov30-2015-yxh651.1.fid
Compound B for 117

^{1}H NMR (500 MHz, DMSO-d_6) δ 10.64 (s, 1H), 7.69 (s, 3H), 6.92 (s, 2H), 4.54 (s, 1H), 4.30 (s, 1H), 2.52 – 2.48 (m, 2H).

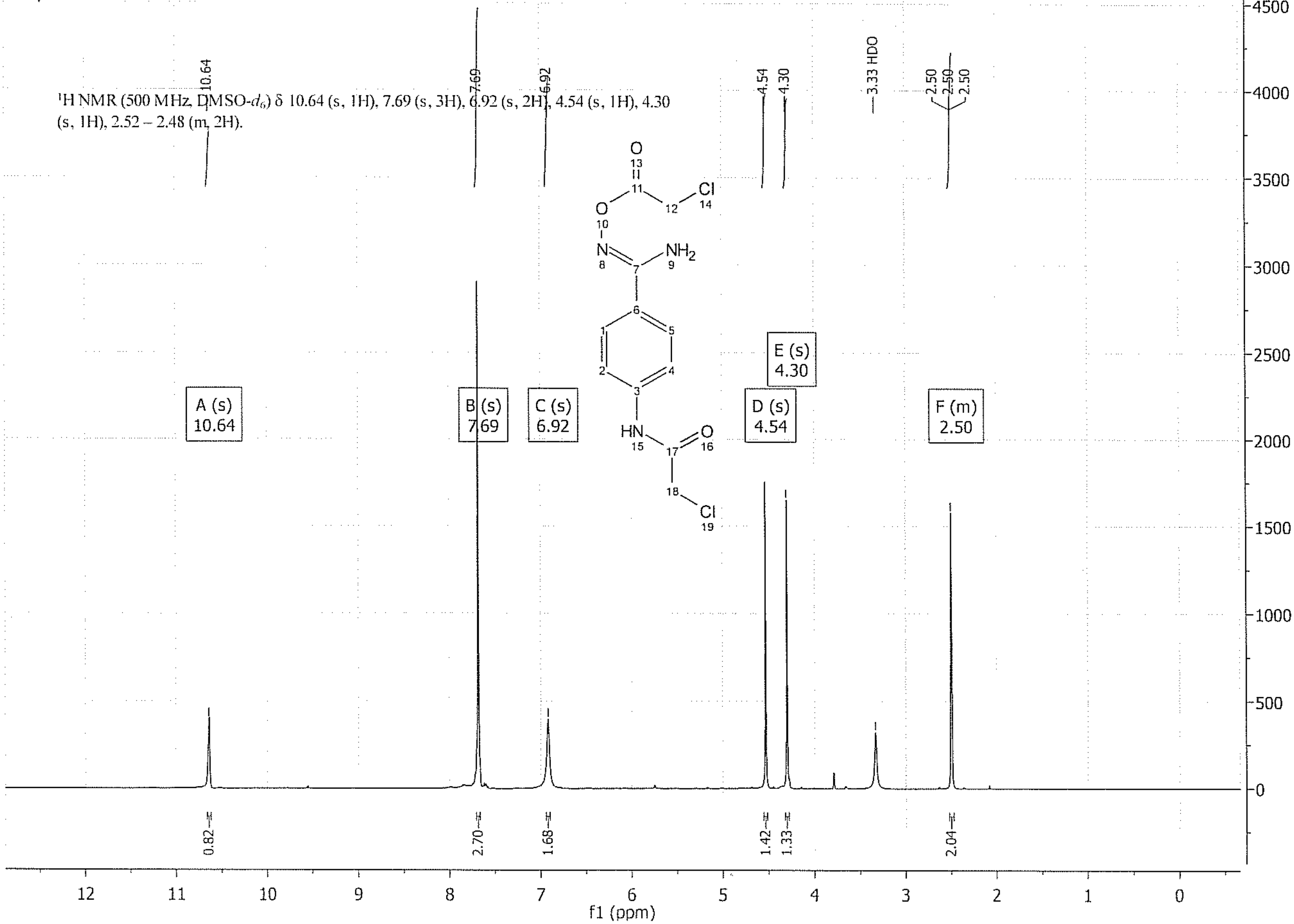

compound B Nov30-2015-yxh651.1.fid
Compound B for 117

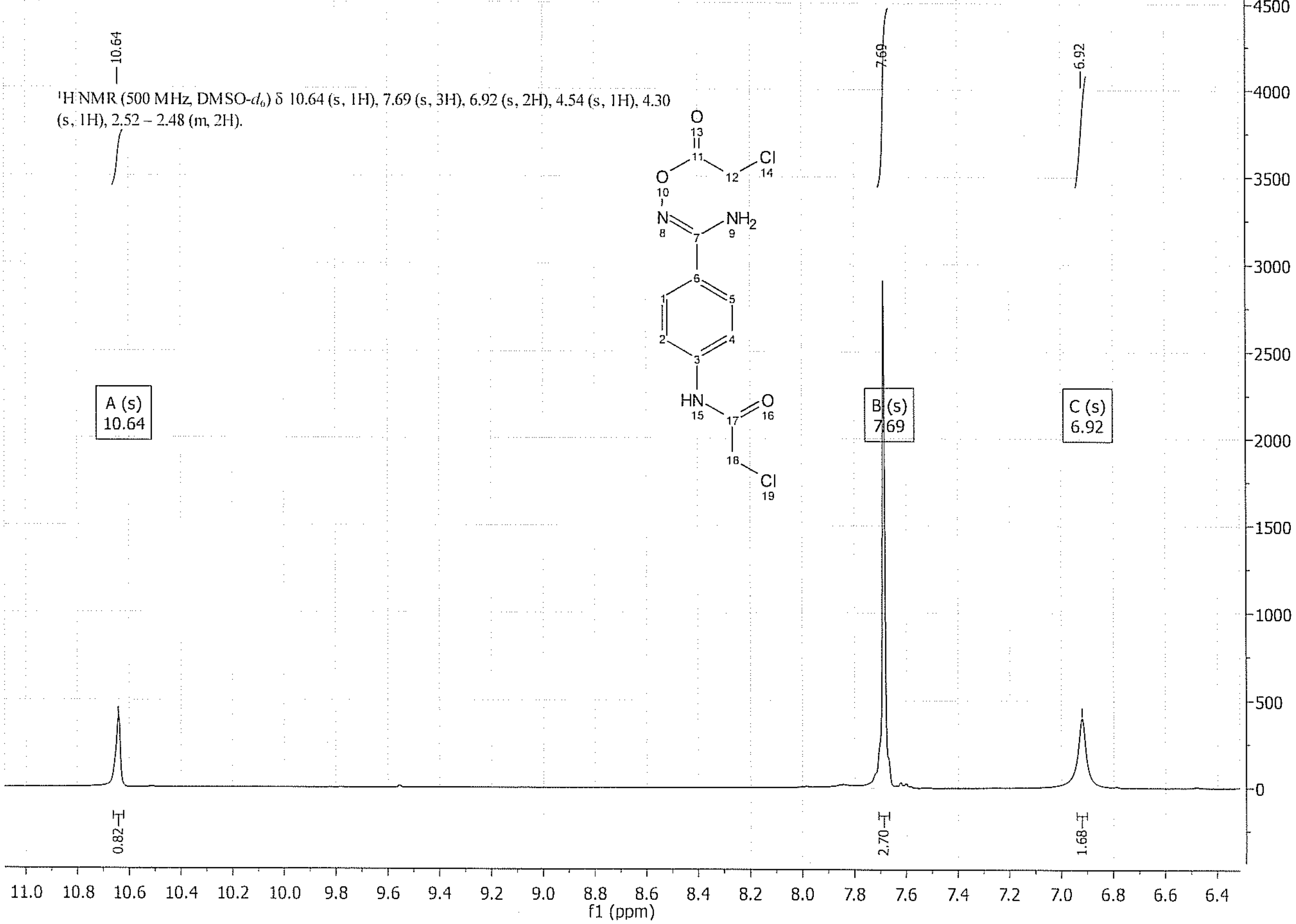

compound B Nov30-2015-yxh651.1.fid
Compound B for 117

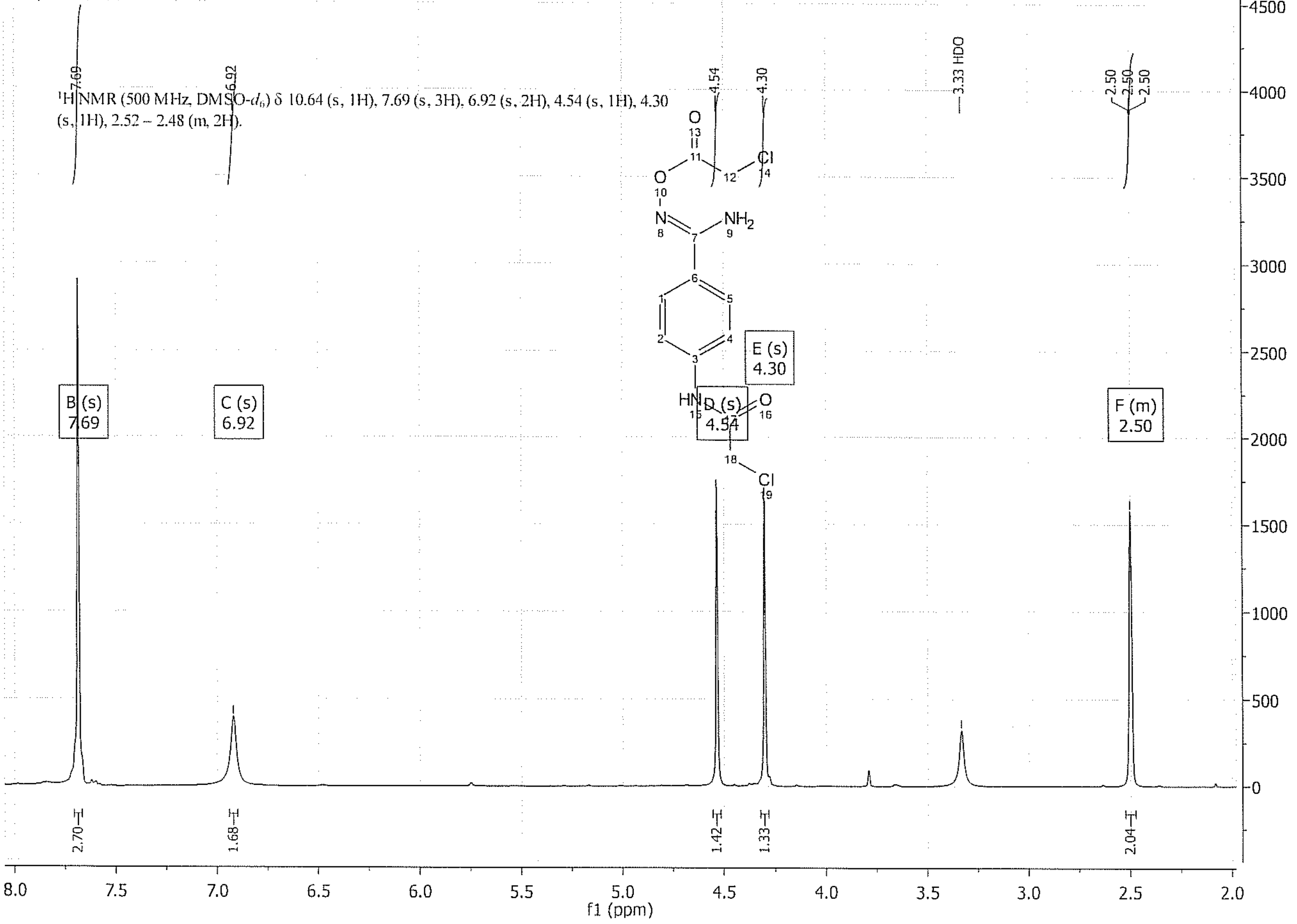

compound B Nov30-2015-yxh651.2.fid
Compound B for 117

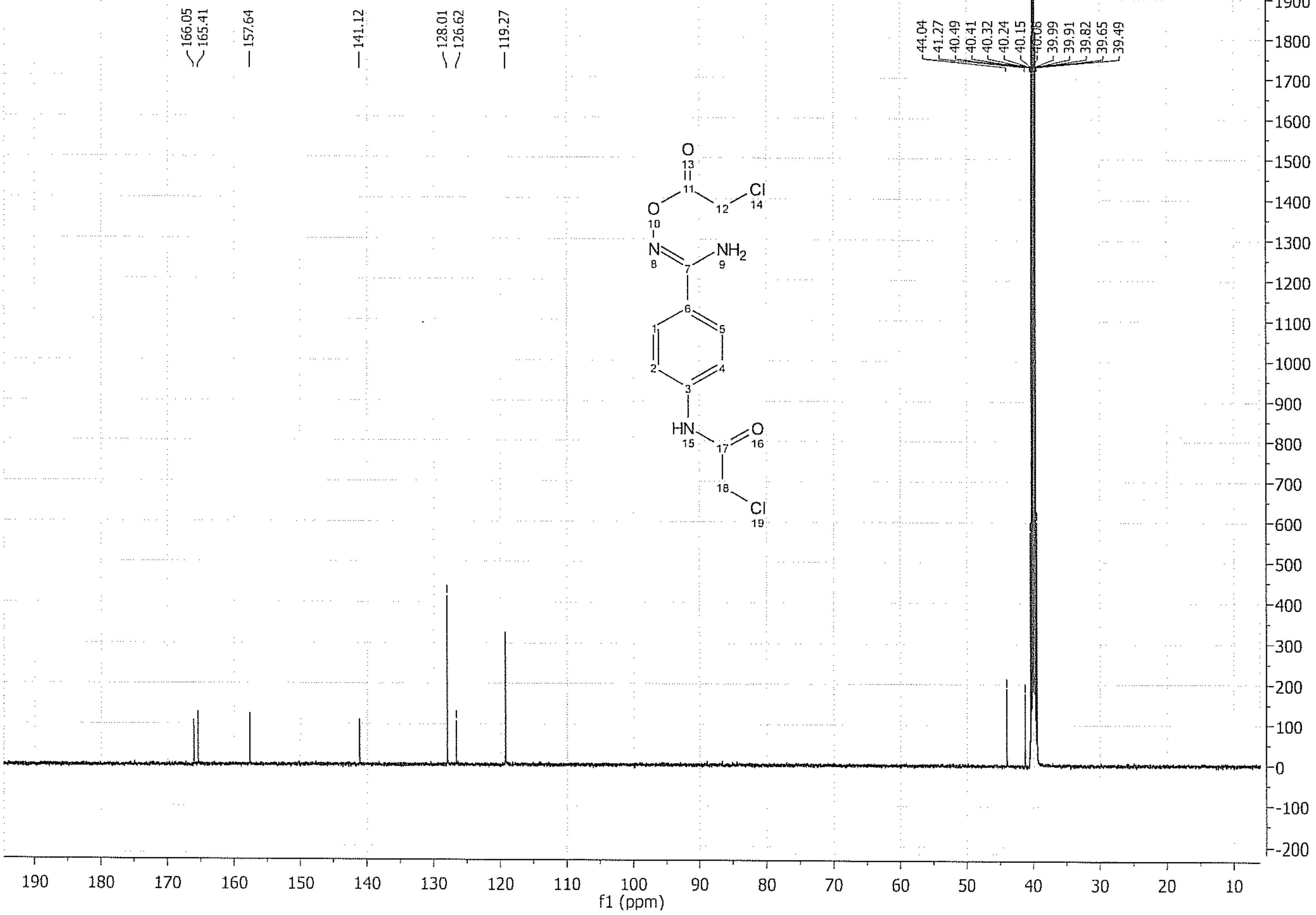

compound B Nov30-2015-yxh651.2.fid
Compound B for 117

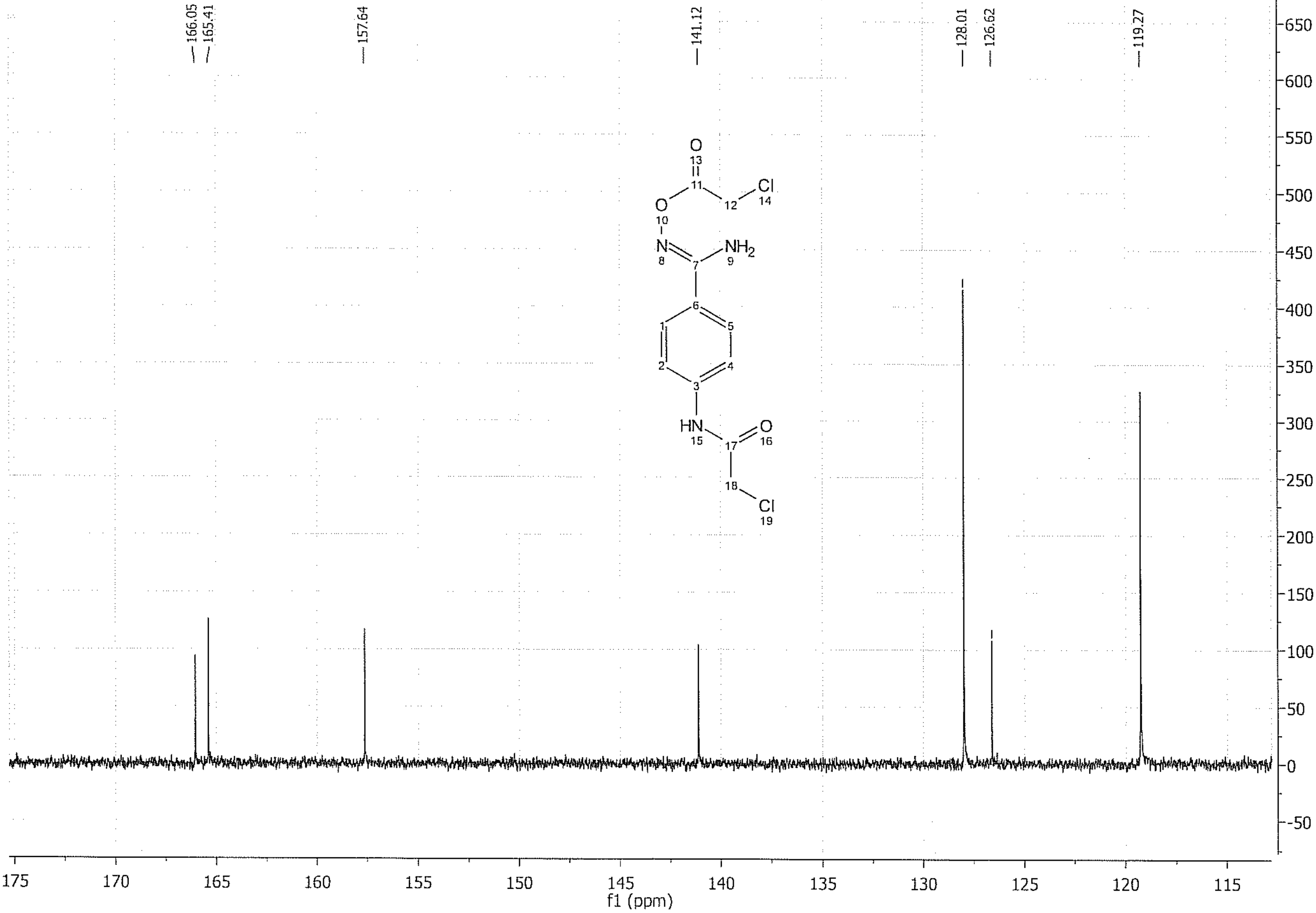

compound B Nov30-2015-yxh651.2.fid
Compound B for 117

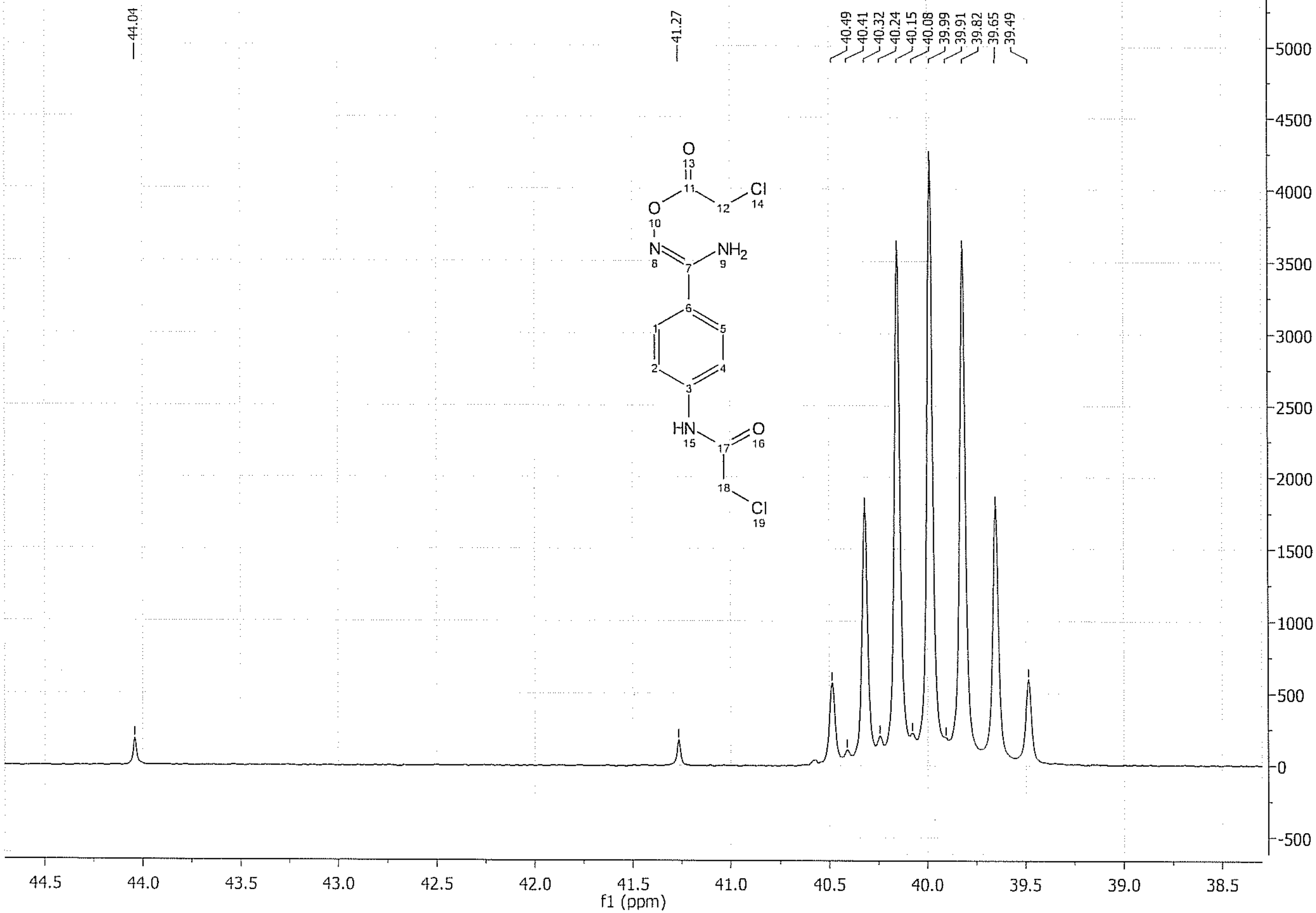

==== Shimadzu LabSolutions Data Report ====

<Chromatogram>

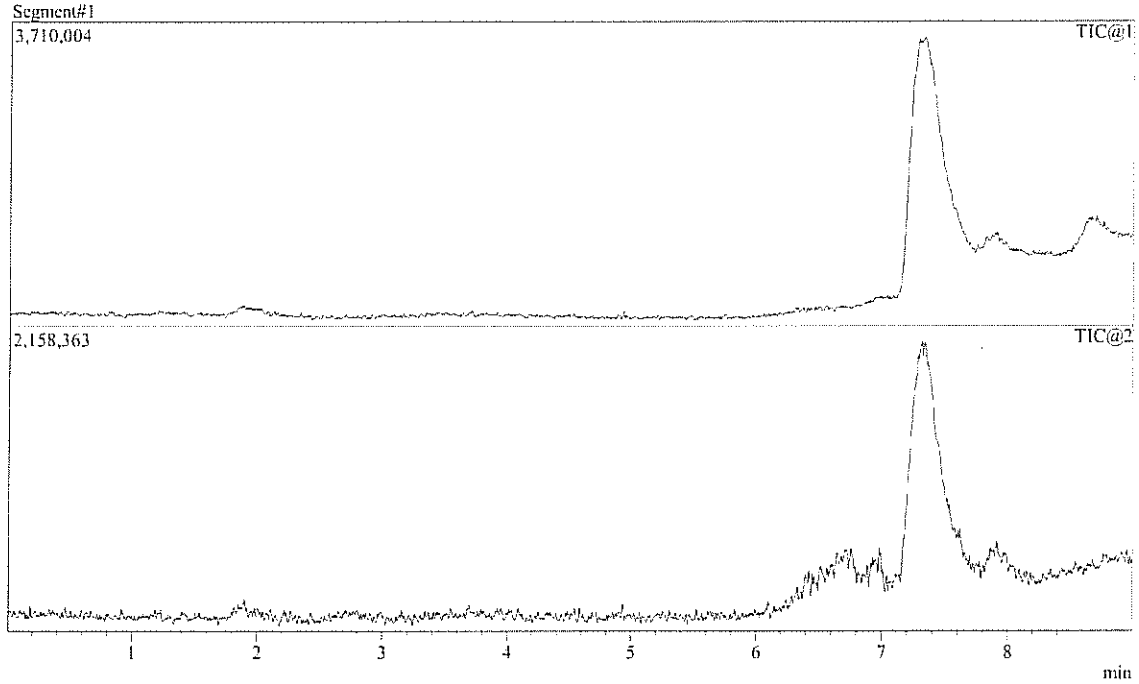

compound B

==== Shimadzu LabSolutions Data Report ====

<Spectrum>

Line#:1 R.Time:7.327(Scan#:6281)
MassPeaks:628
RawMode:Single 7.327(6281) BasePeak:304(440566)
BG Mode:None Segment 1 - Event 1

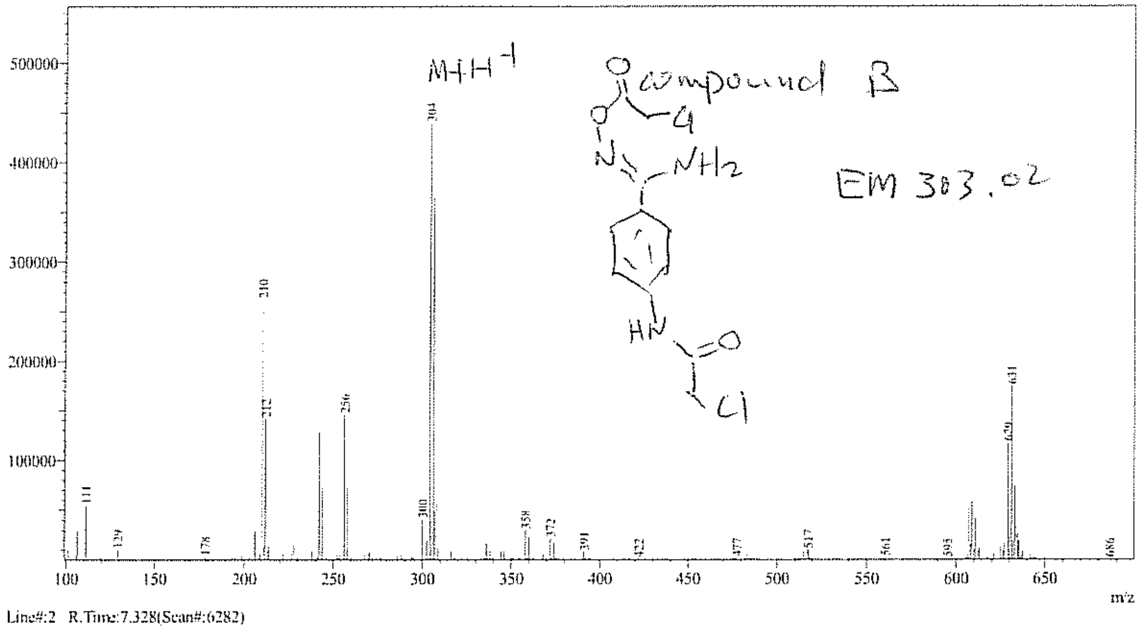

Line#:2 R.Time:7.328(Scan#:6282)
MassPeaks:629
RawMode:Single 7.328(6282) BasePeak:208(198708)
BG Mode:None Segment 1 - Event 2

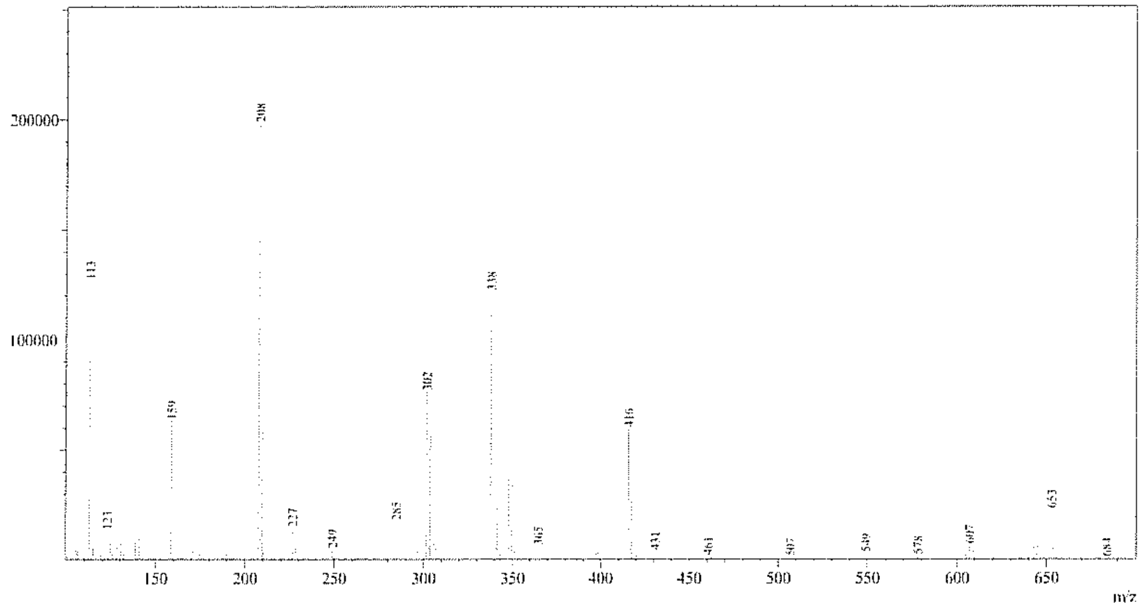

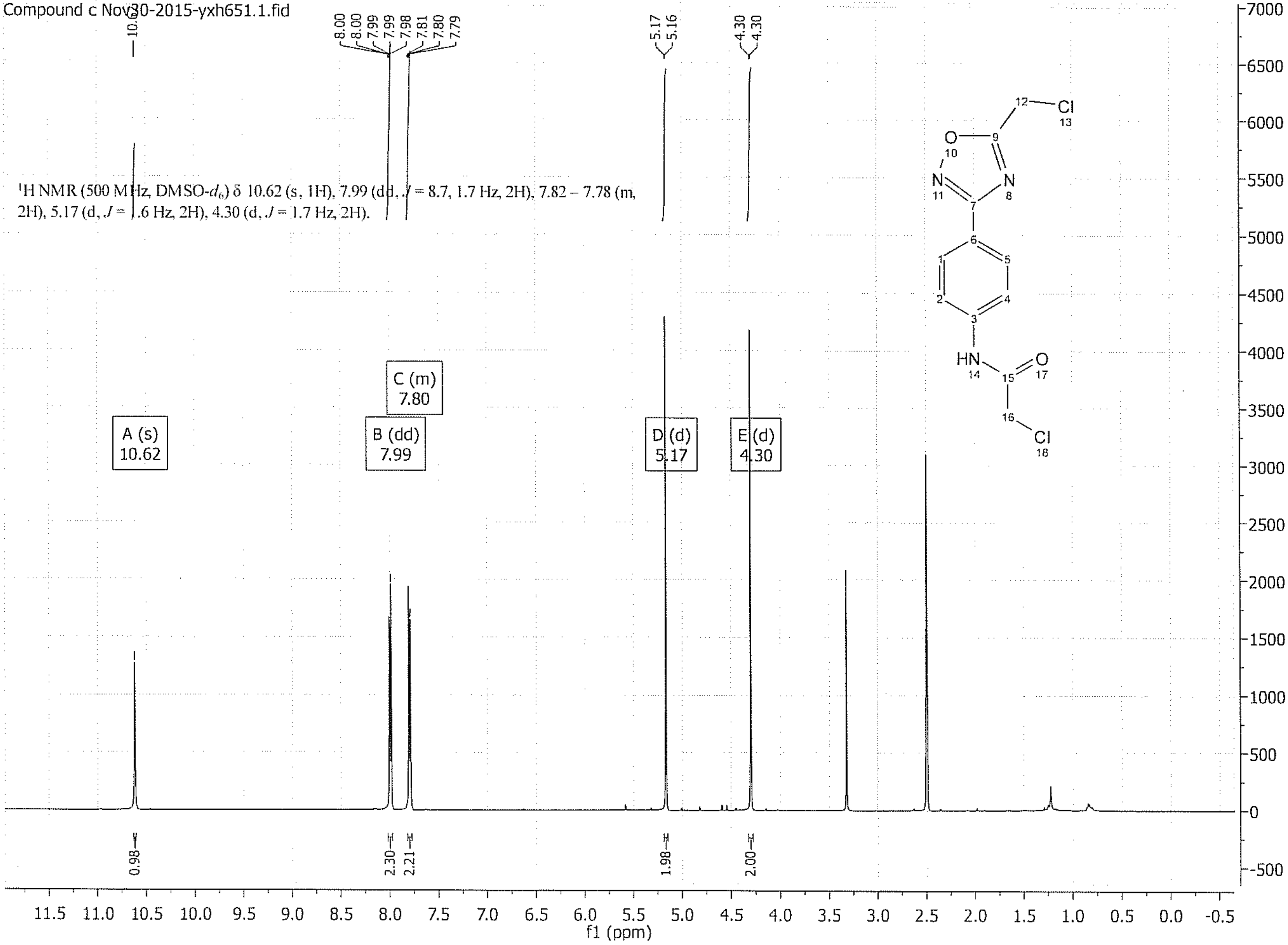
Compound c Nov30-2015-yxh651.1.fid
¹H NMR (500 MHz, DMSO-d_6) δ 10.62 (s, 1H), 7.99 (dd, J = 8.7, 1.7 Hz, 2H), 7.82 – 7.78 (m, 2H), 5.17 (d, J = 1.6 Hz, 2H), 4.30 (d, J = 1.7 Hz, 2H).
8.00 8.00 7.99 7.99 7.98 7.81 7.80 7.79
5.17 5.16
4.30 4.30
A (s) 10.62
B (dd) 7.99
C (m) 7.80
D (d) 5.17
E (d) 4.30
0.98
2.30
2.21
1.98
2.00
f1 (ppm)
HN
O
Cl
N
14 15 16 17 18
3 6 7 8 9 10 11 12 13
1 2 4 5

Compound c Nov30-2015-yxh651.1.fid

^{1}H NMR (500 MHz, DMSO-d_6) δ 10.62 (s, 1H), 7.99 (dd, J = 8.7, 1.7 Hz, 2H), 7.82 – 7.78 (m, 2H), 5.17 (d, J = 1.6 Hz, 2H), 4.30 (d, J = 1.7 Hz, 2H).

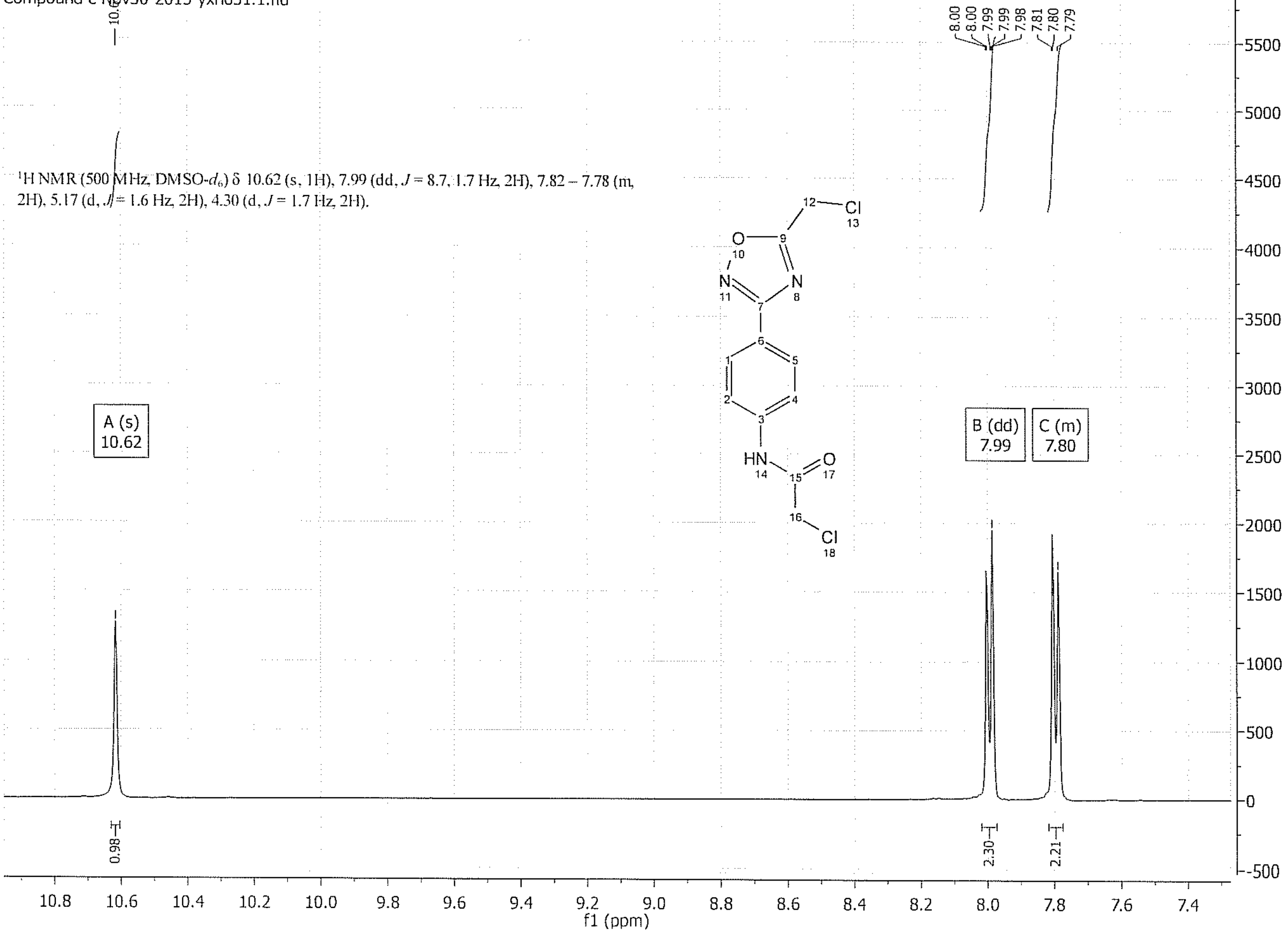

Compound c Nov30-2015-yxh651.1.fid

^{1}H NMR (500 MHz, DMSO-d_6) δ 10.62 (s, 1H), 7.99 (dd, J = 8.7, 1.7 Hz, 2H), 7.82 – 7.78 (m, 2H), 5.17 (d, J = 1.6 Hz, 2H), 4.30 (d, J = 1.7 Hz, 2H).

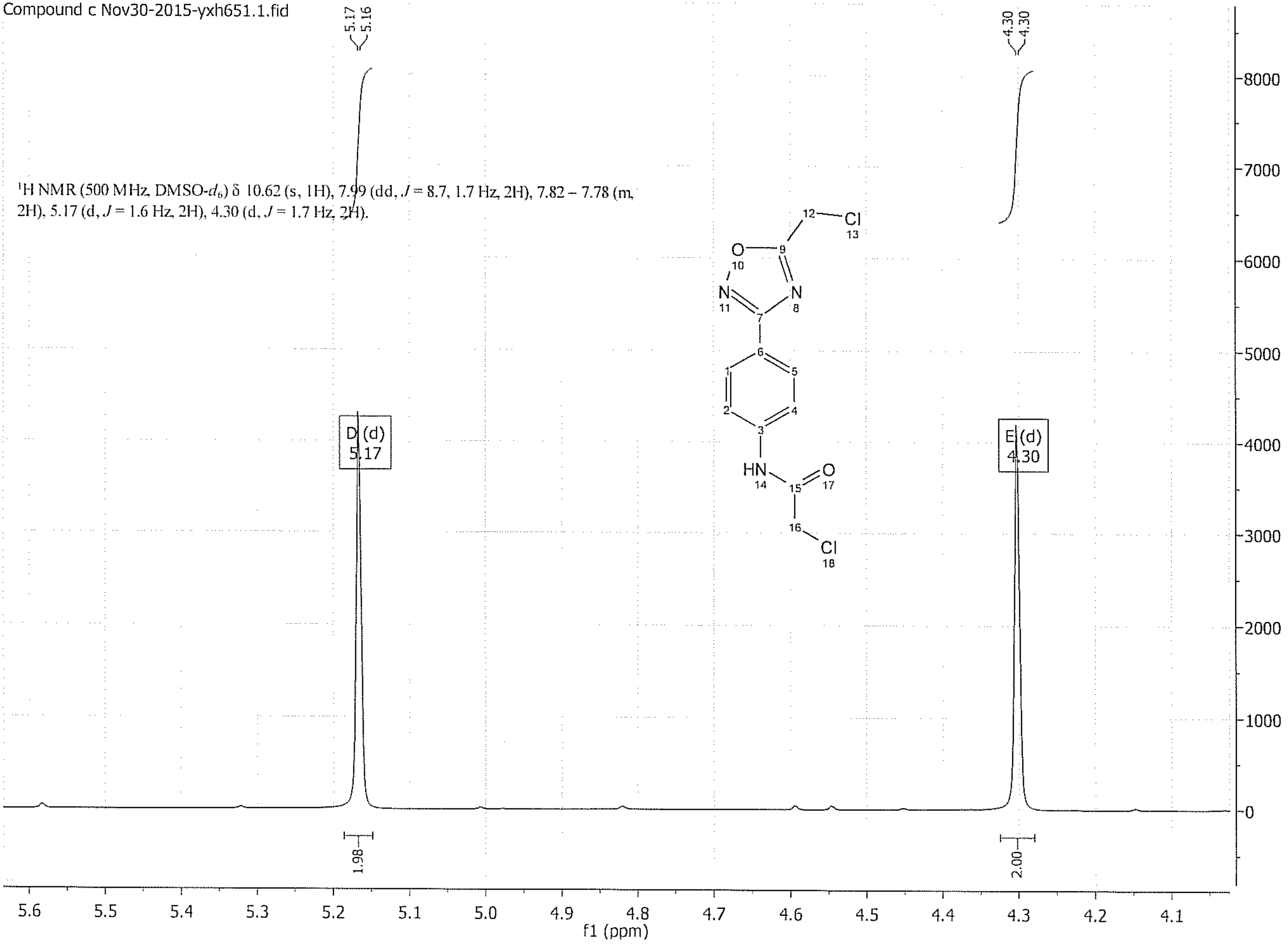

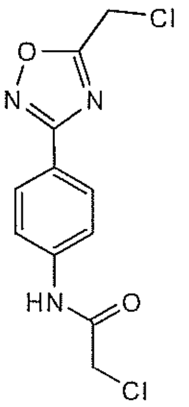

Cl
O
N
N
HN
O
Cl

Compound C

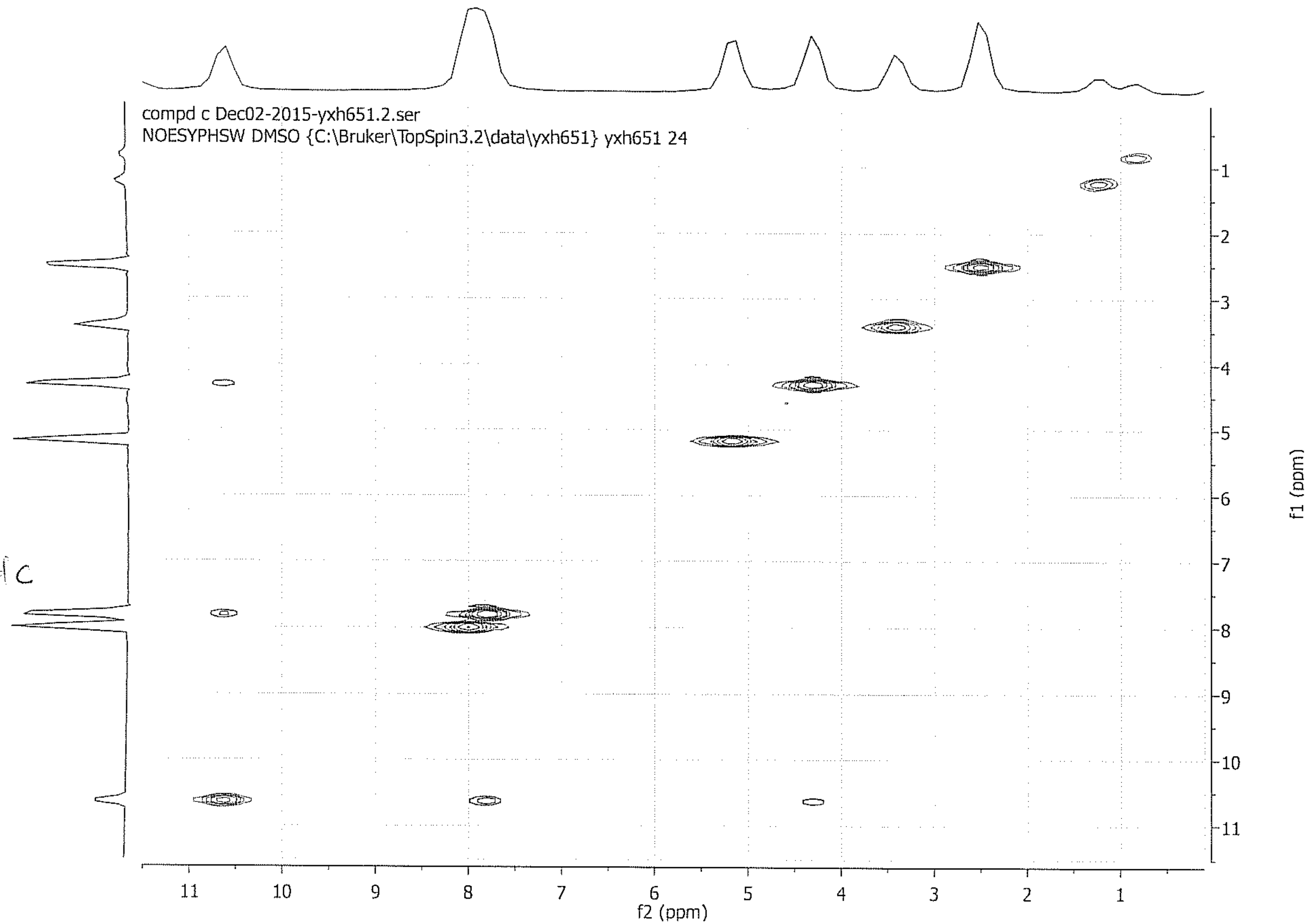

compd c Dec02-2015-yxh651.2.ser
NOESYPHSW DMSO {C:\Bruker\TopSpin3.2\data\yxh651} yxh651 24
f2 (ppm)
f1 (ppm)

Compound c Nov30-2015-yxh651.2.fid
compound c for 117

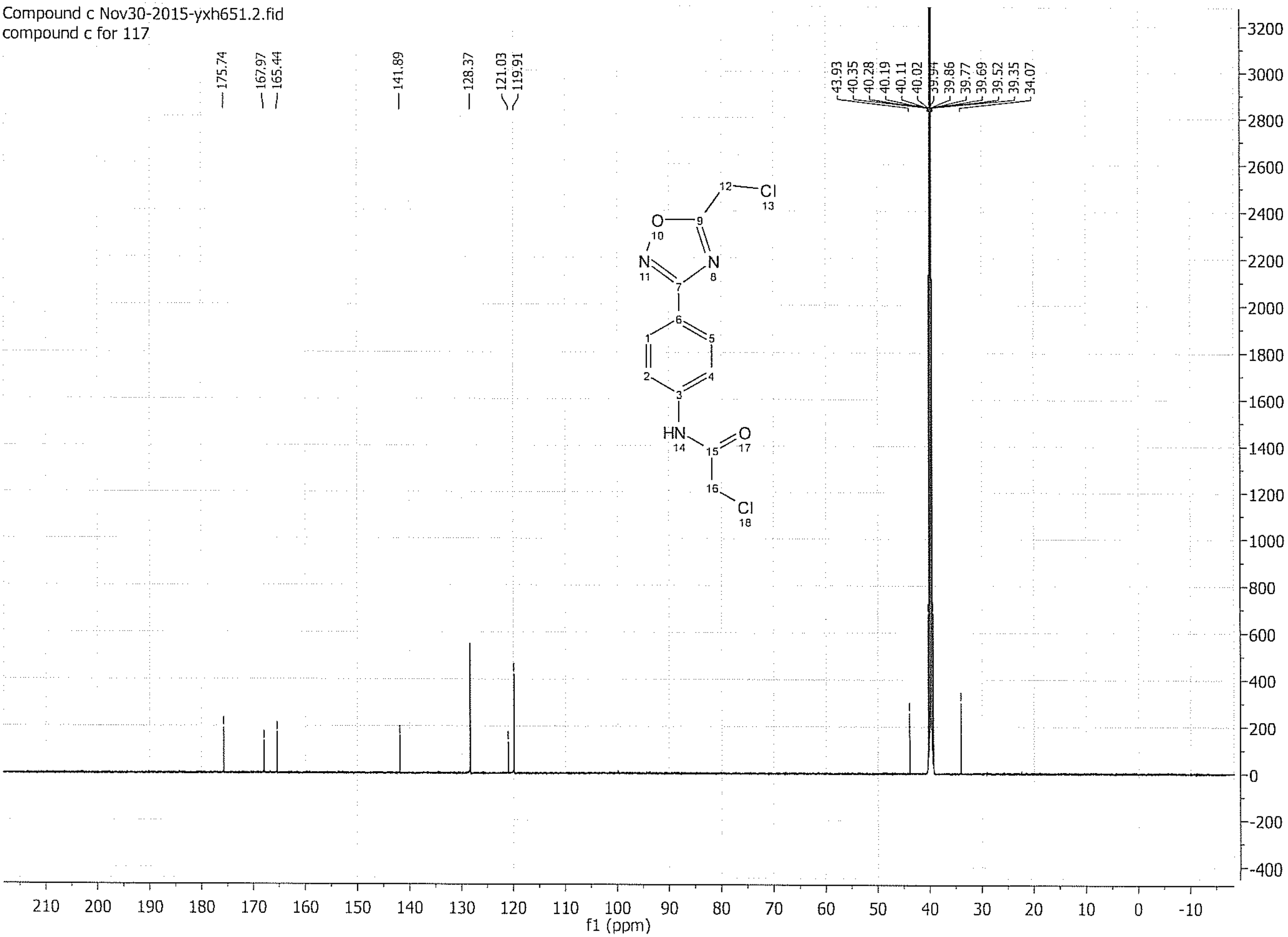

Compound c Nov30-2015-yxh651.2.fid
compound c for 117

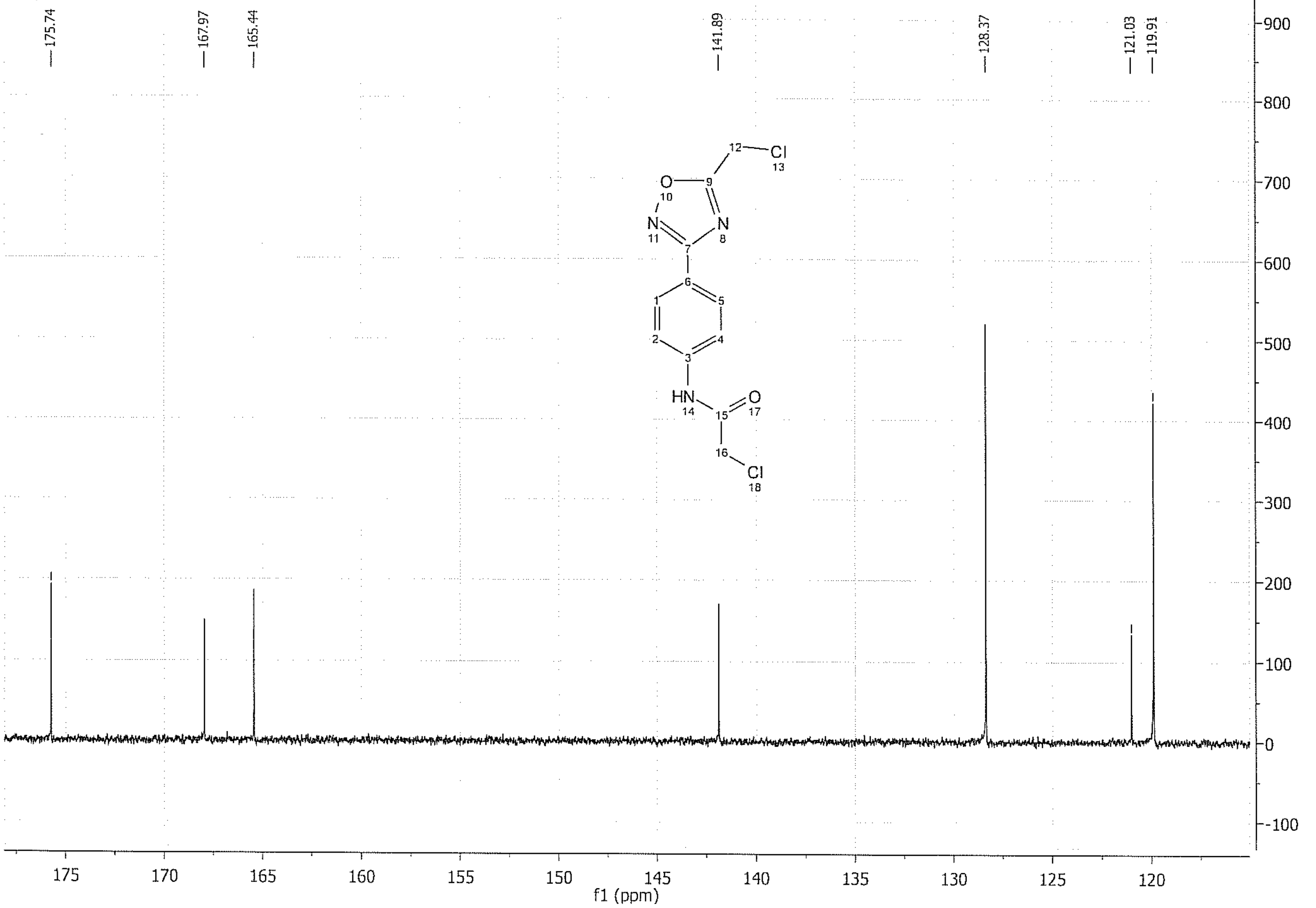

Compound c Nov30-2015-yxh651.2.fid
compound c for 117

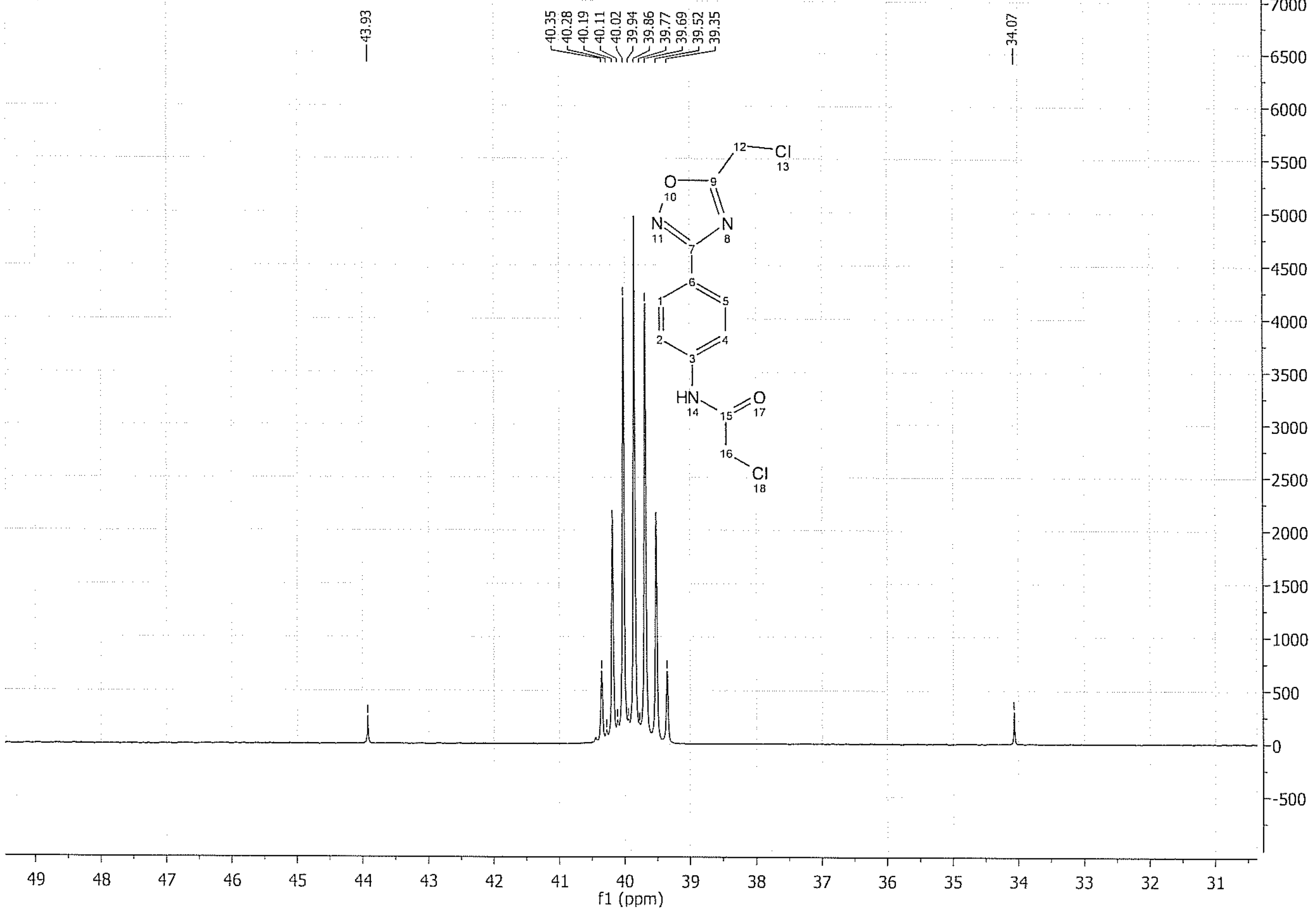

==== Shimadzu LabSolutions Data Report ====

<Chromatogram>

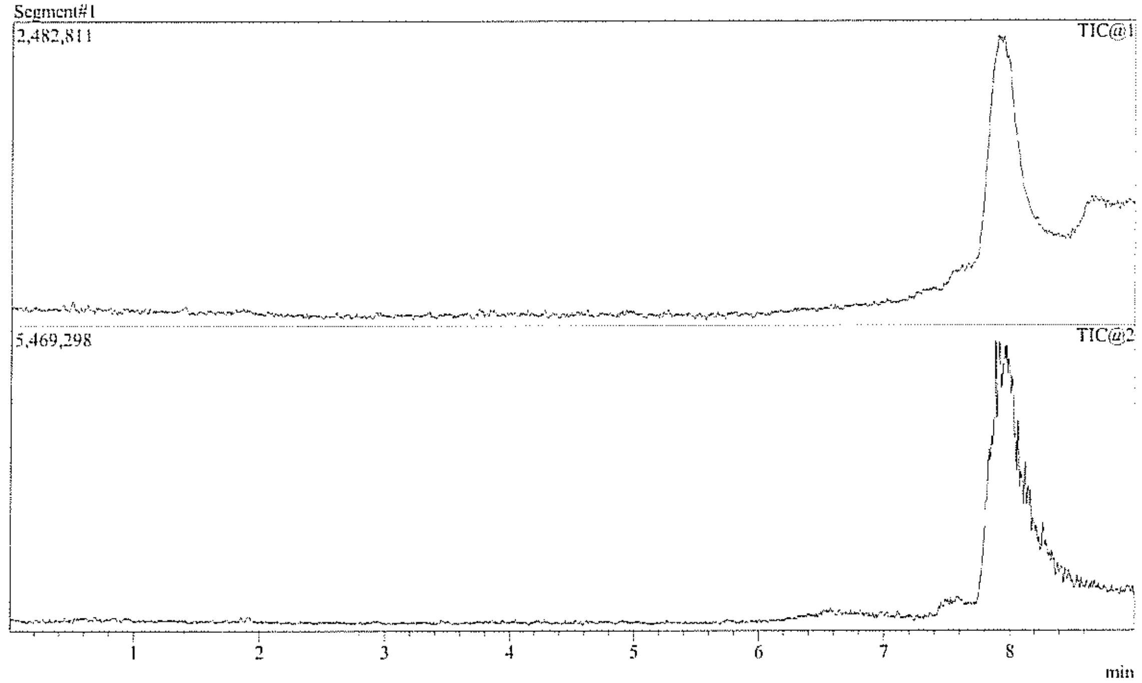

Compound C

==== Shimadzu LabSolutions Data Report ====

<Spectrum>

Line#:1 R.Time:7.933(Scan#:6801)
MassPeaks:642
RawMode:Single 7.933(6801) BasePeak:318(186799)
BG Mode:None Segment 1 - Event 1

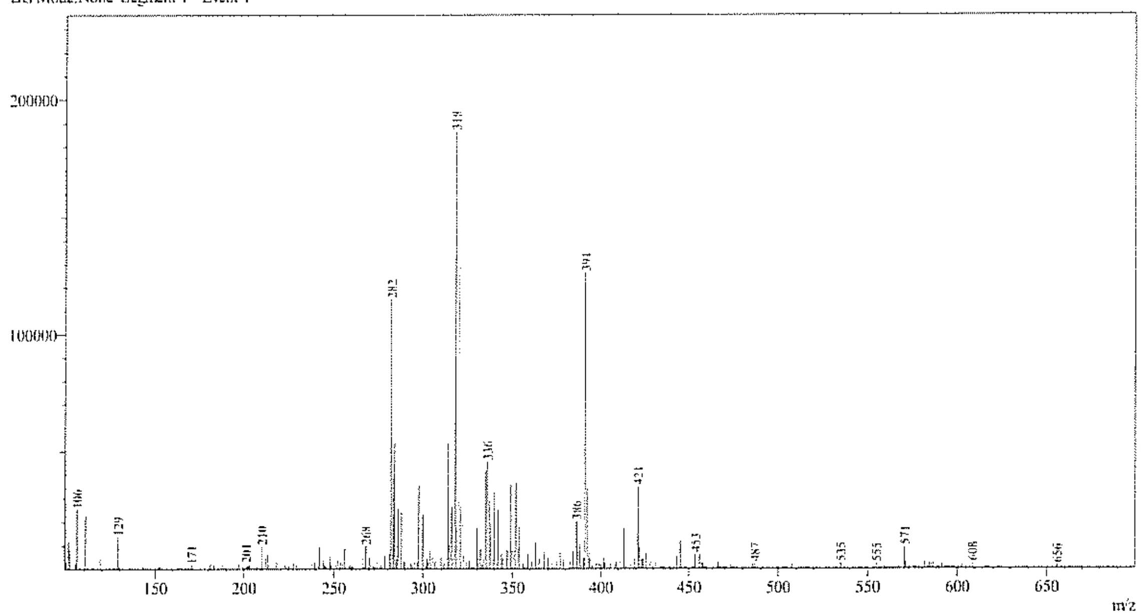

Line#:2 R.Time:7.934(Scan#:6802)
MassPeaks:623
RawMode:Single 7.934(6802) BasePeak:284(527255)
BG Mode:None Segment 1 - Event 2

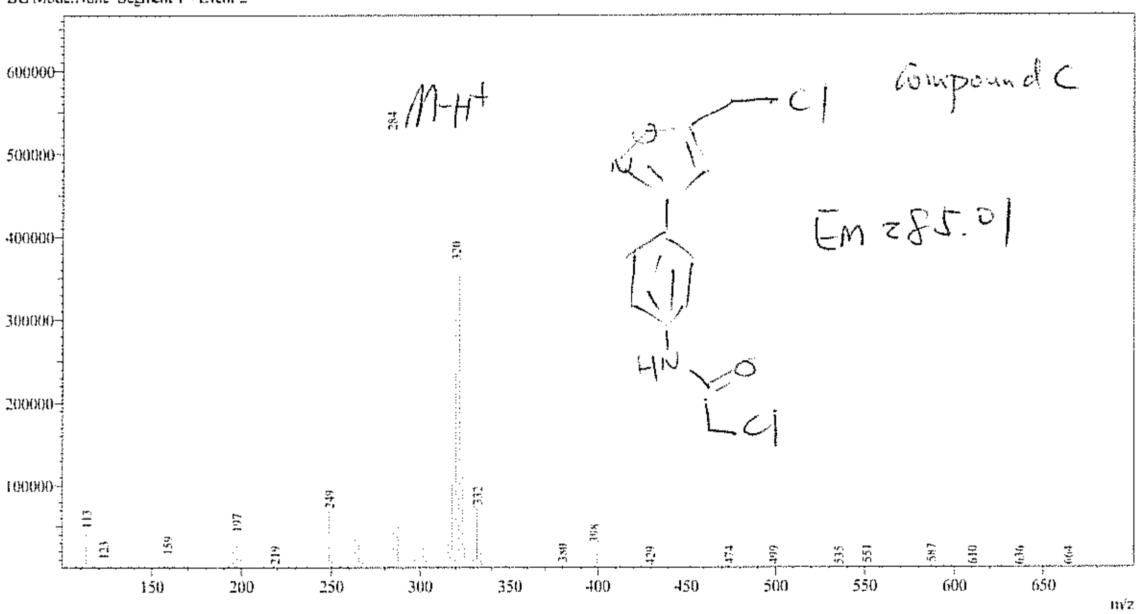

Compound D Nov30-2015-yxh651.1.fid

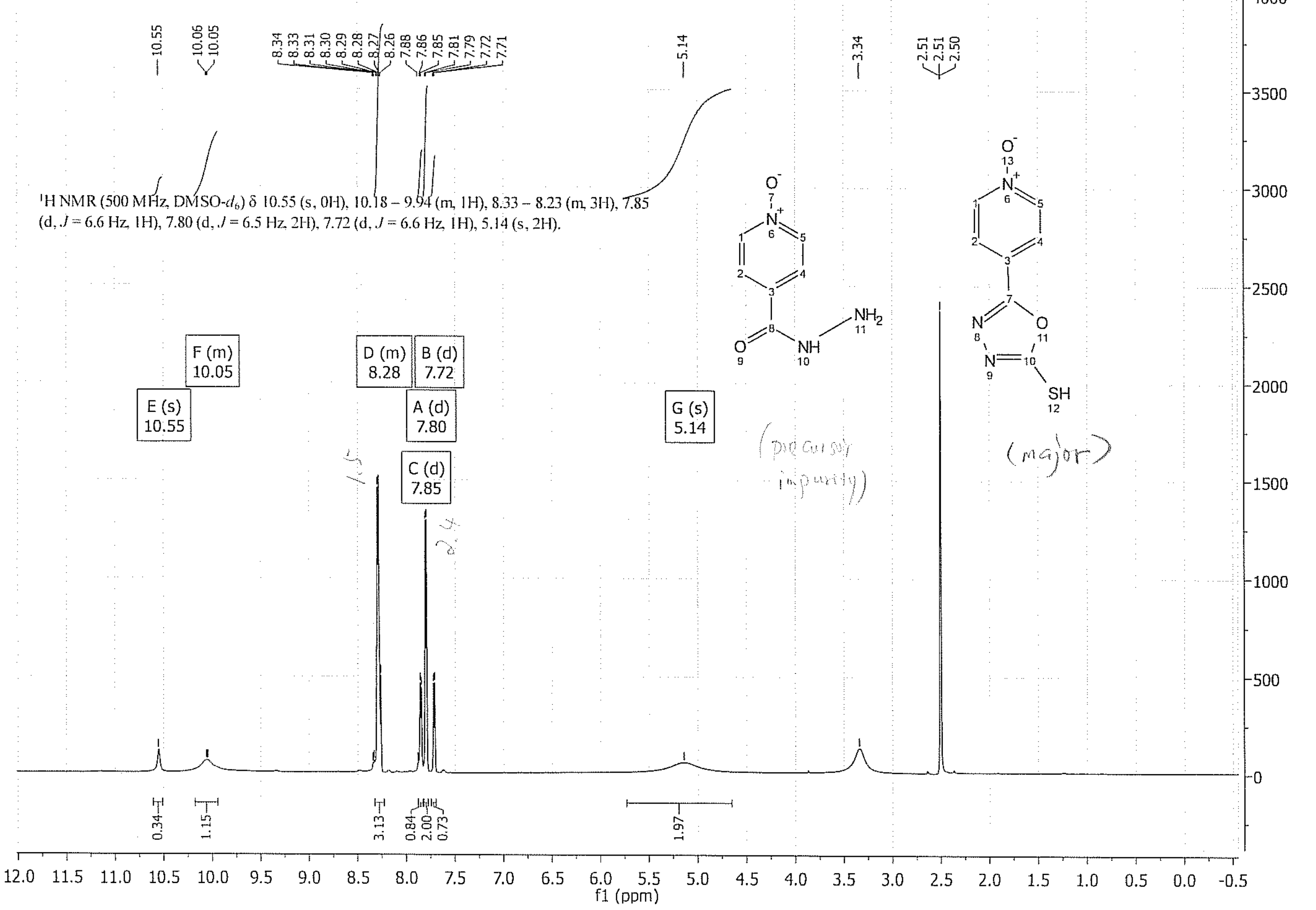

Compound D Nov30-2015-yxh651.1.fid

^{1}H NMR (500 MHz, DMSO-d_6) δ 10.55 (s, 0H), 10.18 – 9.94 (m, 1H), 8.33 – 8.23 (m, 3H), 7.85 (d, J = 6.6 Hz, 1H), 7.80 (d, J = 6.5 Hz, 2H), 7.72 (d, J = 6.6 Hz, 1H), 5.14 (s, 2H).

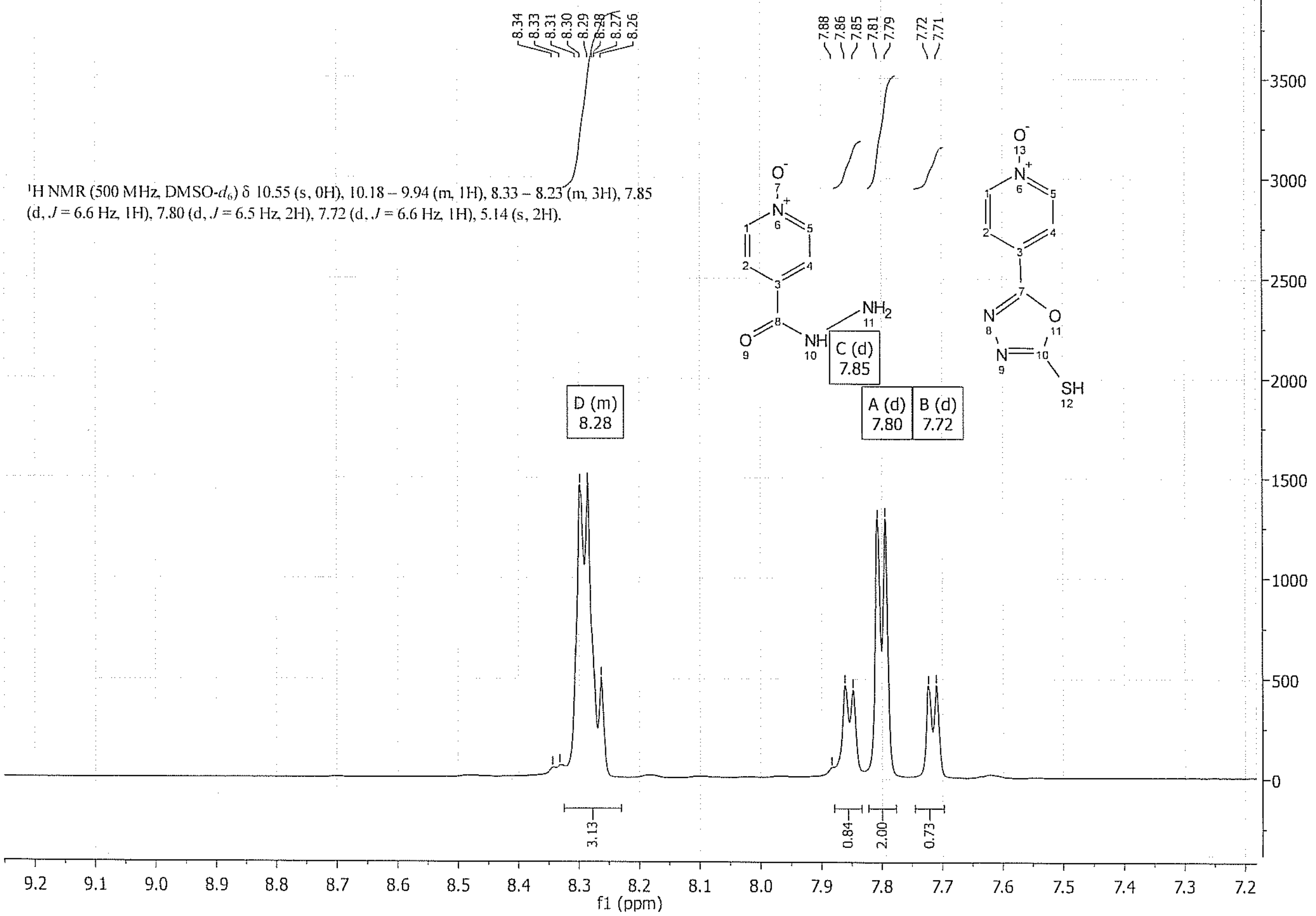

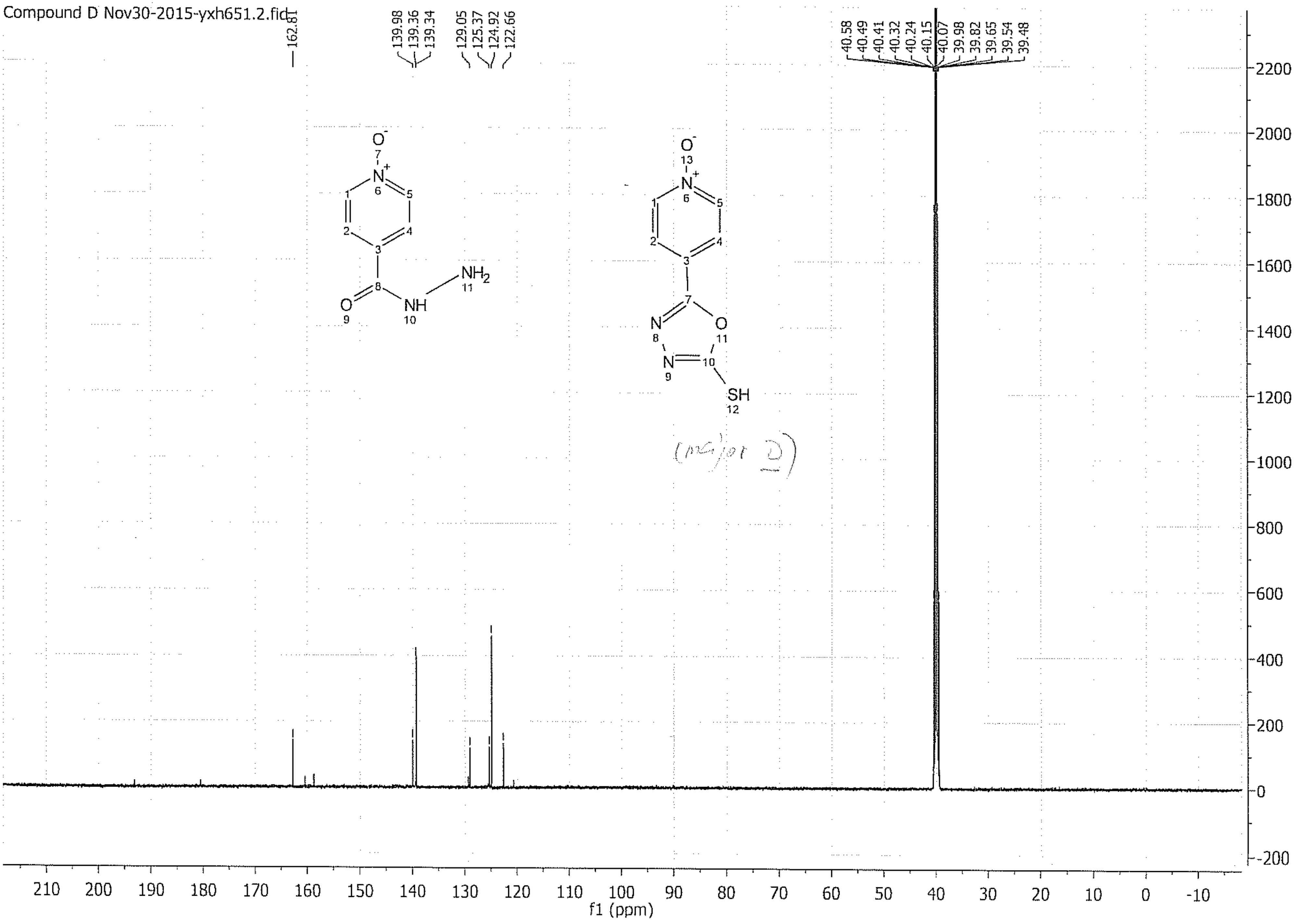

Compound D Nov30-2015-yxh651.2.fid
162.81
139.98
139.36
139.34
129.05
125.37
124.92
122.66
40.58
40.49
40.41
40.32
40.24
40.15
40.07
39.98
39.82
39.65
39.54
39.48
f1 (ppm)

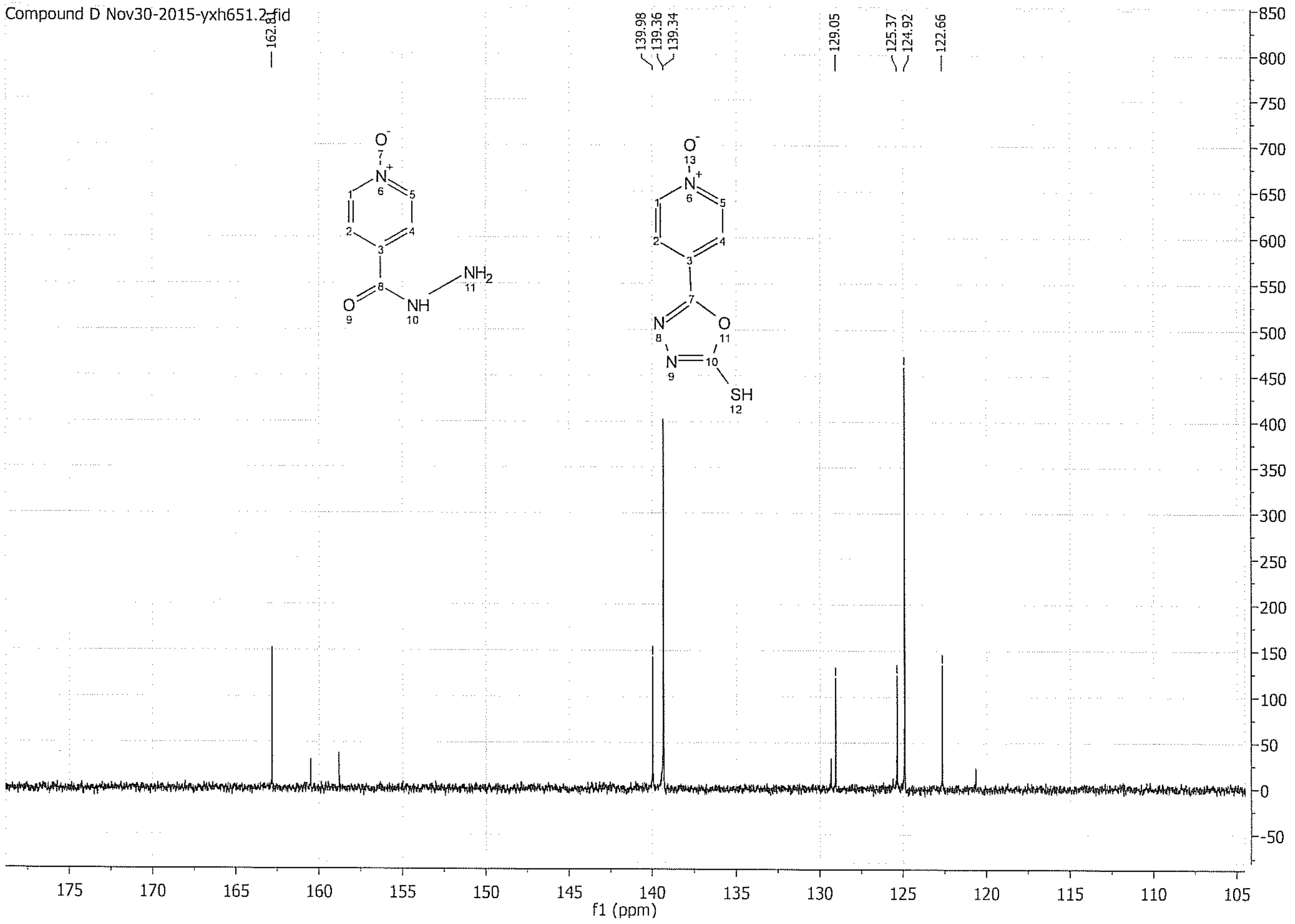

Compound D Nov30-2015-yxh651.2.fid
162.8
139.98
139.36
139.34
129.05
125.37
124.92
122.66
O-
N+
NH2
O
NH
SH
f1 (ppm)

==== Shimadzu LabSolutions Data Report ====

<Chromatogram>

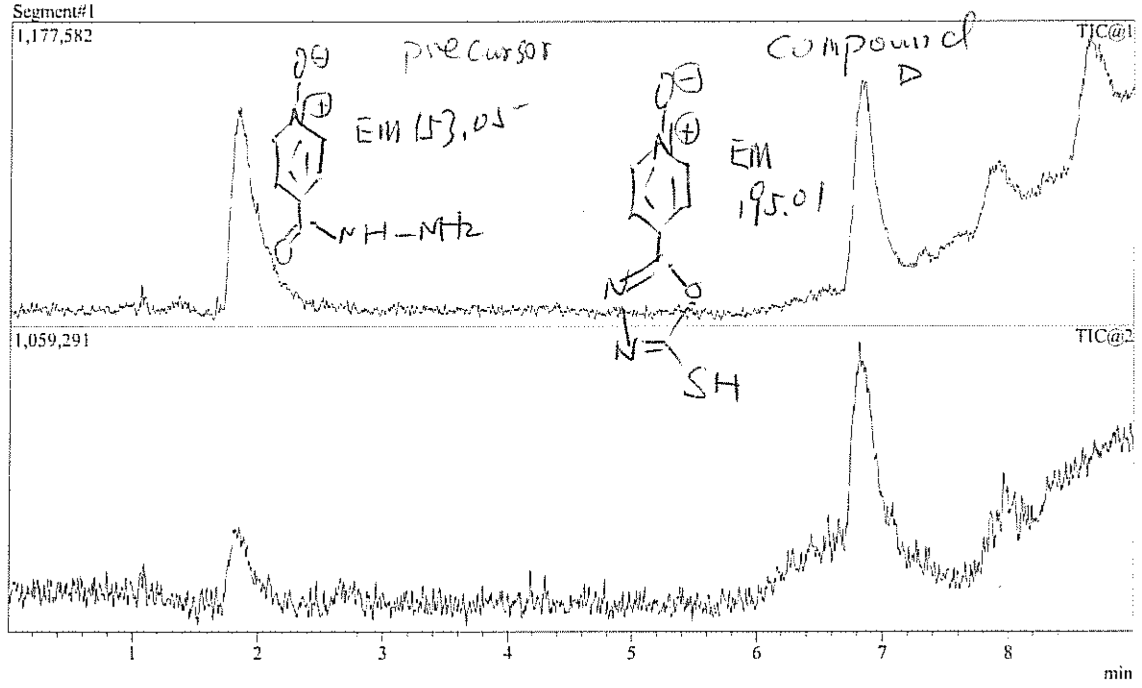

==== Shimadzu LabSolutions Data Report ====

<Spectrum>

Line#:1 R.Time:6.855(Scan#:5877)
MassPeaks:540
RawMode:Single 6.855(5877) BasePeak:196(161723)
BG Mode:None Segment 1 - Event 1

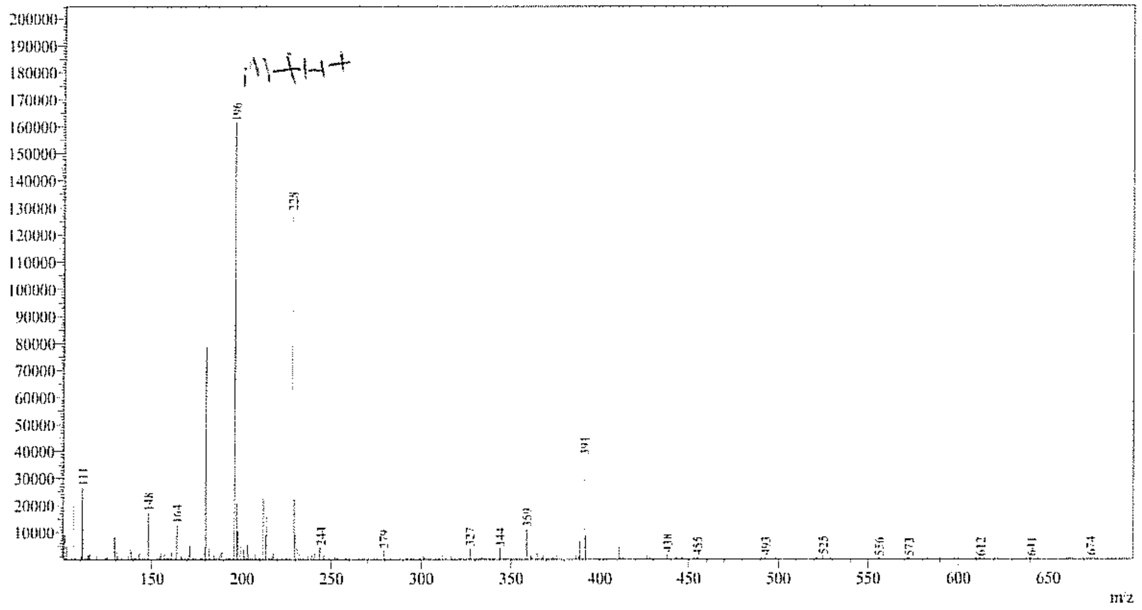

Line#:2 R.Time:6.856(Scan#:5878)
MassPeaks:622
RawMode:Single 6.856(5878) BasePeak:194(229198)
BG Mode:None Segment 1 - Event 2

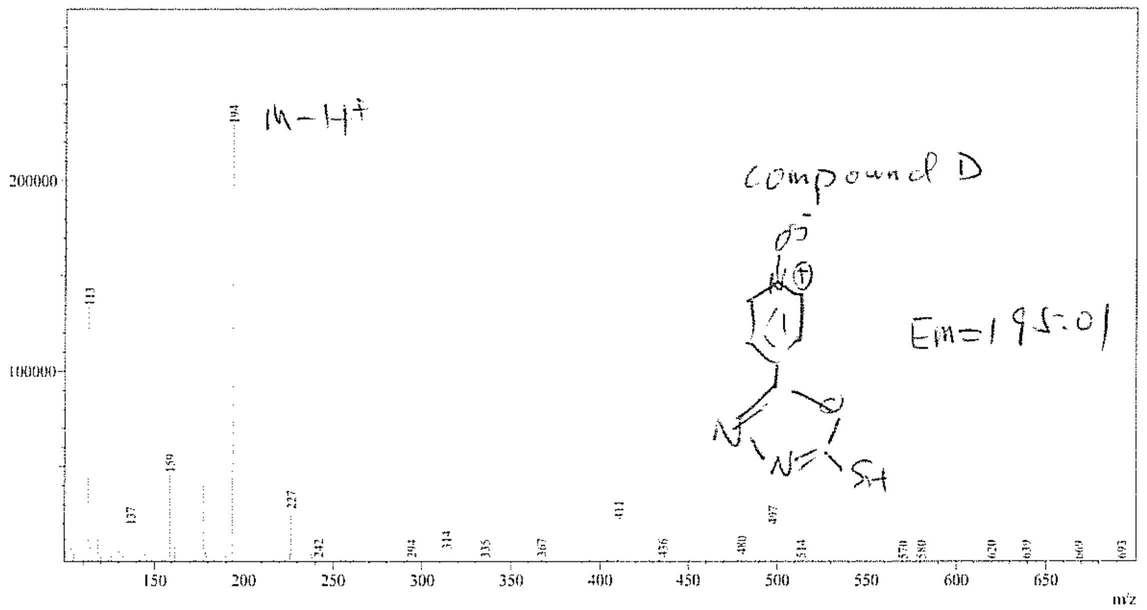

==== Shimadzu LabSolutions Data Report ====

<Spectrum>

Line#:1 R.Time:1.878(Scan#:1611)
MassPeaks:503
RawMode:Single 1.878(1611) BasePeak:154(169259)
BG Mode:None Segment 1 - Event 1

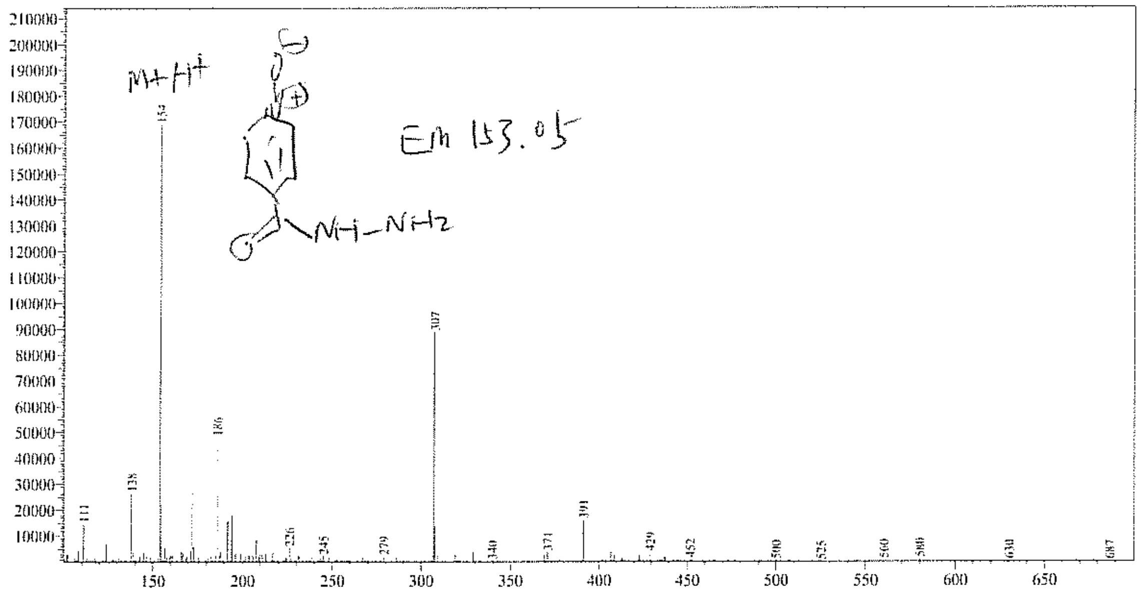

Line#:2 R.Time:1.879(Scan#:1612)
MassPeaks:627
RawMode:Single 1.879(1612) BasePeak:113(103077)
BG Mode:None Segment 1 - Event 2

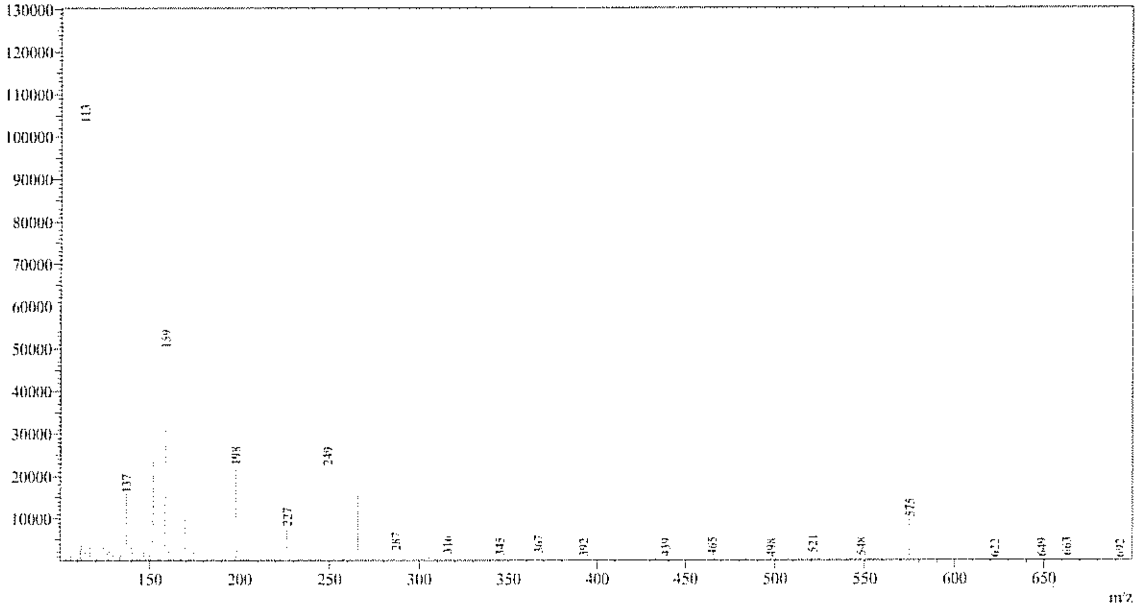

Compound 117-2-Nov30-2015-yxh651.1.fid

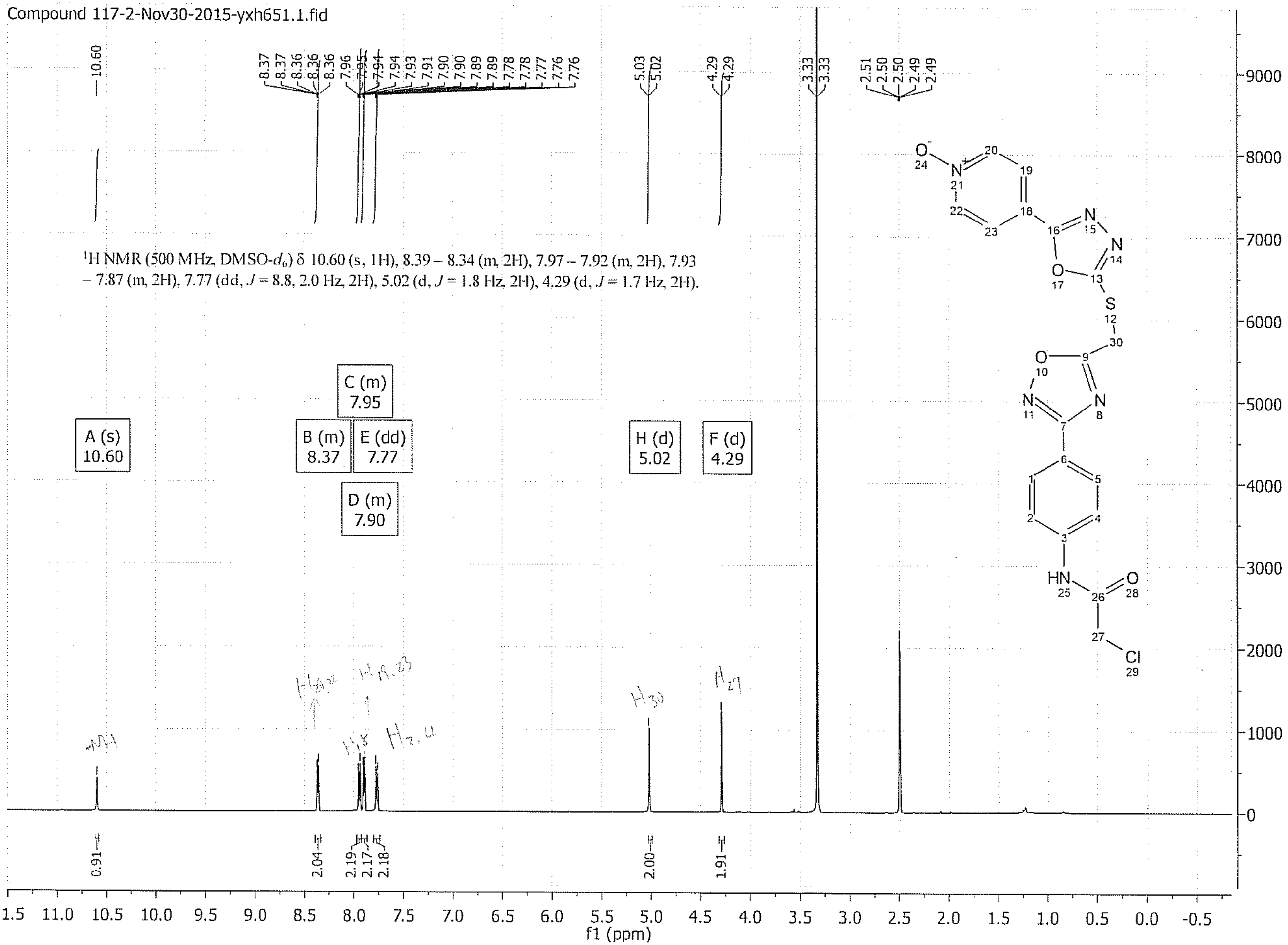

^{1}H NMR (500 MHz, DMSO-d_6) δ 10.60 (s, 1H), 8.39 – 8.34 (m, 2H), 7.97 – 7.92 (m, 2H), 7.93 – 7.87 (m, 2H), 7.77 (dd, J = 8.8, 2.0 Hz, 2H), 5.02 (d, J = 1.8 Hz, 2H), 4.29 (d, J = 1.7 Hz, 2H).

Compound 117-2-Nov30-2015-yxh651.1.fid

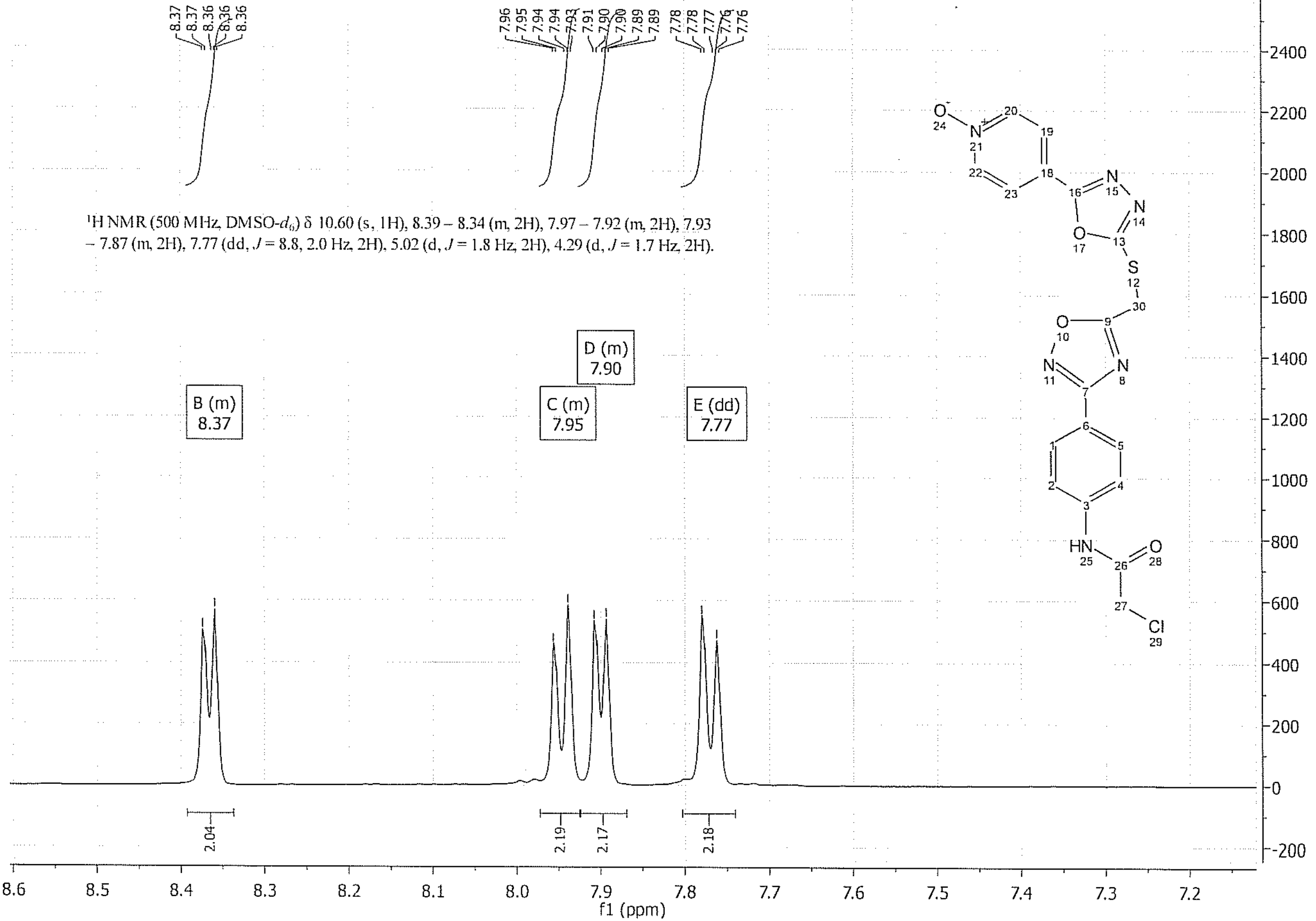

^{1}H NMR (500 MHz, DMSO-d_6) δ 10.60 (s, 1H), 8.39 – 8.34 (m, 2H), 7.97 – 7.92 (m, 2H), 7.93 – 7.87 (m, 2H), 7.77 (dd, J = 8.8, 2.0 Hz, 2H), 5.02 (d, J = 1.8 Hz, 2H), 4.29 (d, J = 1.7 Hz, 2H).

Compound 117-2-Nov30-2015-yxh651.1.fid

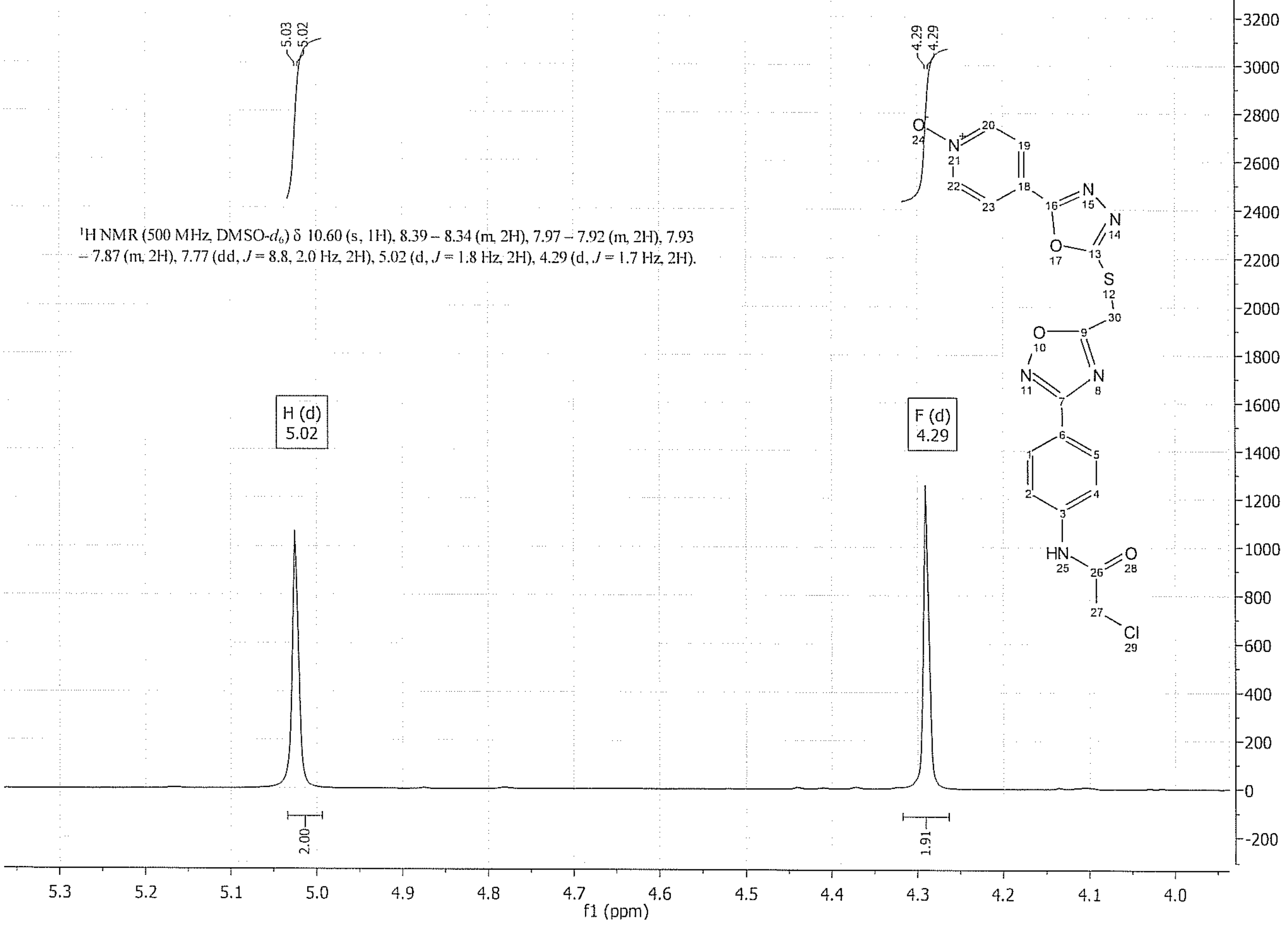

^{1}H NMR (500 MHz, DMSO-d_6) δ 10.60 (s, 1H), 8.39 – 8.34 (m, 2H), 7.97 – 7.92 (m, 2H), 7.93 – 7.87 (m, 2H), 7.77 (dd, J = 8.8, 2.0 Hz, 2H), 5.02 (d, J = 1.8 Hz, 2H), 4.29 (d, J = 1.7 Hz, 2H).

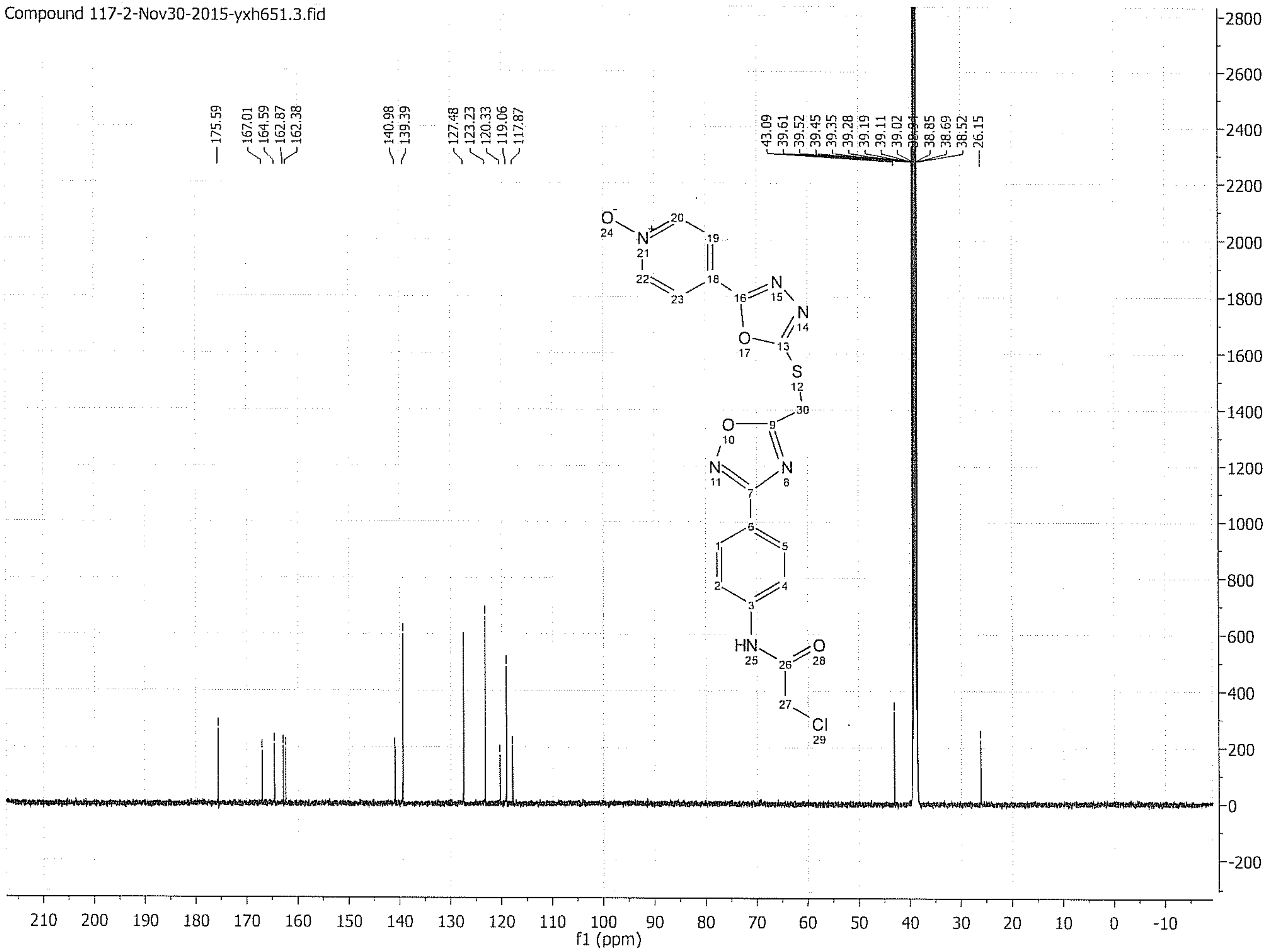
Compound 117-2-Nov30-2015-yxh651.3.fid
175.59
167.01
164.59
162.87
162.38
140.98
139.39
127.48
123.23
120.33
119.06
117.87
43.09
39.61
39.52
39.45
39.35
39.28
39.19
39.11
39.02
38.85
38.69
38.52
26.15
f1 (ppm)

Compound 117-2-Nov30-2015-yxh651.3.fid

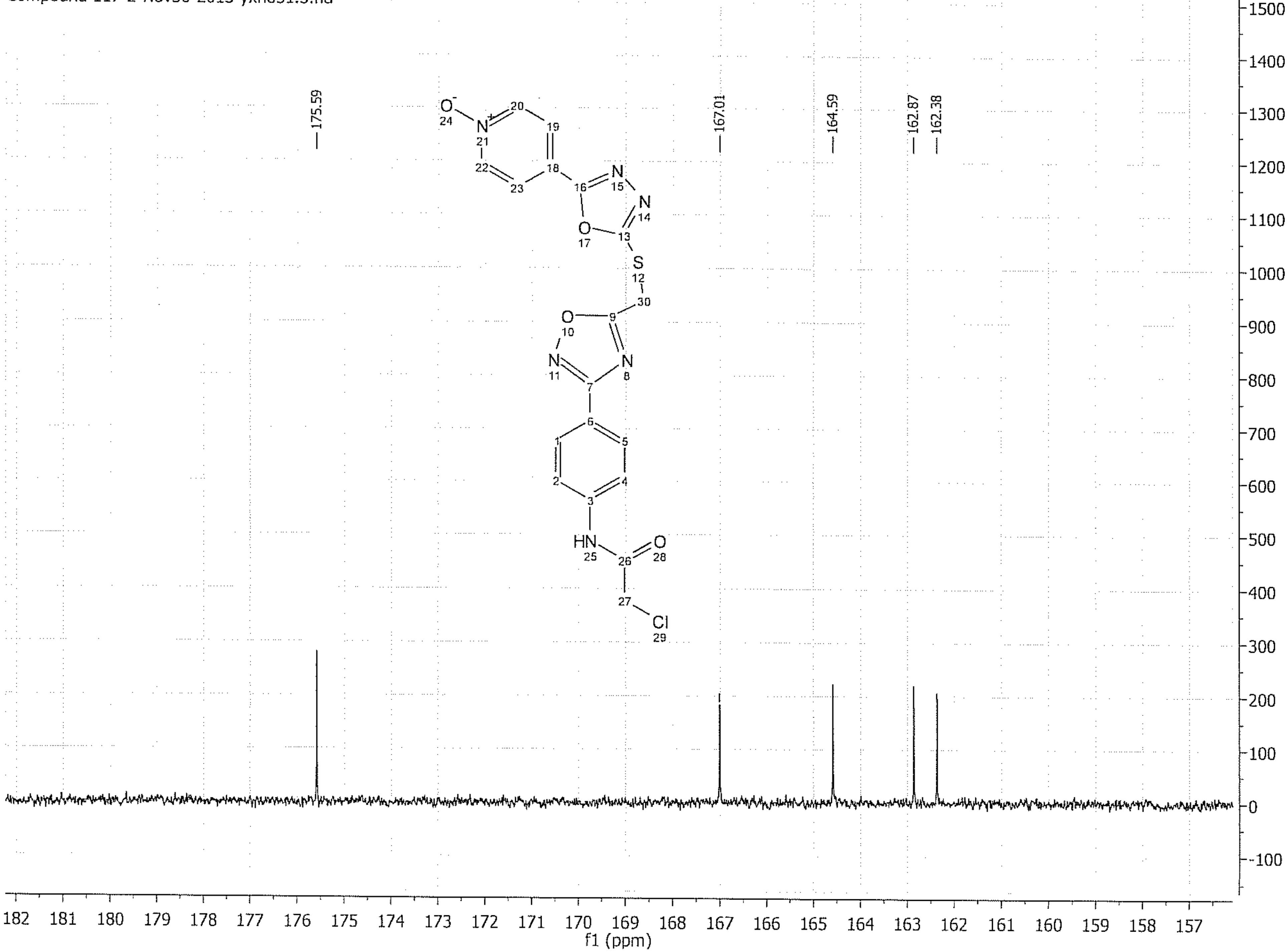

Compound 117-2-Nov30-2015-yxh651.3.fid

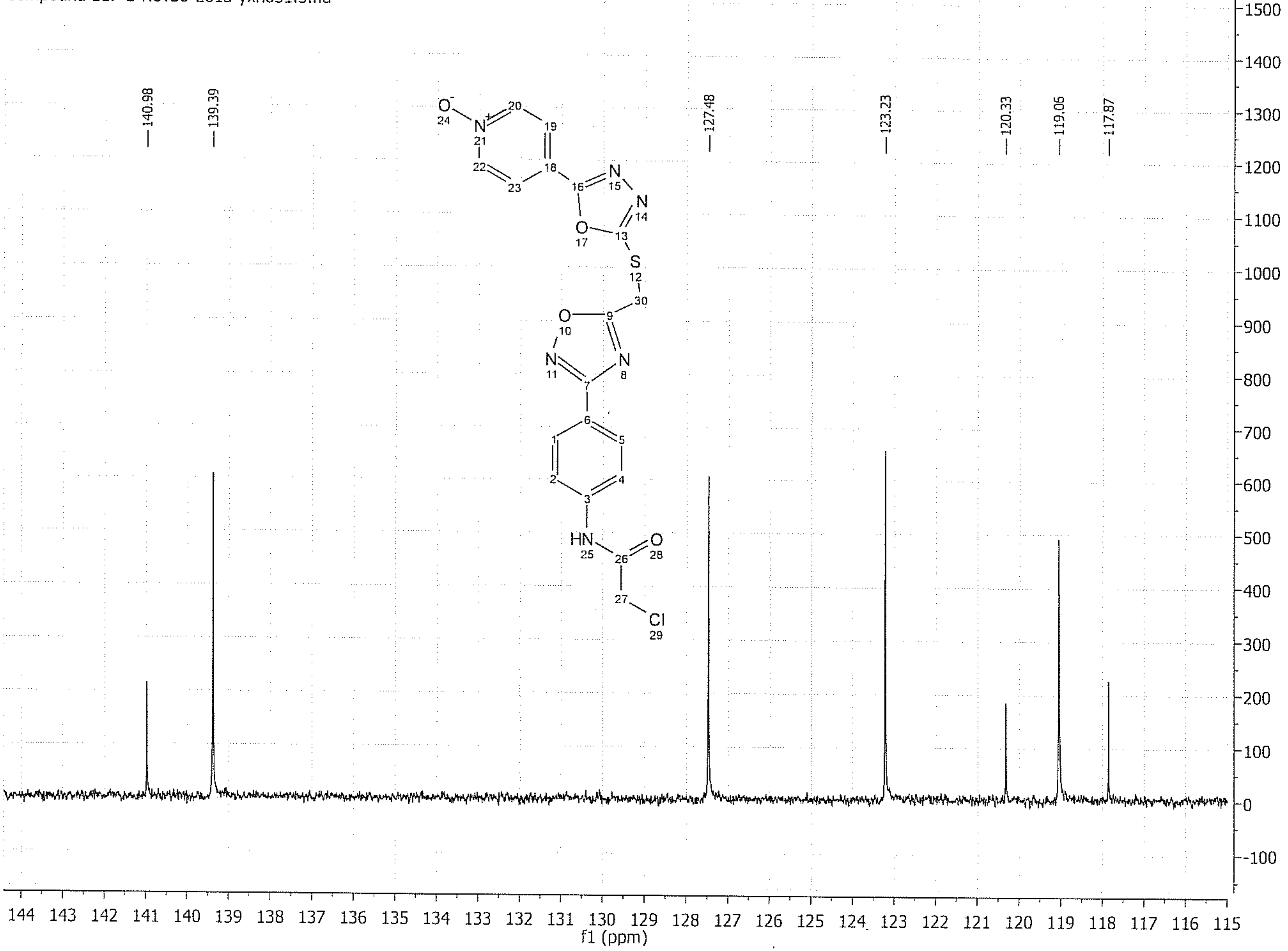

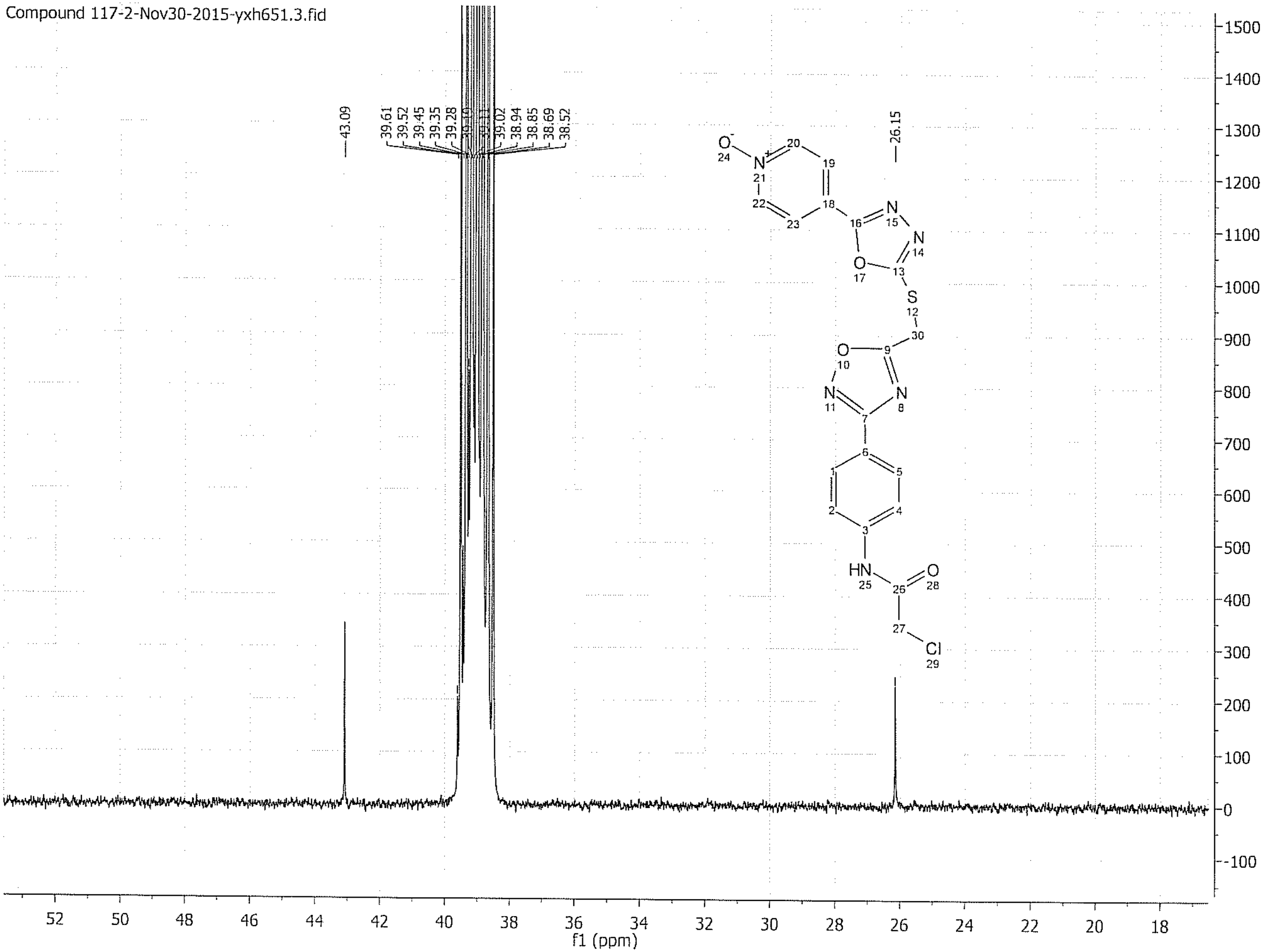

Compound 117-2-Nov30-2015-yxh651.3.fid
43.09
39.61
39.52
39.45
39.35
39.28
39.02
38.94
38.85
38.69
38.52
26.15
f1 (ppm)

Compound 117-2-Nov30-2015-yxh651.3.fid

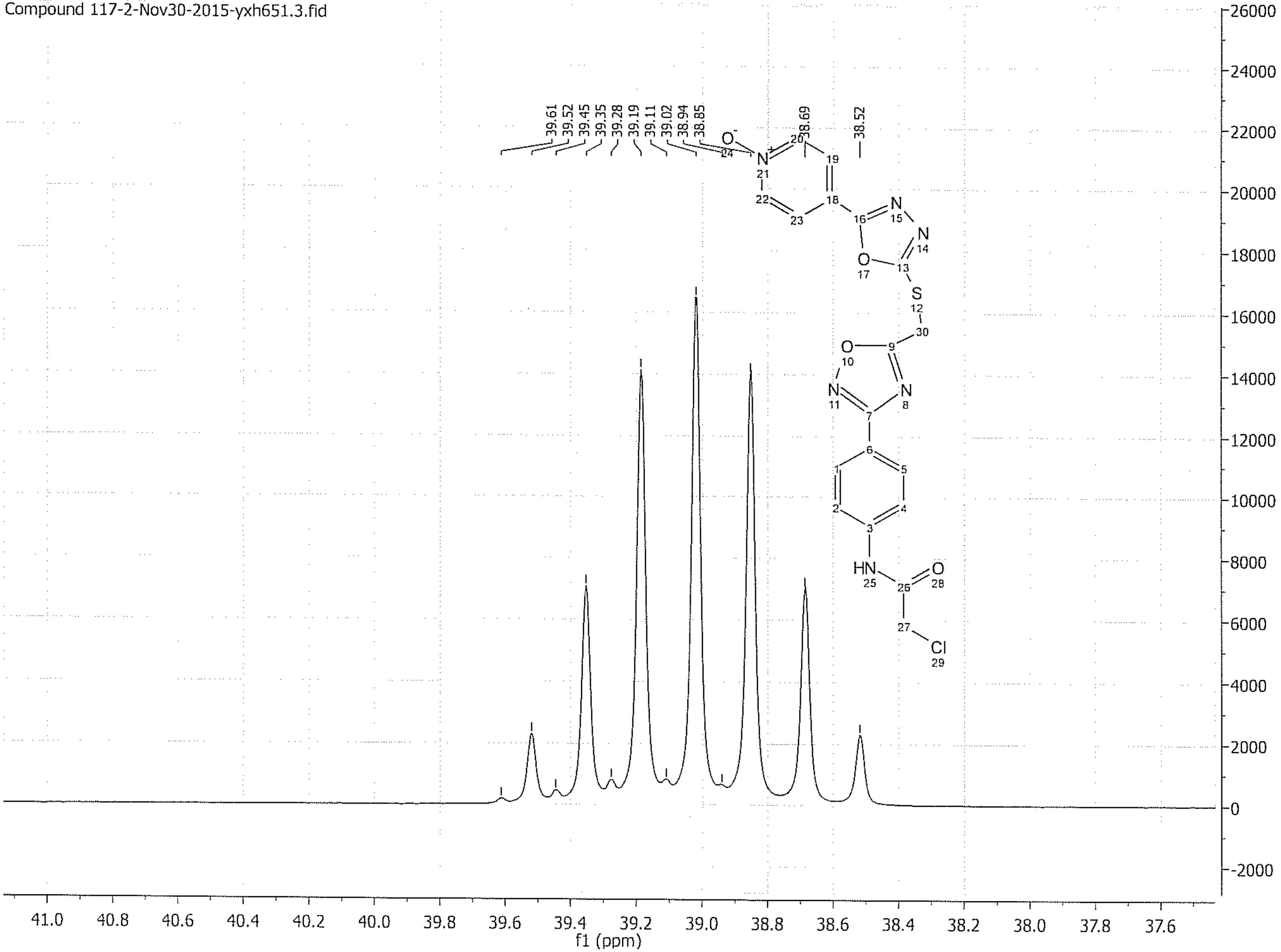

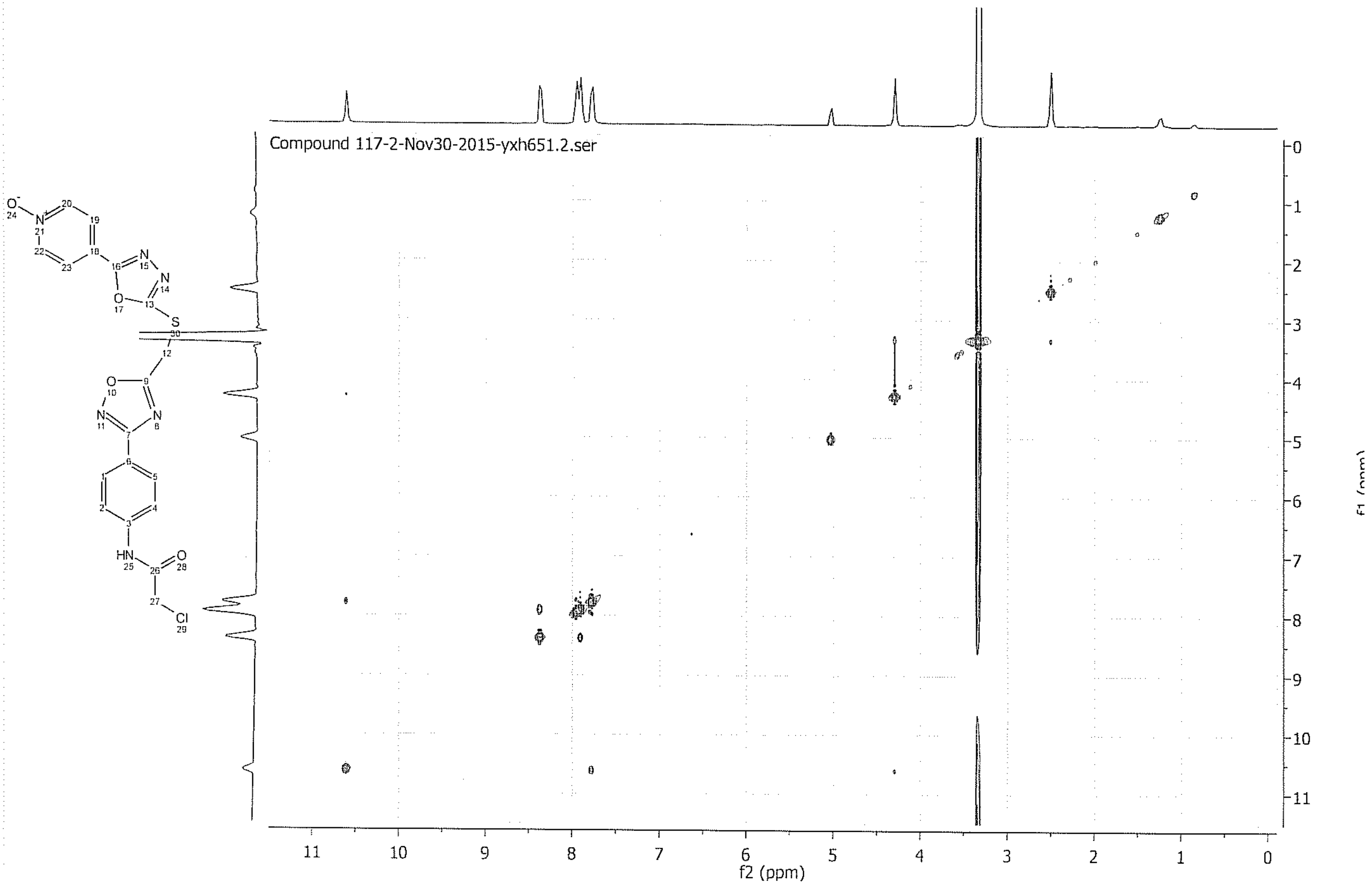
Compound 117-2-Nov30-2015-yxh651.2.ser
f2 (ppm)
f1 (ppm)
HN
O
Cl
S

Compound 117 HPLC-UV Purity Analysis

Instrument: Agilent 1200 Series,

Software: ChemStation Rev. B04.01 [481]

Column: Phenomenex Gemini 5µm, C18 110Å, 250x4.6 mm

Mobile Phase A: 95% water/5%Acetonitrile/0.1% TFA

Mobile Phase B: 95% Acetonitrile /5% water /0.1% TFA

Gradient:

Time	Mobile Phase B
0	30%
9	80%
12	80%
12.5	30%
15	30%

```
=====================================================================
Acq. Operator   : 117
Acq. Instrument : Instrument 1                    Location : Vial 63
Injection Date  : 12/1/2015 2:23:36 PM
                                               Inj Volume : 1 µl
Acq. Method     : C:\CHEM32\1\METHODS\GEORGE-242.M
Last changed    : 12/1/2015 2:22:12 PM by 117
                  (modified after loading)
Analysis Method : C:\CHEM32\1\METHODS\GEORGE-242.M
Last changed    : 12/2/2015 11:15:22 AM by 117
                  (modified after loading)
Method Info     : 117
```

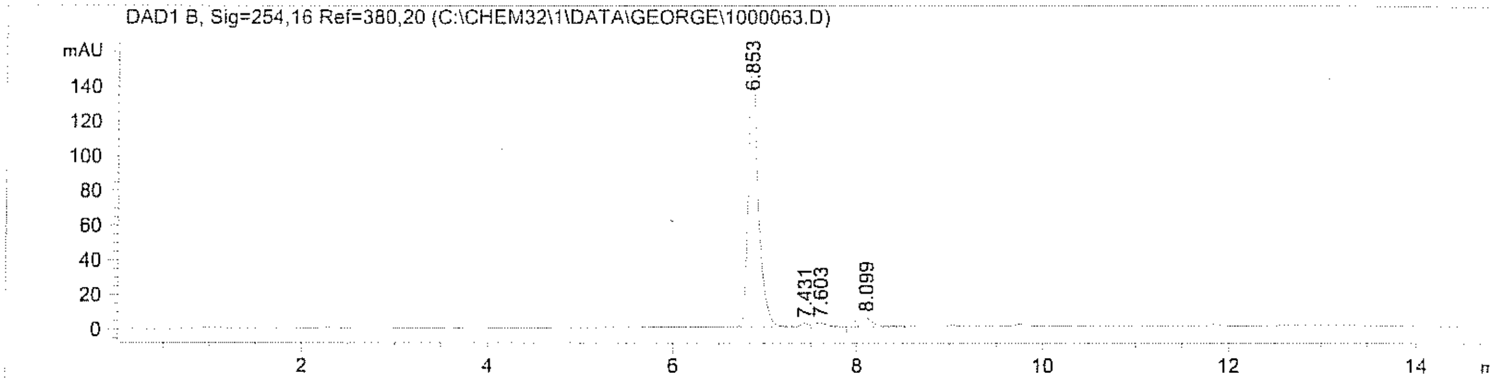

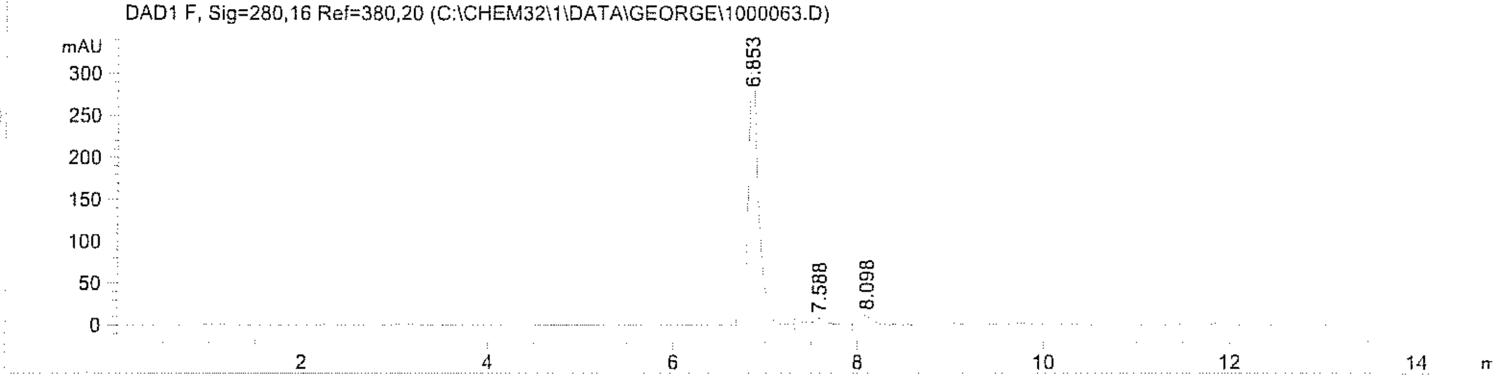


```
=====================================================================
                         Area Percent Report
=====================================================================

Sorted By             :      Signal
Multiplier:                   :      1.0000
Dilution:                     :      1.0000
Use Multiplier & Dilution Factor with ISTDs


Signal 1: DAD1 B, Sig=254,16 Ref=380,20
```

Peak #	RetTime [min]	Type	Width [min]	Area [mAU*s]	Height [mAU]	Area %
1	6.853	BV	0.1128	1170.61816	153.75114	93.4678
2	7.431	VV	0.0976	12.59015	1.99469	1.0053
3	7.603	VB	0.1531	23.83959	2.37460	1.9035
4	8.099	BB	0.1266	45.38136	5.36863	3.6235

Totals : 1252.42927 163.48907

D:\Xcalibur\data\Exactive\UKMSF\15-0366 10/28/2015 2:40:42 PM 117-2 C18H13ClN6O4S

15-0366 #2-15 RT: 0.05-0.39 AV: 14 NL: 2.14E8
T: FTMS + p ESI Full lock ms [150.00-1000.00]

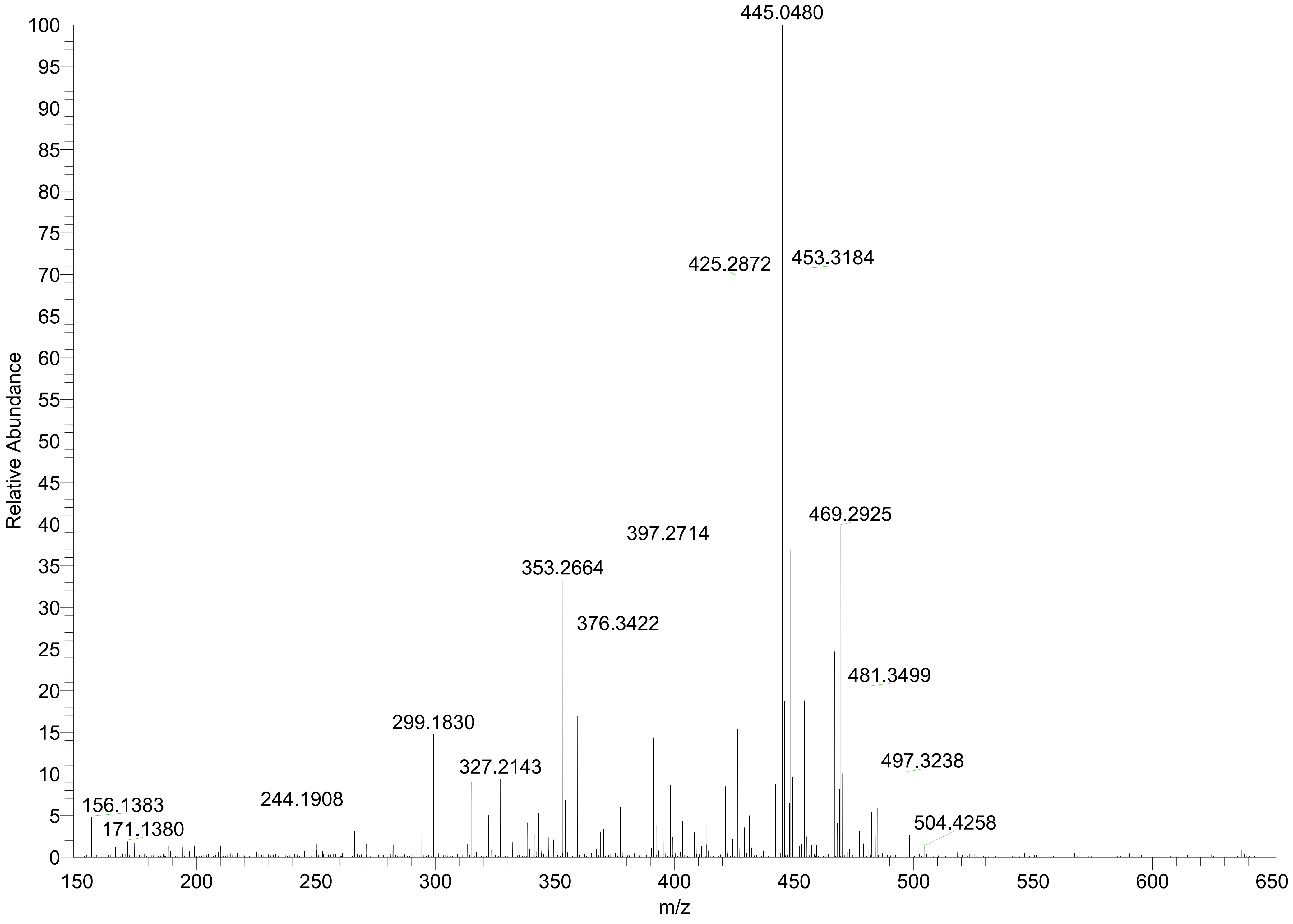

D:\Xcalibur\data\Exactive\UKMSF\15-0366 10/28/2015 2:40:42 PM 117-2 C18H13ClN6O4S

15-0366 #2-15 RT: 0.05-0.39 AV: 14 NL: 2.14E8
T: FTMS + p ESI Full lock ms [150.00-1000.00]

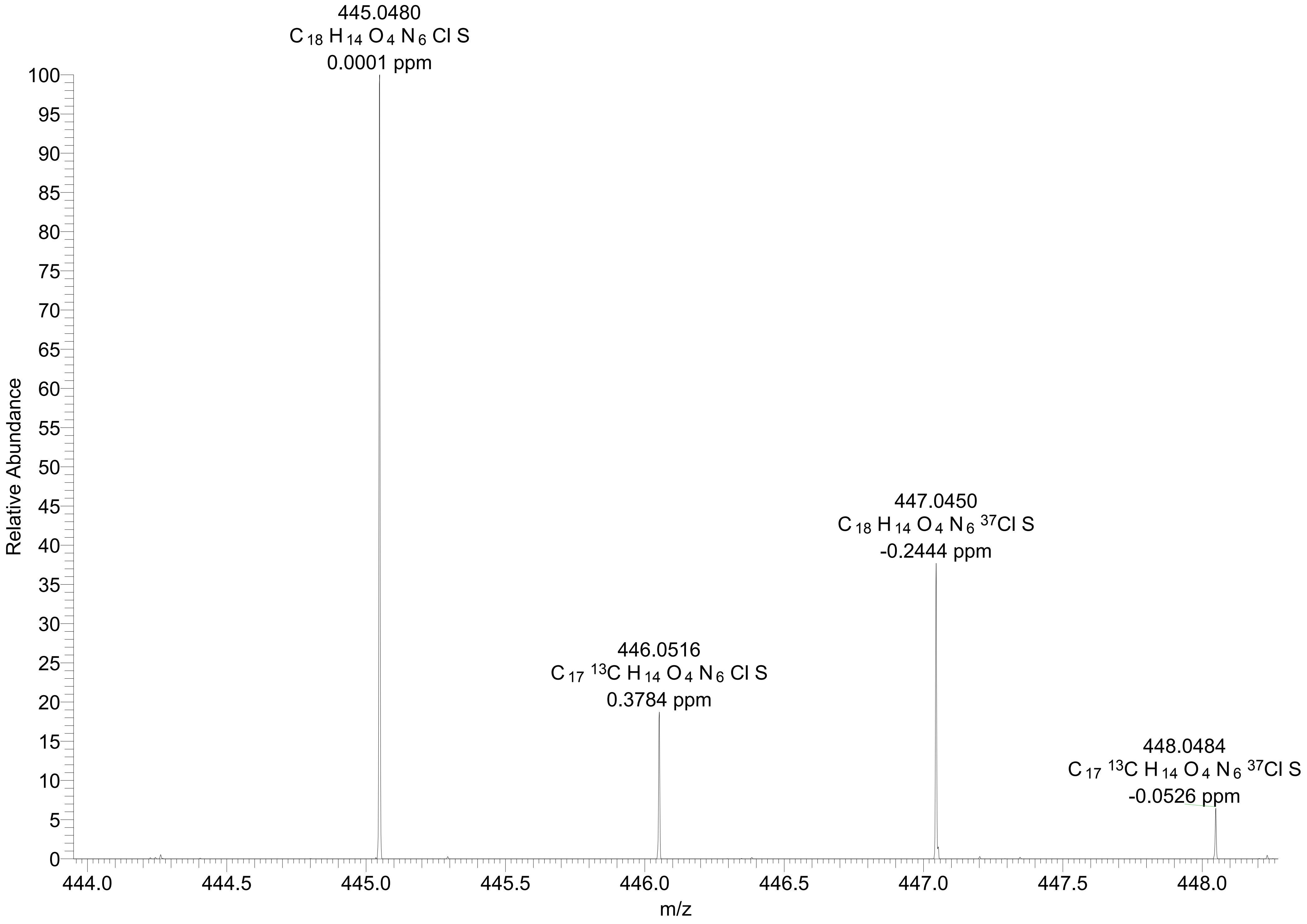

15-0366#2-15 RT: 0.05-0.39 AV: 14
T: FTMS + p ESI Full lock ms [150.00-1000.00]
m/z= 443.9502-448.2720

m/z	Intensity	Relative	Theo. Mass	Delta (ppm)	RDB equiv.	Composition
445.0480	221805856.0	100.00	445.0480	0.00	14.5	$C_{18} H_{14} O_4 N_6 Cl S$
446.0516	40903596.0	18.44	446.0514	0.38	14.5	$C_{17} {}^{13}C H_{14} O_4 N_6 Cl S$
447.0450	82022112.0	36.98	447.0451	-0.24	14.5	$C_{18} H_{14} O_4 N_6 {}^{37}Cl S$
448.0484	13975932.0	6.30	448.0484	-0.05	14.5	$C_{17} {}^{13}C H_{14} O_4 N_6 {}^{37}Cl S$

D:\Xcalibur\data\Exactive\UKMSF\15-0366 10/28/2015 2:40:42 PM 117-2 C18H13ClN6O4S

15-0366 #265 RT: 6.95 AV: 1 NL: 1.57E8
T: FTMS - p ESI Full lock ms [100.00-1000.00]

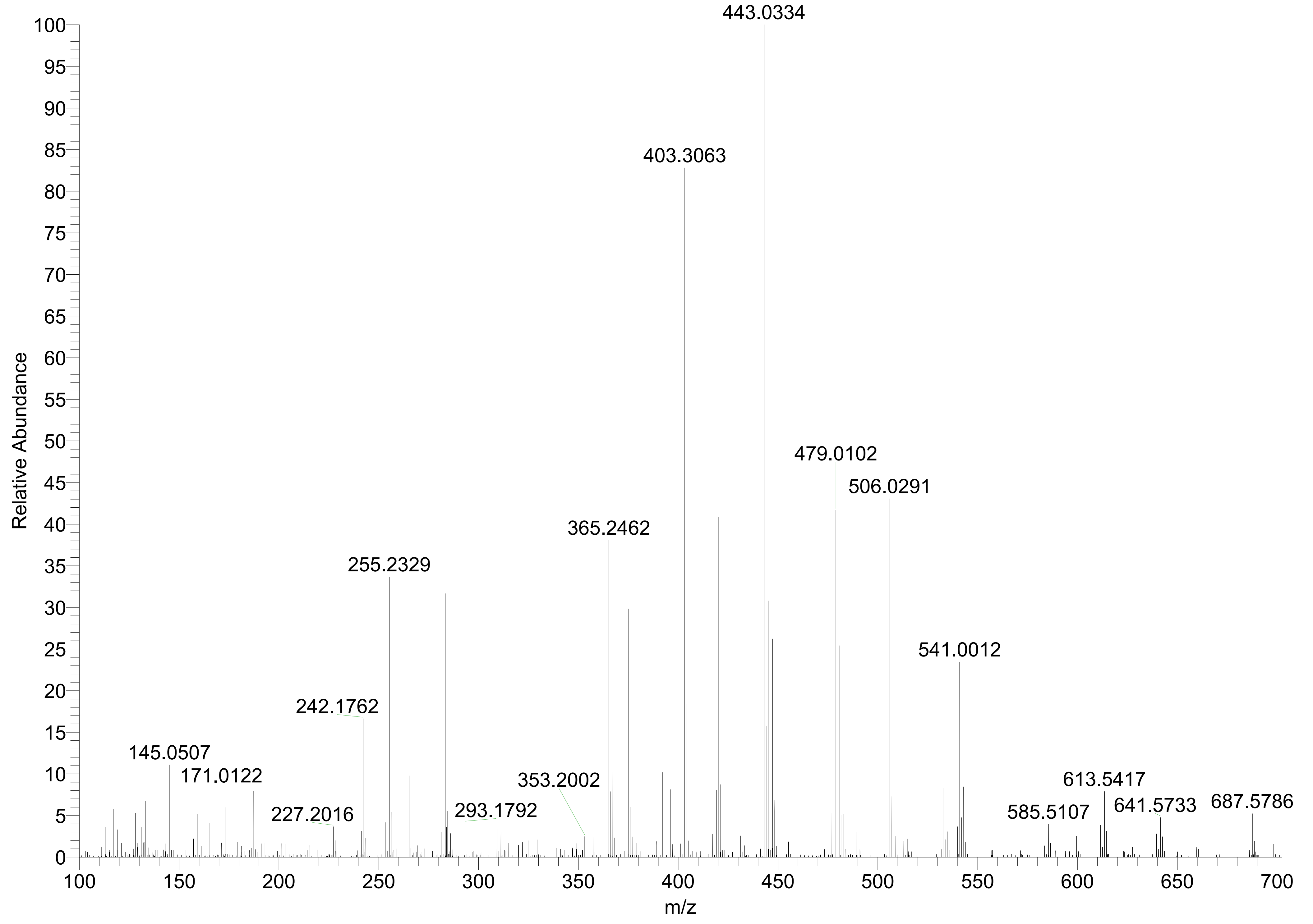

D:\Xcalibur\data\Exactive\UKMSF\15-0366 10/28/2015 2:40:42 PM 117-2 C18H13ClN6O4S

15-0366 #265 RT: 6.95 AV: 1 NL: 1.57E8
T: FTMS - p ESI Full lock ms [100.00-1000.00]

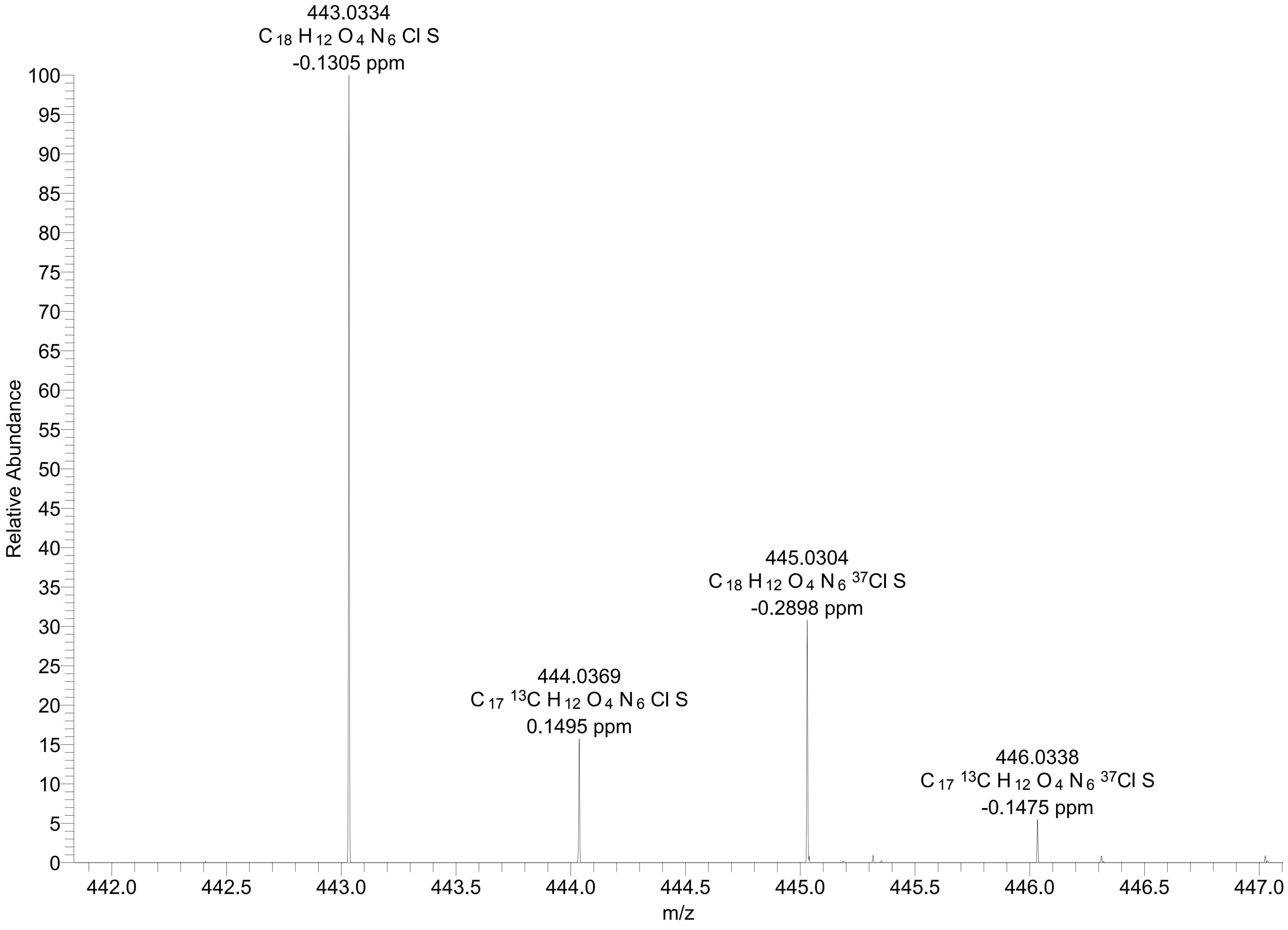

15-0366#265 RT: 6.95
T: FTMS - p ESI Full lock ms [100.00-1000.00]
m/z= 441.8346-447.1034

m/z	Intensity	Relative	Theo. Mass	Delta (ppm)	RDB equiv.	Composition
443.0334	158444928.0	100.00	443.0335	-0.13	15.5	$C_{18}H_{12}O_4N_6ClS$
444.0369	25300164.0	15.97	444.0368	0.15	15.5	$C_{17}{}^{13}CH_{12}O_4N_6ClS$
445.0304	48780188.0	30.79	445.0305	-0.29	15.5	$C_{18}H_{12}O_4N_6{}^{37}ClS$
446.0338	8847348.0	5.58	446.0339	-0.15	15.5	$C_{17}{}^{13}CH_{12}O_4N_6{}^{37}ClS$
